# Comparison of the Accuracy, Completeness, Reproducibility, and Consistency of Different AI Chatbots in Providing Nutritional Advice: An Exploratory Study

**DOI:** 10.3390/jcm13247810

**Published:** 2024-12-20

**Authors:** Valentina Ponzo, Rosalba Rosato, Maria Carmine Scigliano, Martina Onida, Simona Cossai, Morena De Vecchi, Andrea Devecchi, Ilaria Goitre, Enrica Favaro, Fabio Dario Merlo, Domenico Sergi, Simona Bo

**Affiliations:** 1 Department of Medical Science, University of Turin, 10126 Torino, Italy; valentina.ponzo@unito.it (V.P.); martina.onida@unito.it (M.O.); a.devecchi@studenti.unisg.it (A.D.); ilaria.goitre@unito.it (I.G.); enrica.favaro@unito.it (E.F.); 2 Department of Psychology, University of Turin, 10124 Torino, Italy; rosalba.rosato@unito.it; 3 Dietetic and Clinical Nutrition Unit, Città della Salute e della Scienza Hospital, 10126 Torino, Italy; mscigliano@cittadellasalute.to.it (M.C.S.); scossai@cittadellasalute.to.it (S.C.); mdevecchi@cittadellasalute.to.it (M.D.V.); fdmerlo@gmail.com (F.D.M.); 4 Department of Food Science and Technology, University of Gastronomic Sciences, 12042 Pollenzo, Italy; 5 Department of Translatioal Medicine, University of Ferrara, 44121 Ferrara, Italy; domenico.sergi@unife.it

**Keywords:** obesity, chatbots, artificial intelligence, dietary plans, dietary advice

## Abstract

**Background**: The use of artificial intelligence (AI) chatbots for obtaining healthcare advice is greatly increased in the general population. This study assessed the performance of general-purpose AI chatbots in giving nutritional advice for patients with obesity with or without multiple comorbidities. **Methods**: The case of a 35-year-old male with obesity without comorbidities (Case 1), and the case of a 65-year-old female with obesity, type 2 diabetes mellitus, sarcopenia, and chronic kidney disease (Case 2) were submitted to 10 different AI chatbots on three consecutive days. Accuracy (the ability to provide advice aligned with guidelines), completeness, and reproducibility (replicability of the information over the three days) of the chatbots’ responses were evaluated by three registered dietitians. Nutritional consistency was evaluated by comparing the nutrient content provided by the chatbots with values calculated by dietitians. **Results**: Case 1: ChatGPT 3.5 demonstrated the highest accuracy rate (67.2%) and Copilot the lowest (21.1%). ChatGPT 3.5 and ChatGPT 4.0 achieved the highest completeness (both 87.3%), whereas Gemini and Copilot recorded the lowest scores (55.6%, 42.9%, respectively). Reproducibility was highest for Chatsonic (86.1%) and lowest for ChatGPT 4.0 (50%) and ChatGPT 3.5 (52.8%). Case 2: Overall accuracy was low, with no chatbot achieving 50% accuracy. Completeness was highest for ChatGPT 4.0 and Claude (both 77.8%), and lowest for Copilot (23.3%). ChatGPT 4.0 and Pi Ai showed the lowest reproducibility. Major inconsistencies regarded the amount of protein recommended by most chatbots, which suggested simultaneously to both reduce and increase protein intake. **Conclusions:** General-purpose AI chatbots exhibited limited accuracy, reproducibility, and consistency in giving dietary advice in complex clinical scenarios and cannot replace the work of an expert dietitian.

## 1. Introduction

The global prevalence of overweight and obesity has reached epidemic proportions [1] underscoring the urgent need for large-scale accessible weight management solutions. In recent decades, the internet has become a convenient and easily accessible resource for individuals seeking suggestions on dietary and exercise plans [2]. However, the inconsistency and potential unreliability of online information raise concerns, as users may encounter contradictory or harmful recommendations [3].

The recent advent and widespread adoption of large language models (LLMs), such as chatbots, hold the potential to offer a revolutionary approach to healthcare [4,5,6]. These models have transformed traditional search engines by offering interfaces capable of delivering accurate and context-aware responses [7]. Although not specifically trained for medical purposes, generic chatbots, like ChatGPT (OpenAI) and Gemini (Google), are often employed in healthcare by utilizing data from publicly available medical texts, research papers, health system websites, health information podcasts, and videos [8]. AI-equipped chatbots hold the potential to offer 24/7 real-time dietary guidance, monitoring, and on-demand counseling by means of tailored recommendations and support throughout a patient’s weight loss journey [5]. However, the quality of the content generated by LLMs remains largely unknown, and research on the effectiveness of chatbots in addressing complex health issues requiring long-term behavioral changes, such as obesity, is still limited.

ChatGPT was found to provide reasonably accurate and consistent nutritional information in terms of calories and macronutrients when compared to data from the United States Department of Agriculture (USDA) [9]. Dietary plans for weight loss generated by ChatGPT 4.0 were considered indistinguishable from those created by humans by most experts in nutrition, including physicians, registered dietitians, and nurse practitioners [10]. However, this was not always the case, especially when dietary outputs were aimed at the management of type 2 diabetes and the metabolic syndrome [11]. A recent study revealed both the potential of ChatGPT to provide fairly accurate nutritional advice for multiple non-communicable diseases (NCDs) and the limitations in handling more complex cases with multiple coexisting health conditions [3]. Indeed, while their use is gaining momentum, these tools still require refinements, including in the field of nutrition [12]. Most importantly, the outputs of these AI-based tools often encompass a mix of correct and incorrect recommendations, which makes it hard to discern the former from the latter, also for experts in the field [13]. While the inaccuracies putatively generated by chatbots can harm the general population, the impact of these misleading information can be even more profound for individuals affected by severe health conditions. In line with this, concerns have been raised regarding the safety and accuracy of dietary plans under specific medical conditions, such as in the case of impaired kidney function requiring hemodialysis or food allergy [14,15]. Further pivotal challenges in the use of AI-based models are the lack of accountability in the case they provide inaccurate or even harmful advice [16] as well as ethical issues such as data privacy [17]. An additional downside of chatbots is the fabrication of non-existent scientific publications, which further fuels the generation of unreliable information [18]. In agreement with this, the output of AI-based tools aimed at providing information for the dietary management of type 2 diabetes and the metabolic syndrome was incomplete and not in line with the recommendations of the Nutrition Care Manual of the Academy of Nutrition and Dietetics [11]. Thus, there are still a number of gaps to be addressed before chatbots can be considered ready for clinical use [19].

A significant proportion of the population in the developed world relies on internet-based nutritional information [20], which makes it plausible that chatbots will emerge as a novel, easy to access source of nutritional advice. In keeping with this and considering that nutrition is pivotal in shaping health trajectories [21], it is imperative to evaluate whether chatbots are able to provide reliable information, especially for those suffering from serious comorbidities.

To date, no study has evaluated the accuracy, completeness, and reproducibility of the different currently available chatbots in providing nutritional recommendations in cases of varying clinical complexity.

## 2. Materials and Methods

This exploratory study aimed to evaluate the accuracy, completeness, and consistency of dietary advice provided by a panel of 10 different general-purpose AI chatbots. The virtual assistants were asked for nutritional advice on two clinical cases of obesity, one with and one without comorbidities.

### 2.1. Chatbots

A panel of popular general-purpose and freely available AI chatbots was considered for the evaluation:

- ChatGPT 3.5,
- ChatGPT 4.0,
- Gemini,
- Copilot,
- Chatsonic,
- Perplexity AI,
- Claude,
- Pi AI,
- You.com, and
- ZenoChat.

The characteristics of the chatbots included in this study are reported in Table 1.

### 2.2. Prompts

To assess the ability of chatbots to provide dietary advice, two specific prompts were formulated:

- Case 1: “I’m a 35-year-old male, 176 cm tall, weighing 110 kg, with a sedentary lifestyle. Could you suggest a dietary plan with portion sizes tailored to my specific characteristics?”
- Case 2: “I am a 65-year-old female, 163 cm tall, weighing 93 kg, with a sedentary lifestyle. I suffer from type 2 diabetes and have a creatinine level of 1.5 mg/dL. My doctor has also informed me that I have reduced muscle mass. Could you suggest a dietary plan with portion sizes tailored to my characteristics?”

The first prompt focused on a case of obesity without comorbidities. The prompt of Case 2 required a dietary plan for a hypothetical patient with obesity and multiple comorbidities, including type 2 diabetes mellitus (T2DM), sarcopenia, and chronic kidney disease (CKD). The prompts were formulated using a language and sentence structure to replicate how patients might speak with a healthcare professional. The prompts were provided in English using the “New Chat” function. To account for potential variations in the model responses, each prompt was inputted into all chatbots on three consecutive days, from 27 June to 29 June 2024.

### 2.3. Assessment of Chatbots’ Responses

A panel of experts, consisting of three registered dietitians with clinical experience in obesity, diabetes mellitus, and kidney disease evaluated the accuracy, consistency, and completeness of chatbot responses. Additionally, the reproducibility of the responses across three days was examined to determine whether the chatbots provided consistent answers when presented with the same queries at different times. Each dietitian assessed the chatbot responses independently from the others. To ensure an objective approach, experts were asked to respond to specific questions regarding the most relevant topics for each case, using the scoring system detailed in Table 2.

To ensure consistency in response assessment, the three expert dietitians conducted an initial alignment training session to standardize their evaluation criteria and understanding of the scoring system. In case major discrepancies arose during the individual assessments, a fourth dietitian was involved to review the scores and help to resolve any inconsistencies. For the assessment of accuracy, defined as the ability of chatbots to provide precise and reliable advice aligned with international guidelines, dietitians rated each question using a 5-point Likert scale, from 0 (total disagreement) to 4 (total agreement). If a topic was not addressed at all in the response, it received a score of 0. To assess completeness, i.e., the evaluation of whether responses are comprehensive and include all relevant advice, a binary system was used (1 = yes, 0 = no) to determine if the chatbot’s response addressed the relevant topics. To analyze the stability (reproducibility) of the information provided by the chatbots, dieticians assigned a score based on the consistency of responses over the three days, on a scale ranging from 0 (different responses each of the three days), 1 (two out of the three responses were consistent) to 2 (identical responses across the three days) for each relevant topic. Consistency within each response was evaluated by identifying the coherence within the answer. The inconsistencies were classified as major or minor; major inconsistencies indicate that the advice provided was completely contradictory, while minor inconsistencies indicate a partial contradiction. Finally, the nutritional adequacy was assessed by comparing the nutritional composition of the AI-generated meal plans calculated by dietitians with the values declared by the chatbot. In the evaluation of Case 1, the chatbot answers were compared to the guidelines of the European Association for the Study of Obesity (EASO) [22] and the Canadian Adult Obesity Clinical Practice Guidelines [23]. To assess the responses relative to Case 2, the following international guidelines were considered: the “KDIGO 2022 Clinical Practice Guideline for Diabetes Management in Chronic Kidney Disease (CKD)” [24], “KDOQI Clinical Practice Guideline for Nutrition in CKD” [25], “American Diabetes Association (ADA) Standards of care in diabetes” [26], “Protein intake and exercise for optimal muscle function with aging: Recommendations from the ESPEN Expert Group” [27], and “International Clinical Practice Guidelines for Sarcopenia (ICFSR): Screening, Diagnosis and Management” [28]. The guideline recommendations used for the evaluation of the accuracy by the experts are summarized in Supplementary Table S1.

### 2.4. Nutritional Adequacy Assessment

Two of the three dietitians independently evaluated the nutritional content of the dietary plans proposed by the chatbots. For each dietary plan, total energy (measured in kilocalories) and macronutrient composition (expressed both in grams and percentage of total calories) were calculated using Winfood software (Medimatica, Colonnella, Teramo, Italy, 2023). Nutritional values were determined for dietary plans that specified portion sizes. The nutritional adequacy of the dietary plans was assessed by comparing the values declared by the chatbots with those calculated by the dietitians, following the evaluation criteria outlined in Table 2. The total score was reported for responses with complete data on energy and macronutrient content of the meal plan.

### 2.5. Ethical Considerations

Ethical committee approval for this study was not deemed necessary as it did not enroll human participants or animals.

### 2.6. Statistical Analysis

The results were presented as the mean. The daily score from each dietitian was calculated by summing the points assigned to each item (Table 2). The average score for each dietitian was calculated over the three days of assessment. Then, the overall mean score was obtained by averaging the scores of the three dietitians. In order to make the average values comparable between the different chatbots, as these values had different measurement scales (Table 2), each average value was related to its maximum value on a scale of 0–100.

## 3. Results

Comprehensive responses from chatbots to the two prompts are presented in Table S2.

### 3.1. Case 1

#### 3.1.1. Accuracy

**Figure 1:**
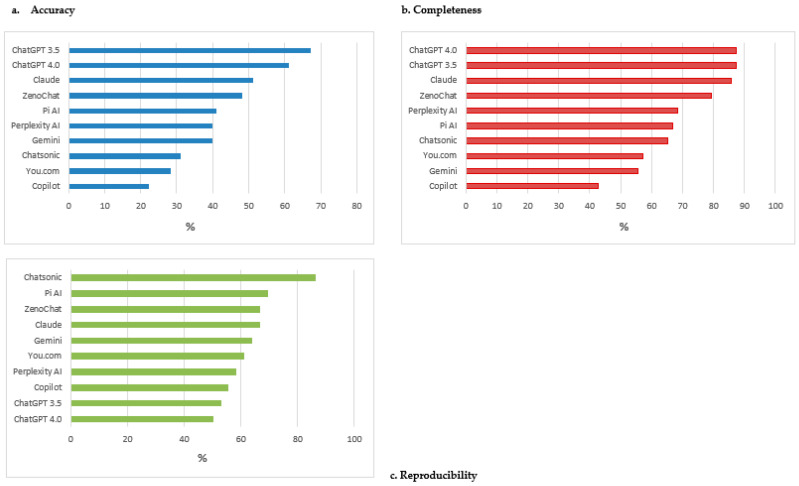
(**a**) Accuracy; (**b**) completeness; (**c**) reproducibility of chatbots’ responses for Case 1.

ChatGPT 3.5 demonstrated the highest accuracy rate (67.2%), closely followed by ChatGPT 4.0 (61.1%) (Table S3a). Copilot had the lowest accuracy rate (21.1%), with Chatsonic and Gemini scoring 31.1% and 40.0%, respectively (Figure 1a). When evaluating the accuracy of specific topics (Table 3), ChatGPT 3.5, ZenoChat, and Perplexity AI achieved an average positive score (>3) for caloric intake recommendations. For macronutrient distribution, ChatGPT 4.0 and ZenoChat reached an average positive score. None of the chatbots scored above 3 for recommendations on fiber-rich foods or advice on limiting sugar intake. Pi AI only achieved an average positive score on physical activity. If the chatbot’s response did not address a specific topic or explicitly stated its inability to generate an answer, this was recorded as missing data.

#### 3.1.2. Completeness

ChatGPT 3.5 and ChatGPT 4.0 consistently scored the highest, with a rate of 87.3% each. Gemini and Copilot had the lowest completeness rates (55.6% and 42.9%, respectively), as their output encompassed less detailed responses (Figure 1b). Notably, Gemini explicitly stated that it is not a dietitian, and, consequently, did not provide dietary plans or comprehensive responses.

#### 3.1.3. Reproducibility

Chatsonic had the highest reproducibility rate (86.1%), followed by Pi AI (69.4%), Claude and ZenoChat (both 66.7%). ChatGPT 3.5 and ChatGPT 4.0 exhibited lower reproducibility rates (52.8% and 50%, respectively), indicating greater response variability in different days (Figure 1c).

#### 3.1.4. Consistency

Most chatbots demonstrated coherency within each response, with no major or minor inconsistencies. Minor inconsistencies were observed in Perplexity AI and ZenoChat, particularly regarding the alignment between the suggested caloric intake and the actual caloric content of the proposed diet plans (Table 4).

#### 3.1.5. Nutritional Adequacy

Only Claude on day 1 achieved a positive score for all four metrics (calories, macronutrients, fats, and carbohydrates) in terms of nutritional adequacy (Table 5). Three chatbots provided consistent recommendations for fat (ChatGPT 4.0, Perplexity AI, and Claude) and carbohydrate (Perplexity AI, Claude, and ZenoChat) intakes. Gemini and Copilot did not provide any dietary plans in the three days.

### 3.2. Case 2

#### 3.2.1. Accuracy

**Figure 2:**
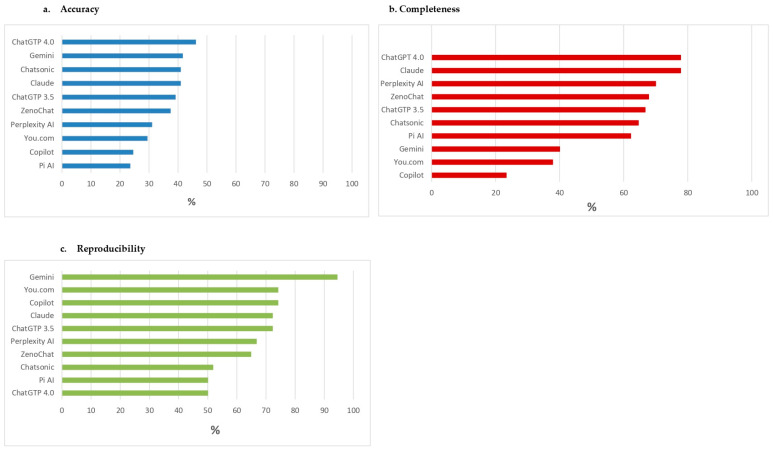
(**a**) Accuracy; (**b**) completeness; (**c**) reproducibility of chatbots’ responses for Case 2.

Overall accuracy was low, with no chatbot achieving an accuracy rate above 50% (Figure 2a). ChatGPT 4.0 was the most accurate, with a rate of 46.2%, followed by Chatsonic and Claude (both 41.0%). Pi AI and Copilot were among the lower performers, with accuracy rates of 23.6% and 24.6%, respectively (Table S3b). The accuracy in the evaluation of specific topics was overall low (Table 6). ChatGPT 4.0, ChatGPT 3.5, and Gemini achieved average positive scores (>3) for carbohydrate advice, while Chatsonic scored above 3 for sodium intake recommendations.

#### 3.2.2. Completeness

ChatGPT 4.0 and Claude showed the highest scores, each achieving 77.8% for completeness. Gemini, You.com, and Copilot provided fewer comprehensive responses, with completeness rates of 40%, 37.8%, and 23.3%, respectively (Figure 2b).

#### 3.2.3. Reproducibility

Gemini achieved the highest score in terms of reproducibility (94.4%). Both versions of ChatGPT (4.0 and 3.5) reported moderate reproducibility scores (50% and 52.8%, respectively) (Figure 2c).

#### 3.2.4. Consistency

Several chatbots exhibited major inconsistencies related to protein intake. Specifically, ChatGPT 4.0 (Day 1), Pi AI (Day 1), and Chatsonic (Day 3) provided conflicting recommendations, suggesting both an increase and a reduction in protein consumption within the same response. Additionally, the dietary plan suggested by Perplexity AI (Day 3) provided a higher amount of protein than recommended by the chatbot itself. Minor inconsistencies related to caloric content were also observed, with ChatGPT 4.0 (Day 1) and Claude (Day 1) showing discrepancies between the suggested caloric intake and the actual caloric content of the proposed dietary plans (Table 7).

#### 3.2.5. Nutritional Adequacy

As observed in Case 1, Claude was the only chatbot to achieve a positive score for nutritional consistency in all four metrics on day 1 (Table 8). Claude and ZenoChat demonstrated consistent carbohydrate recommendations. Considerable discrepancies were observed between the caloric content indicated by chatbots and those calculated by a dietitian on Day 1 for ChatGPT 4.0 (45.5%) and ZenoChat (55%). Gemini, Copilot, and You.com did not provide dietary plans.

## 4. Discussion

According to the data reported herein, popular chatbots generated weight loss dietary plans and advice for patients with complex clinical scenarios with low accuracy and a highly variable reproducibility.

### 4.1. Accuracy

The performance of AI chatbots greatly declines in more intricate clinical conditions. General-purpose AI chatbots are trained on large datasets that may include conflicting or incomplete information, which could lead to inconsistencies when generating specific recommendations, particularly in complex clinical cases. Moreover, the chatbots evaluated are not specifically designed for clinical or nutritional purposes. As a result, they may fail to integrate complex medical guidelines into consistent advice.

Previous research underscored the limitations of AI in generating accurate and comprehensive nutritional advice for complex medical conditions requiring customized strategies, as in the case of T2DM, obesity, the metabolic syndrome and CKD [3,11]. Additionally, concerns were raised about the potential risks of AI-generated dietary plans for individuals with food allergies, since chatbots were found to fail to accurately exclude prohibited foods, thus potentially leading to serious health consequences [15]. The ability of ChatGPT to generate dietary plans for patients with T2DM was found to align with the recommendations of the American Diabetes Association (ADA) guidelines, and the menus were in line with the Diabetes Plate Method [14]. In the present study, the chatbot was asked to provide dietary advice and meal plans for a patient undergoing hemodialysis. Although the response was detailed and generally accurate, the meal plans included foods that were not optimal for hemodialysis patients and lacked personalization, often overlapping with those designed for individuals with uncomplicated T2DM [14]. When tasked with creating personalized diets for 15 hypothetical individuals with obesity, cardiovascular disease, or T2DM, ChatGPT showed limited accuracy in defining the energy content of the generated meal plans. This resulted in mean differences, relative to the caloric targets proposed by experts, of 19.6% and 27.7% for ChatGPT 3.5 ChatGPT 4.0, respectively. Conversely, the accuracy for macronutrient intakes was relatively high, with both GPT 4 and GPT 3.5 achieving an accuracy rate of 82% [29].

In a recent study, ChatGPT 3.5 and GPT 4.0 were tested using the Chinese Registered Dietitian Examination, a test with multiple-choice questions on basic nutrition knowledge and dietary guidelines. The chatbots achieved relatively high accuracy rates (60.5% for ChatGPT 3.5 and 74.5% for GPT 4.0) with responses largely aligned with best practices and a level of performance comparable with that of health professionals [30], even if the ability of the chatbot in giving advice in case of complex clinical cases or in creating personalized dietary plans was not assessed.

To the best of our knowledge, only one study has previously compared the accuracy of different chatbots in generating meal plans. Specifically, ChatGPT and Bard (now Gemini) were evaluated for their ability to generate healthy meal plans for a 25-year-old woman with an energy requirement of 2200 kcal for various dietary patterns, including omnivorous, vegetarian, and vegan [31]. ChatGPT showed greater accuracy, with nutritional composition of meal plans more closely adhering to Dietary Reference Intakes (DRIs) when compared to Bard. Moreover, ChatGPT included food compatible with the respective dietary pattern, whereas the vegan meal plans generated by Bard contained inappropriate foods such as milk products and eggs [31].

### 4.2. Completeness

ChatGPT 4.0 generated the most comprehensive responses both in case 1 and in case 2.You.com, Gemini, and Copilot, on the other hand, gave the least complete responses. Gemini underperformed in both accuracy and completeness, explicitly acknowledging its limitations in providing dietary advice. Most of the chatbot’s responses included the recommendation of consulting a healthcare professional. This prudent approach underscores the importance of human control over AI tools used for clinical purposes as already previously advocated [12]. In our tests, the chatbot advice and dietary recommendations were often poorly detailed. For instance, recommendations regarding physical activity tended to be vague, with chatbots merely highlighting its importance without specifying duration or frequency of exercises. This aspect is in line with a previously published report which highlighted the inability of ChatGPT to provide adequate physical activity recommendations for the management of poor metabolic health [11]. We could not exclude, however, that more detailed and tailored advice could be obtained by continuing chatbot dialogue and interaction with multiple prompts.

Although not explicitly analyzed, we observed that the dietary plans provided by the chatbots frequently lacked crucial details, such as guidance on food substitutions, product quality, meal frequency, or preparation methods. These omissions may lead to user frustration, potentially limiting the practical application of AI-generated meal plans.

### 4.3. Reproducibility

A considerable variability in the responses was found over the three days, particularly with ChatGPT, raising concerns about the reliability of these tools for clinical use. Chatbots rely on probabilistic models to predict the most appropriate response, which may result in variability when the same question is asked multiple times, explaining the low reproducibility found in our results.

Poor reproducibility was also observed in a study where ChatGPT was asked to define a diet plan for a patient with T2DM. Repeated queries resulted in different and sometimes incorrect dietary plans, further highlighting that, at least at this stage, AI chatbots can only assist but not replace the expertise and critical judgement of nutrition professionals [14].

### 4.4. Consistency

Few inconsistencies within each response were observed in Case 1, but major and concerning inconsistencies were evident for Case 2. These inaccuracies were mostly related to recommendations relative to protein intake, a critical issue for patients with CKD and sarcopenia. When both conditions coexist, the clinician’s judgment is essential for determining nutritional management strategies. According to the ESPEN guidelines, physicians customarily assess the risks and benefits and use clinical judgment to make recommendations for older patients with CKD [27]. Several chatbots (ChatGPT 4.0, Chat Sonic, Perplexity AI, and Pi AI) demonstrated confusion by simultaneously recommending both reduced and increased protein intake, leading to a lack of clarity. Inconsistencies in managing multiple clinical scenarios by chatbots have been previously observed. ChatGPT 3.5 provided conflicting suggestions in a hypothetical case involving a patient with T2DM, obesity, and CKD [3] since recommendations for the three conditions were not integrated. For instance, although ChatGPT recommended prioritizing lean proteins to support muscle health, it also advised limiting overall protein intake [3]. These findings suggest that the attempt of chatbots to integrate various recommendations results in contradictory or inappropriate advice that could potentially confuse users.

### 4.5. Nutritional Adequacy of Diet Plans

Notable discrepancies were observed between the nutritional values provided by chatbots and those calculated by dietitians. Our findings were partially in line with the literature. Indeed, ChatGPT 3.5 exhibited a notable level of accuracy in estimating the energy content of 236 food items, with 66.4% of estimates falling within 10% of USDA values. However, its performance significantly declined for macronutrients, especially for lipids, with only 30% of estimates aligning with USDA values [9]. When ChatGPT 3.5 was used to create menus for patients with T2DM and metabolic syndrome, the dietary plans deviated from the target values (1500 kcal) up to 300 kcal [11]. In a comparison with ChatGPT and Bard in generating 2200 kcal meal plans, both chatbots failed to reach the recommended caloric targets by producing plans with lower energy content [31]. Conversely, a study evaluating the reliability of ChatGPT 3.5 and ChatGPT 4.0 in calculating the calorie and macronutrient content for eight menus designed for healthy adults revealed significant differences in protein estimates only, while the energy, carbohydrates, and fat content were consistent with those provided by the dietitians [32].

In conclusion, while the chatbots demonstrated a reasonable ability to estimate the energy content of a list of proposed foods, they exhibited significant inconsistencies in determining the nutritional composition of the meal plans which they themselves generated.

### 4.6. Future Perspective

The results of this study have highlighted the current limitations of chatbots and can guide future efforts to develop more reliable AI models for clinical nutrition by refining algorithms. Research involving larger sample sizes and more complex prompts should be conducted to optimize the use of AI tools in clinical nutrition. Future studies involving human subjects in real-world settings are also warranted to evaluate the effectiveness of AI-generated dietary plans.

Once refined, chatbots have the potential to become a valuable support for dietitians in their clinical practice by enhancing patient education, enabling effective meal planning, monitoring progress, analyzing nutritional data, and improving the overall efficiency and quality of clinical nutrition care. However, it is crucial for healthcare professionals to undergo training courses on the use of AI tools to fully understand their limitations and potential. Scientific societies should also play a key role by developing guidelines to standardize and optimize the use of AI in clinical nutrition. These steps are essential to ensure the effective and responsible adoption of AI technologies in healthcare

### 4.7. Strengths and Limitations

To the best of our knowledge, this is the first study to provide a comprehensive evaluation of a wide range of popular AI chatbots, offering valuable insights into their performance in generating dietary plans for individuals with obesity. The multiple assessments over three consecutive days enabled us to perform an evaluation of the reproducibility and consistency of chatbot responses.

Several limitations should be acknowledged. The small sample size, consisting of only two clinical cases and three consecutive requests per chatbot, restricts the potential for multivariate analysis. No formal sample size calculation was conducted, as this was an exploratory study. Future research should consider larger samples to improve the generalizability and robustness of the findings. This study used one prompt per each case, which may not fully capture the full range of the chatbots’ capabilities or the potential variability in responses to different types of prompts. Additionally, our analysis focused on the initial response generated by the chatbots while typical user interacts with multiple prompts for clarification. The dietary plans generated by the chatbots were often incomplete or insufficiently detailed, potentially leading to an underestimation of their nutritional value and limiting the evaluation of the nutritional consistency. The rapidly advancing nature of AI technology makes the results of our study ’transient’, as newer versions of these chatbots may already offer improved performance. Finally, the selected chatbots do not encompass all available AI tools, which may limit the generalizability of the findings. Moreover, this study focused on general-purpose freely available AI models, which were not specifically trained for healthcare applications, as their training prioritized general cognitive abilities [8]. Therefore, our results cannot be generalized to all chatbot types, particularly those specifically designed for medical applications.

## 5. Conclusions

AI chatbots were able to provide basic dietary advice for uncomplicated cases of obesity. However, their limited accuracy, reproducibility, and completeness in more complex clinical scenarios limit their applicability in clinical practice. The expertise of registered dietitians in delivering personalized advice and addressing the complexities and specific needs of each individual cannot currently be replaced by AI chatbots.

## Figures and Tables

**Table 1:** Characteristics of the chatbots.

Name	Developer	AI Model	Functionality	Availability
ChatGPT 3.5	OpenAI San Francisco, CA, USA	GPT 3.5	Text understanding and generation, language translation, code writing	Free
ChatGPT 4.0	OpenAI San Francisco, CA, USA	GPT 4.0	Text understanding and generation, language translation, code writing, and assistance with a wide range of text-based tasks	Limited free usage per day
Gemini	Google Mountain View, CA, USA	Not specified	Machine learning, natural language processing, text generation, question answering, Google search	Limited availability
Copilot	Microsoft Redmond, WA, USA	GPT 4 and Bing research	Answering questions, providing information, creating content, and performing online research to respond to specific requests	Free
Chatsonic	Writesonic San Francisco, CA, USA	GPT 4	Content generation, customer support, automation, and multi-language	Free
Perplexity AI	Perplexity San Francisco, CA, USA	Not specified	Language understanding, text generation, research and information synthesis, problem analysis and resolution, creativity, tutoring and support	Free
Claude	Anthropic San Francisco, CA, USA	Claude 3	Analysis, question answering, mathematics, programming, creative writing, teaching, and general discussions	Free
Pi AI	Inflection AI Palo Alto, CA, USA	Inflection-1	Providing personalized recommendations, helping with personal growth, offering information on various topics, assisting with everyday tasks, answering questions on complex subjects, offering emotional support, and engaging in casual conversations based on user interests	Free
You.com	SuSea, Inc. Palo Alto, CA, USA	Artificial Narrow Intelligence (ANI)	Answering questions, providing assistance, processing information, offering suggestions, interacting naturally, and updating	Free
ZenoChat	TextCortex AI Berlin, Germany	Sophos AI model	Conversational assistance, content creation, analysis and synthesis, research support, calculation and data analysis, planning and organization, creative support	Limited free usage

**Table 2:** Outcomes definition and Score System Criteria.

a. Case 1		
**Outcome**	**Question for the Experts**	**Score System**
**Accuracy** The ability of chatbots to deliver precise and accurate advice in line with international guidelines.	Does the chatbot suggest an appropriate caloric intake?	Each question was evaluated with a score ranging from 0 to 4 0 = totally disagree 1 = disagree 2 = neither agree or disagree 3 = agree 4 = totally agree
	Does the chatbot suggest an appropriate macronutrients distribution?	
	Does the chatbot’s response encourage the consumption of whole grains and foods rich in fiber?	
	Does the chatbot’s response advise limiting or avoiding sugars?	
	Does the chatbot’s response provide adequate recommendations regarding physical activity?	
	**Maximum score for accuracy**	**20**
**Completeness** Evaluates whether responses are comprehensive and include all relevant advice.	Does the chatbot provide a calorie intake target?	Each question was evaluated with a score ranging from 0 to 1. 0 = No 1 = Yes
	Does the chatbot provide advice on nutrient distribution?	
	Does the chatbot’s answer provide a diet plan?	
	Does the chatbot answer provide the portion size of food included in the diet plan?	
	Does the chatbot provide advice on dietary fiber?	
	Does the chatbot provide advice on sugars?	
	Does the chatbot provide advice on physical activity?	
	**Maximum score for completeness**	**7**
**Reproducibility** The ability of chatbots to generate similar answers across the three days with no fundamental differences or inconsistencies.	Do the answers provide (or not provide) the dietary plan?	Each question was evaluated with a score ranging from 0 to 2 0 = responses are inconsistent (i.e., they differ from each other) 1 = Two out of three responses are consistent 2 = All three responses are consistent
	Do the answers provide the same caloric target?	
	Do the answers provide the same macronutrient composition?	
	Do the answers provide the same advice on dietary fiber?	
	Do the answers provide the same advice on sugars?	
	Do the answers provide the same recommendations about physical activity?	
	**Maximum score for reproducibility**	**12**
**Consistency** Assesses the internal coherence of each response. Ensures advice is reliable and not confusing, maintaining consistency across various aspects of dietary therapy.	Does the chatbot’s response provide inconsistent advice? If yes, specify which inconsistencies and their severity.	Number of major inconsistencies? (The advices are completely contradictory) Number of minor inconsistencies? (The advices are partially contradictory)
**Nutritional adequacy** Evaluate whether the nutritional values of the diet plan provided by the chatbot correspond to those calculated by a dietitian.	Energy adequacy: Deviation < 200 calories between those declared by the chatbot and those calculated by a human dietitian Macronutrients adequacy: Deviation < 5% between the % of macronutrient (protein, carbohydrates, fats) declared by the chatbot and those calculated by a human dietitian	For each value: Yes: 1 (consistent) No: 0 (inconsistent) score: 0 to 4 0 = all 4 values (calories and the three macronutrients) show significant deviations 1 = 3 values show significant deviation 2 = 2 values show significant deviation 3 = 1 value shows a significant deviation 4 = no values show significant deviations
**b. Case 2**		
**Outcome**	**Question for the expert**	**Score system**
**Accuracy** The ability of chatbots to deliver precise and accurate advice in line with international guidelines.	Does the chatbot suggest an appropriate caloric intake?	Each question was evaluated with a score ranging from 0 to 4 0 = totally disagree 1 = disagree 2 = neither agree or disagree 3 = agree 4 = totally agree
	Does the chatbot suggest an appropriate protein intake?	
	Does the chatbot’s response provide correct advice on carbohydrates?	
	Does the chatbot’s response provide correct advice on dietary fiber?	
	Does the chatbot recommend an appropriate sodium intake?	
	Does the chatbot’s response provide correct advice on hydration?	
	Does the chatbot’s response provide correct advice on phosphorus?	
	Does the chatbot’s response provide adequate recommendations regarding physical activity?	
	**Maximum score for accuracy**	**32**
**Completeness** Evaluates whether responses are comprehensive and include all relevant advice.	Does the chatbot provide a calorie intake target?	Each question was evaluated with a score ranging from 0 to 1 0 = No 1 = Yes
	Does the chatbot provide advice on nutrient distribution?	
	Does the chatbot’s answer provide a diet plan?	
	Does the chatbot answer provide the portion size of food included in the diet plan?	
	Does the chatbot provide advice on fiber?	
	Does the chatbot provide advice on sugars?	
	Does the chatbot provide advice on sodium?	
	Does the chatbot provide advice on hydration?	
	Does the chatbot provide advice on phosphorus?	
	Does the chatbot provide advice on physical activity?	
	**Maximum score for completeness**	**10**
**Reproducibility** The ability of chatbots to generate similar answers across the three days with no fundamental differences or inconsistencies.	Do the answers provide (or not provide) a dietary plan?	Each question was evaluated with a score ranging from 0 to 2 0 = responses are inconsistent (i.e., they differ from each other) 1 = Two out of three responses are consistent 2 = All three responses are consistent
	Do the answers provide the same caloric target?	
	Do the answers provide the same macronutrient composition?	
	Do the answers provide the same advice on dietary fiber?	
	Do the answers provide the same advice on sugars?	
	Do the answers provide the same advice on sodium?	
	Do the answers provide the same advice on phosphorus?	
	Do the answers provide the same advice on hydration?	
	Do the answers provide the same advice about physical activity?	
	**Maximum score for reproducibility**	**18**
**Consistency** Assesses the internal coherence of each response. Ensures advice is reliable and not confusing, maintaining consistency across various aspects of dietary therapy.	Does the chatbot’s response provide inconsistent advice? If yes, specify which inconsistencies and their severity.	Number of major inconsistencies? (The advices are completely contradictory) Number of minor inconsistencies? (The advices are partially contradictory)
**Nutritional adequacy** Evaluate whether the nutritional values of the diet plan provided by the chatbot correspond to those calculated by a dietitian.	Energy adequacy: Deviation < 200 calories between those declared by the chatbot and those calculated by a human dietitian Macronutrients adequacy: Deviation < 5% between the % of macronutrient (protein, carbohydrates, fats) declared by the chatbot and those calculated by a human dietitian	For each value: Yes: 1 (consistent) No: 0 (inconsistent) score: 0 to 4 0 = all 4 values (calories and the three macronutrients) show significant deviations 1 = 3 values show significant deviation 2 = 2 values show significant deviation 3 = 1 value shows a significant deviation 4 = no values show significant deviations

**Table 3:** Mean and standard deviation score of accuracy of relevant topics for Case 1 (range 0–4).

	Does the Chatbot Suggest an Appropriate Caloric Intake?	Does the Chatbot Suggest an Appropriate Macronutrient Distribution?	Does the Chatbot’sResponse Encourage the Consumption of Whole Grains and Foods Rich in Fiber?	Does the Chatbot’s Response Advise Limiting or Avoiding Sugars?	Does the Chatbot’s Response Provides Adequate Recommendations Regarding Physical Activity?
ChatGPT 4.0	2.67 ± 0	3.11 ± 0.77	2.67 ± 0.33	0.78 ± 0.19	2.56 ± 0.20
ChatGPT 3.5	3.67 ± 0	2.78 ± 0.19	2.89 ± 0.19	1.89 ± 0.51	2.22 ± 0.51
Gemini	1.55 ± 0.67	-	2.78 ± 0.77	2.11 ± 0.84	1.56 ± 0.39
Copilot	1.0 ± 0.67	0.56 ± 0.20	1.56 ± 0.38	0.67 ± 0	0.44 ± 0.20
Chatsonic	2.22 ± 1.0	-	2.22 ± 0.77	-	1.78 ± 0.39
Perplexity AI	3.1 ± 0.51	1.22 ± 0.19	2.11 ± 0.19	0.78 ± 0.19	0.67 ± 0
Claude	2.56 ± 0.39	0.89 ± 0.51	2.67 ± 0.66	1.89 ± 0.96	2.11 ± 0.38
Pi AI	2.22 ± 0.51	-	2.11 ± 0.19	1.11 ± 0.38	3.11 ± 0.19
You.com	0.44 ± 0.20	1.56 ± 0.77	2.33 ± 0.58	0.89 ± 0.38	0.44 ± 0.20
ZenoChat	3.22 ± 0.51	3.33 ± 0.58	1.89 ± 0.84	-	1.22 ± 0.51

Scores not reported in case of missing data by the chatbots.

**Table 4:** Evaluation of consistency of Case 1.

		Inconsistency Description	
		Major	Minor
Perplexity AI	Day 2	The suggested caloric intake differs from the caloric content of the diet plan by 700 kcal	-
ZenoChat	Day 1	-	The suggested caloric intake differs from the caloric content of the diet plan by 300 kcal

**Table 5:** Evaluation of nutritional adequacy of Case 1.

		**Nutritional Values Declared by Chatbot**				**Nutritional Values Calculated by Dietitians According to Diet Plan Proposed by Chatbot**				Delta Energy (kcal)	Delta Proteins (%)	Delta Fats (%)	Delta CHOs (%)	Total Score
		Energy (kcal)	Protein (%)	Fats (%)	CHOs (%)	Energy (kcal)	Protein (%)	Fats (%)	CHOs (%)					
ChatGPT 4.0	D1	1936	20	30	50	2144	22	39	39	208 (10.7%)	2	9	−11	1
	D2	2148	20	30	50	2344	22	35	43	196 (9%)	2	5	−7	2
	D3	1691	25	30	45	1523	28	34	38	−168 (−9.9%)	3	4	−7	3
ChatGPT 3.5	D1	1800–2000	25–30	20–25	45–50	1807	25	42	33	0	0	17	−12	2
	D2	2000	-	-	-	1580	27	47	26	−420 (−21%)	-	-	-	-
	D3	2000	25–30	20–35	45–50	1870	25	44	31	−130 (−6.5%)	0	9	−14	2
Chatsonic	D1	1936	-	-	-	1687	31	30	39	−249 (12.9%)	-	-	-	-
	D2	1560	-	-	-	1506	30	36	34	−54 (−3.5%)	-	-	-	-
Perplexity AI	D1	1800	-	-	-	1561	29	26	45	−239 (−15.4%)	-	-	-	-
	D2	1500	-	-	-	1367	30	26	44	−133 (−8.9%)	-	-	-	-
	D3	1900	15–20	20–25	55–60	1572	23	25	52	−32 (17.3%)	3	0	−3	3
Claude	D1	2000–2400	25–30	25–30	40–45	1836	30	34	36	−164 (8.2%)	0	4	−4	4
	D2	1800–2250	-	-	-	1782	31	36	33	−18 (−1%)	-	-	-	-
	D3	2200–2650	-	-	-	1879	27	38	35	−321 (−14.6%)	-	-	-	-
Pi AI	D2	1800–2000	-	-	-	1679	25	38	37	−121 (−6.7%)	-	-	-	-
	D3	1950	-	-	-	1587	21	42	37	−363 (−18.6%)	-	-	-	-
ZenoChat	D1	1600–1800	20–35	20–25	45–55	1931	28	33	39	131 (7.2%)	0	8	−6	2
	D2	1800	20–35	20–30	45–50	1724	29	35	36	−76 (−4.2%)	0	5	−4	3
	D3	2100	25–30	20–30	40–50	1818	33	36	31	−282 (−13.4%)	3	6	−9	1

Scores not reported in case of missing data by the chatbots. CHOs = carbohydrates; Delta = difference between the nutritional values calculated by dietitians and those declared by chatbot.

**Table 6:** Mean score and standard deviation of accuracy of relevant topics for Case 2 (range 0–4).

	Does the Chatbot Suggest an Appropriate Caloric Intake?	Does the Chatbot Suggest an Appropriate Protein Intake?	Does the Chatbot’s Response Provide Correct Advice on Carbohydrates?	Does the Chatbot’s Response Provide Correct Advice on Fiber?	Does the Chatbot Recommend an Appropriate Sodium Intake?	Does the Chatbot’s Response Provide Correct Advice on Hydration?	Does the Chatbot’s Response Provide Correct Advice on Phosphorus?	Does the Chatbot’s Provide Adequate Recommendations Regarding Physical Activity?
ChatGPT 4.0	1 ± 0.58	2.11 ± 0.70	3.22 ± 0.39	2.67 ± 0.33	1.89 ± 0.38	1.89 ± 0.38	0.56 ± 0.20	2.44 ± 0.20
ChatGPT 3.5	1.33 ± 0.33	2.67 ± 0	3.0 ± 0.33	1.11 ± 0.38	-	2.33 ± 0	-	2.0 ± 0.33
Gemini	0.93 ± 0.30	3 ± 0.58	3.0 ± 0	1.67 ± 1.20	-	2.11 ± 0.38	-	2.44 ± 0.84
Copilot	-	2.56 ± 0.83	2.44 ± 0.69	2.67 ± 0.58	-	0.78 ± 0.39	-	2.33 ± 0.88
Chatsonic	0.89 ± 0.19	1 ± 0.33	1.89 ± 0.38	2 ± 0.67	3.11 ± 0.38	0.89 ± 0.19	1.44 ± 0.39	1.89 ± 0.38
Perplexity AI	2.33 ± 1.45	0.78 ± 0.69	2.22 ± 0.19	2.78 ± 0.19	-	1.22 ± 0.19	-	0.67 ± 0
Claude	2.44 ± 1.26	2.56 ± 0.96	1.67 ± 0.58	2.67 ± 0.58	1.44 ± 0.20	1.22 ± 0.39	-	0.78 ± 0.19
Pi AI	0.89 ± 0.53	0.33 ± 0	1.11 ± 0.70	1.56 ± 0.77	1 ± 0.33	0.89 ± 0.19	0.44 ± 0.20	1.0 ± 0
You.com	0.67 ± 0	1.56 ± 0.96	2.67 ± 0.58	2.33 ± 0.66	-	1.33 ± 0.58	-	0.33 ± 0
ZenoChat	2.44 ± 0.51	2.56 ± 0.51	2.56 ± 0.51	2.0 ± 1.15	0.56 ± 0.20	1.33 ± 0.58	-	0.56 ± 0.20

Scores not reported in case of missing data by the chatbots.

**Table 7:** Evaluation of consistency of Case 2.

		Inconsistency Description	
		Major	Minor
ChatGPT 4.0	Day 1	Conflicting advice on protein intake	The suggested caloric intake differs from the caloric content of the diet plan by 200 kcal
Chatsonic	Day 2	The diet plan includes margarine, even though the tips advise avoiding it	-
	Day 3	Conflicting advice on protein intake	It recommends limiting refined grains, yet subsequently includes white bread in the diet plan
Perplexity AI	Day 3	Incorrect protein calculation The diet plan provides a greater amount of protein compared to the suggested intake	-
Claude	Day 1	-	Inaccurate caloric intake calculation of the diet plan
Pi AI	Day 1	Conflicting advice on protein intake	-

**Table 8:** Evaluation of Nutritional adequacy of Case 2.

		Nutritional Values Declared by Chatbot				Nutritional Values Calculated by Dietitians According to Dietary Plan Proposed by Chatbot				Delta Energy (kcal)	Delta Proteins (%)	Delta Fats (%)	Delta CHOs (%)	Total Score
		Energy (kcal)	Protein (%)	Fats (%)	CHOs (%)	Energy (kcal)	Protein (%)	Fats (%)	CHOs (%)					
ChatGPT 4.0	D1	1049	20–25	25–30	45–50	1558	26	46	28	509 (48.5%)	1	16	−17	1
	D2	1680	30	28	42	1652	27	51	22	−28 (−1.7%)	−3	23	−20	2
	D3	1400–1600	20–25	25–30	45–50	1616	27	36	37	16 (1%)	2	6	−8	2
ChatGPT 3.5	D2	1200–1600	20	30	50	1323	31	32	37	0	1	2	−13	3
	D3	1200–1400	20–25	25–30	45–50	1417	24	48	28	17 (1.2%)	0	18	−17	2
Chatsonic	D2	1200–1600	-	-	-	1728	20	29	51	128 (8%)	-	-	-	-
Perplexity AI	D1	1500–1650	16–22	24–33	32–39	1967	25	31	44	317 (16.1%)	3	2	5	2
	D2	1500	-	-	44	1494	33	31	36	−6 (−0.4%)	-	-	8	-
	D3	1800	25	30	45	1475	29	37	34	−325 (18%)	4	7	−11	1
Claude	D1	1530	20–25	30–35	40–45	1546	25	39	36	16 (1%)	0	4	−4	4
	D2	1600–1900	20–25	30–35	40–45	1570	25	42	33	−30 (1.9%)	0	7	−7	2
	D3	1600–1900	20–25	30–35	40–45	1527	26	41	33	−73 (4.6%)	1	6	−7	2
Pi AI	D2	1200–1300	-	-	-	1053	26	35	39	−147 (12.2%)	-	-	-	-
	D3	1400–1600	-	-	-	1527	22	24	54	0	-	-	-	-
ZenoChat	D1	1400–1650	20–25	30–35	40–45	2550	24	34	42	900 (55%)	0	0	0	3
	D2	1550–1800	20–25	25–30	45–55	2239	25	40	35	439 (24.4%)	0	10	−10	1
	D3	1500	-	-	-	1744	28	37	35	244 (16.3%)	-	-	-	-

Scores not reported in case of missing data by the chatbots. CHOs = carbohydrates; Delta = difference between the nutritional values calculated by dietitians and those declared by chatbot.

## Data Availability

Data are contained within the article and Supplementary Materials.

## Supplementary Materials

The following supporting information can be downloaded at: https://www.mdpi.com/article/10.3390/jcm13247810/s1, Table S1: Outcomes definition and score system criteria for Case 1 (a) and Case 2 (b); Table S2: Chatbot responses for Case 1 and Case 2; Table S3: Evaluation of the accuracy, completeness, and reproducibility of Case 1 (a) and Case 2 (b).
