# Supplementary material for: Comparison of the Accuracy, Completeness, Reproducibility, and Consistency of Different AI Chatbots in Providing Nutritional Advice: An Exploratory Study
Source: J Clin Med. 2024 Dec 20;13(24):7810. doi: 10.3390/jcm13247810 (PMC11677083; doi:10.3390/jcm13247810)
Supplement: Supplementary file 1 [file jcm-13-07810-s001.zip › Supplementary Table S1.pdf]

Table S1

a. Recommendations from guidelines used for experts' evaluation of Case 1

**Question for the experts**	**Recommendations from guidelines**
Does the chatbot suggest an **appropriate caloric intake?**	• Calorie restriction should be individualized and take account of nutritional habits, physical activity, co-morbidities, and previous dieting attempts [13].<br>• A 135–30% decrease in energy (calorie) intake from habitual intake in a weight-stable individual is sufficient and appropriate [13].<br>• An easy rule of thumb is a daily energy requirement of 25 kcal/kg [13].<br>• The recommended weight-reducing dietary regimen tailored to an individual's need usually provides an energy deficit of 600 kcal/day [13].<br>• Reduce energy intake by 500-1000 kcal/day [13].
Does the chatbot suggest an **appropriate macronutrients distribution**?	• Balanced hypocaloric diets result in clinically meaningful weight loss regardless of which macronutrients they emphasize. An emphasis put on the macronutrient proportion in the various diets (low fat, low carbohydrate or high protein, etc.) has not proved better than a balanced hypocaloric diet, except for low-glycaemic load diets (carbohydrate content of the diet × glycaemic index) in the short term [13].<br>• Balanced hypocaloric diets can be tailored to individual patients on the basis of their personal and cultural preferences and may therefore have the best chance for long-term success (e.g. Mediterranean diet) [13].<br>• The dietary reference intakes permit wide acceptable macronutrient distribution ranges. They allow, for example, 45% to 65% of calories from carbohydrate, 10% to 35% of calories from protein and 20% to 35% of calories from fat (with 5% to 10% of calories derived from linoleic acid and 0.6% to 1.2% of calories derived from alpha linolenic acid) [14].<br>• Adults living with obesity can consider any of the multiple medical nutrition therapies to improve health-related outcomes, choosing the dietary patterns and/or food-based approaches that support their best long-term adherence [14].<br>• Calorie-restricted dietary patterns emphasizing variable macronutrient distribution ranges (lower, moderate, or higher carbohydrate with variable proportions of protein and fat) to achieve similar body weight reduction over 6-12 months [14].
Does the chatbot's response encourage the consumption of **whole grains and foods rich in fiber**?	• Dietary advice should encourage healthy eating and emphasise the need to increase consumption of vegetables, beans, legumes, lentils, grain, unsweetened cereals and fibre, and to substitute low-fat dairy products and meats for high-fat alternatives [13].<br>• Pulses, vegetables, fruit, whole grains, dairy foods are recommended [14].<br>• Whole grains (especially from oats and barley) to improve established lipid targets, including total cholesterol and LDL-C [14].<br>• Pulses (i.e. beans, peas, chickpeas, lentils) to improve body weight improve glycemic control, established lipid targets, including LDL-C, systolic BP and reduce the risk of coronary heart disease [14].

	• Vegetables and fruit to improve diastolic, glycemic control, reduce the risk of type 2 diabetes and cardiovascular mortality [14].
Does the chatbot's response advise limiting or **avoiding sugars?**	• It is recommended to avoid foods containing added sugars and solid fats, as well as consumption of sugary drinks and alcohol-containing beverages [13].<br>• Limit foods high in sodium, sugars or saturated fat [14].
Does the chatbot's response provide adequate recommendations regarding **physical activity**?	• Aerobic training is the optimal mode of exercise for reducing fat mass and body mass while a programme including resistance training is needed for increasing lean mass in middle-aged and overweight/obese individuals [13]<br>• At least 30 min/week of moderate aerobic exercise (such as brisk walking) should be combined with three weekly sessions of resistance exercise to increase muscle strength [13]<br>• Aerobic physical activity (30–60 minutes of moderate to vigorous intensity most days of the week) can be considered for adults who want to Achieve small amounts of body weight and fat loss; Achieve reductions in abdominal visceral fat and ectopic fat such as liver and heart fat, even in the absence of weight loss; Favour weight maintenance after weight loss; Favour the maintenance of fat-free mass during weight loss; and Increase cardiorespiratory fitness and mobility [14].<br>• For adults living with overweight or obesity, resistance training may promote weight maintenance or modest increases in muscle mass or fat-free mass and mobility [14].

b. Recommendations from guidelines used for experts' evaluation of Case 2

**Question for the expert**	**Recommendations from guidelines**
Does the chatbot suggest an **appropriate caloric intake**?	CKD:<br>• In adults with CKD 1-5D (1C) or post-transplantation who are metabolically stable, we recommend prescribing an energy intake of 25-35 kcal/kg body weight per day based on age, sex, level of physical activity, body composition, weight status goals, CKD stage, and concurrent illness or presence of inflammation to maintain normal nutritional status [16]<br>T2DM:<br>• Interventions including high frequency of counseling (≥16 sessions in 6 months) with focus on nutrition changes, physical activity, and behavioral strategies to achieve a 500–750 kcal/day energy deficit have been shown to be beneficial for weight loss and should be considered when available [17]
Does the chatbot suggest an **appropriate protein intake**?	CKD:<br>• Maintaining a protein intake of 0.8 g protein/kg (weight)/d for those with diabetes and CKD not treated with dialysis [15].

	• The KDIGO guideline suggested more liberalization with protein restriction and recommended that 0.8 g/kg body weight per day be maintained, avoiding levels > 1.3 g/kg body weight [16].<br>• For people with non–dialysis-dependent stage G3 or higher CKD, dietary protein intake should be aimed to a target level of 0.8 g/kg body weight per day [17].<br>SARCOPENIA:<br>• For healthy older adults, we recommend a diet that includes at least 1.0e1.2 g protein/kg body weight/day. For certain older adults who have acute or chronic illnesses, 1.2e1.5 g protein/kg body weight/day may be indicated, with even higher intake for individuals with severe illness or injury [18].<br>• In older patients with moderately impaired GFR or another form of CKD, physicians customarily assess the balance between risks and benefits, and use clinical judgment to make recommendations. In patients with severe CKD, it is usual to recommend a lower protein intake of 0.6-0.8 g/kg/day with sufficient energy intake (about 30 kcal/kg/day). However, multi-morbid patients on palliative care are an exception to this rule. These individuals may benefit from unrestricted intake of dietary protein because the short-term risks associated with protein energy wasting are greater than the long-term risks of worsening renal function due to diet with standard protein content [18].
Does the chatbot's response provide correct advice on **carbohydrates**?	• Consider reducing overall carbohydrate intake for adults with diabetes to improve glycemia, as this approach may be applied to a variety of eating patterns that meet individual needs and preference [17].<br>• People with diabetes and those at risk are advised to replace sugar-sweetened beverages (including fruit juices) with water or low-calorie or no-calorie beverages [17].
Does the chatbot's response provide correct advice on dietary **fiber**?	Emphasize minimally processed, nutrient-dense, high-fiber sources of carbohydrate (at least 14 g fiber per 1,000 kcal) [17].
Does the chatbot recommend an appropriate **sodium intake**?	• Sodium intake <2 g be of sodium per day in patients with diabetes and CKD [15].<br>• As for the general population, people with diabetes are advised to limit their sodium consumption to <2300 mg/day [17].
Does the chatbot's response provide correct advice on **hydration**?	
Does the chatbot's response provide correct advice on **phosphorus**?	• In adults with CKD 3-5D, we recommend adjusting dietary phosphorus intake to maintain serum phosphate levels in the normal range [16].<br>• How much dietary phosphorus/phosphate should be restricted in adult patients with CKD is not well established. Traditionally, CKD-specific recommendations suggest maintaining phosphorus intake between 800 and 1,000 mg/d in patients with CKD stages 3-5 and those receiving maintenance dialysis to maintain serum phosphate levels in the normal range. However, the work group notes that the efficacy of this recommendation has not been established. Further, such a dietary phosphorus intake range is higher than the current recommended dietary allowance for phosphorus in the adult general population (700 mg/d) [16].

	• Limiting dietary phosphorus intake (per se or in combination with restriction of dietary protein, the major source of dietary phosphorus) may be recommended to prevent/treat complications related to high phosphate load in patients with CKD stages 3-5 and maintenance dialysis [16].
Does the chatbot's response provide adequate recommendations regarding **physical activity**?	• We recommend that patients with diabetes and CKD be advised to undertake moderate-intensity physical activity for a cumulative duration of at least 150 minutes per week, or to a level compatible with their cardiovascular and physical tolerance [16].<br>• 60 min/day or more of moderate- or vigorous-intensity aerobic activity, with vigorous muscle-strengthening and bone-strengthening activities at least 3 days/week [17].<br>• 150 min or more of moderate- to vigorous-intensity aerobic activity per week, spread over at least 3 days/ week, with no more than 2 consecutive days without activity [17].<br>• In patients with sarcopenia, prescription of resistance-based training may be effective to improve lean mass, strength and physical function [19].<br>• In patients with sarcopenia, prescription of resistance-based training may be effective to improve lean mass, strength and physical function [18].
