# Supplementary material for: Comparison of the Accuracy, Completeness, Reproducibility, and Consistency of Different AI Chatbots in Providing Nutritional Advice: An Exploratory Study
Source: J Clin Med. 2024 Dec 20;13(24):7810. doi: 10.3390/jcm13247810 (PMC11677083; doi:10.3390/jcm13247810)
Supplement: Supplementary file 1 [file jcm-13-07810-s001.zip › Supplementary Table S2.pdf]

ChatGPT 4.0 case 1		
June 27th	June 28th	June 29th
Certainly! To create a tailored diet plan, let's start with some basic calculations and guidelines. For weight loss, it's important to create a calorie deficit while ensuring you get the nutrients you need. Here are the steps:<br><br>Calculate Basal Metabolic Rate (BMR):<br>Your BMR is the number of calories your body needs at rest to maintain basic physiological functions. We can use the Mifflin-St Jeor equation for this:<br>BMR=10×weight (kg)+6.25×height (cm)−5×age (years)+5<br>For you:<br>BMR=10×110+6.25×176−5×35+5<br><br>BMR=1100+1100−175+5<br><br>BMR=2030 calories/day<br><br>Calculate Total Daily Energy Expenditure (TDEE):<br><br>Since you have a sedentary lifestyle, we'll multiply your BMR by 1.2 (sedentary activity level):<br>TDEE=BMR×1.2=2030×1.2=2436 calories/day<br><br>Caloric Deficit for Weight Loss:<br>To lose weight, a common recommendation is a deficit of 500-1000 calories per day, aiming for a weight loss of about 0.5-1 kg per week.<br>Let's start with a 500-calorie deficit:<br>Caloric intake = TDEE - 500 = 2436 - 500 = 1936 calories/day<br><br>Macronutrient Distribution:<br>A balanced approach is:<br>45-55% carbohydrates<br>25-35% fats<br>15-25% proteins<br><br>For simplicity, we can use:<br>50% carbohydrates<br>30% fats<br>20% proteins<br><br>This translates to:<br>Carbohydrates: 1936 \times 0.5 = 968 calories (about 242 grams)	Certainly! Crafting a diet plan that suits your specific needs involves considering your age, height, weight, activity level, and nutritional goals. Based on your details, here's a tailored plan:<br><br>Step 1: Calculate Your Caloric Needs<br>Given your sedentary lifestyle, we can use the Harris-Benedict Equation to estimate your daily calorie needs:<br><br>Basal Metabolic Rate (BMR):<br>BMR=88.362+(13.397×weight in kg)+(4.799×height in cm)−(5.677×age in years)<br><br>Daily Caloric Needs (for a sedentary lifestyle):<br><br>Calories=BMR×1.2<br><br>Let's calculate:<br>BMR=88.362+(13.397×110)+(4.799×176)−(5.677×35)<br><br>BMR=88.362+1473.67+843.424−198.695=2206.761<br><br>Daily Caloric Needs=2206.761×1.2=2648.113<br><br>Step 2: Set a Caloric Deficit<br>To lose weight safely, a deficit of 500 calories per day is often recommended:<br><br>Target Calories per Day=2648.113−500=2148.113<br><br>Step 3: Macronutrient Distribution<br>A balanced macronutrient distribution could be:<br><br>Carbohydrates: 45-55% of total calories<br>Proteins: 25-35% of total calories<br>Fats: 20-30% of total calories<br>For simplicity, let's go with:<br><br>50% Carbohydrates<br>30% Proteins<br>20% Fats<br>Step 4: Calculate Macronutrient Needs<br>Using your target of 2148 calories:	Creating a diet plan tailored to your specific characteristics involves considering your current weight, height, age, and activity level. Given your sedentary lifestyle, the primary goal would be to create a calorie deficit to promote weight loss while ensuring balanced nutrition. Here's a suggested diet plan with portion sizes:<br><br>Daily Caloric Needs<br>Based on your information:<br><br>Age: 35 years<br>Height: 176 cm (5'9")<br>Weight: 110 kg (242 lbs)<br>Activity Level: Sedentary<br>Your Basal Metabolic Rate (BMR) and Total Daily Energy Expenditure (TDEE) can be estimated using the Mifflin-St Jeor Equation.<br><br>Calculating BMR and TDEE<br>BMR Calculation:<br><br>BMR=10×weight in kg+6.25×height in cm−5×age in years+5<br>BMR=10×110+6.25×176−5×35+5=1826 kcal/day<br>TDEE Calculation (for a sedentary lifestyle, multiply BMR by 1.2):<br>TDEE=1826×1.2=2191 kcal/day<br>To lose weight, a common recommendation is to reduce caloric intake by 500 kcal per day, aiming for about 1 pound (0.45 kg) of weight loss per week.<br><br>Target Daily Calories: 1691 kcal/day (2191 - 500)<br>Suggested Diet Plan<br><br>Macronutrient Distribution<br><br>Protein: 25% of daily calories<br>Fat: 30% of daily calories<br>Carbohydrates: 45% of daily calories<br><br>Meal Plan<br>Breakfast<br><br>Calories: ~400 kcal<br>Portion Sizes:<br>2 boiled eggs (140 kcal)

Fats: 1936 \times 0.3 = 580 calories (about 64 grams)
Proteins: 1936 \times 0.2 = 387 calories (about 97 grams)

Meal Planning:
Let's divide these calories and macros into meals. Here's a sample daily diet plan:

Breakfast (400-450 calories)
Oatmeal: 1 cup cooked (154 calories)
Greek Yogurt: 1 cup (100 calories)
Blueberries: 1/2 cup (42 calories)
Almonds: 1 oz (164 calories)

Mid-Morning Snack (150-200 calories)
Apple: 1 medium (95 calories)
Peanut Butter: 1 tbsp (95 calories)

Lunch (500-550 calories)
Grilled Chicken Breast: 150g (165 calories)
Quinoa: 1 cup cooked (222 calories)
Steamed Broccoli: 1 cup (55 calories)
Olive Oil: 1 tbsp (119 calories)
Afternoon Snack (150-200 calories)
Carrot Sticks: 1 cup (50 calories)
Hummus: 4 tbsp (100 calories)

Dinner (500-550 calories)
Salmon: 150g (280 calories)
Brown Rice: 1/2 cup cooked (108 calories)
Mixed Vegetables: 1 cup (50 calories)
Olive Oil: 1 tbsp (119 calories)
Evening Snack (100-150 calories)
Mixed Berries: 1 cup (84 calories)
Low-fat Cottage Cheese: 1/2 cup (80 calories)

Daily Totals
Calories: Approximately 1936
Carbohydrates: 240-250 grams
Proteins: 90-100 grams
Fats: 60-70 grams

Tips for Success
Hydration: Drink plenty of water throughout the day.
Physical Activity: Incorporate light physical activity, like walking, to

Carbohydrates:
Carbs (grams)=(0.50x2148)/4=268.5g

Proteins:
Proteins (grams)=(0.30x2148)/4=161.1g

Fats:
Fats (grams)=(0.20*2148)/9=47.7g

Step 5: Example Diet Plan
Here's a daily meal plan tailored to your needs:

Breakfast (~500 Calories)

Oatmeal: 1 cup cooked (154 calories)
Blueberries: ½ cup (42 calories)
Almonds: 1 ounce (28 grams) (164 calories)
Greek Yogurt: ½ cup (84 calories)

Nutritional Breakdown:
Carbs: ~64g
Proteins: ~17g
Fats: ~19g

Mid-Morning Snack (~200 Calories)
Apple: 1 medium (95 calories)
Peanut Butter: 1 tablespoon (94 calories)

Nutritional Breakdown:
Carbs: ~27g
Proteins: ~4g
Fats: ~8g

Lunch (~600 Calories)
Grilled Chicken Breast: 150 grams (165 calories)
Quinoa: 1 cup cooked (222 calories)
Mixed Vegetables: 1 cup steamed (120 calories)
Olive Oil: 1 tablespoon for dressing (119 calories)

Nutritional Breakdown:
Carbs: ~57g
Proteins: ~40g
Fats: ~25g

1 slice of whole-grain toast (70 kcal)
1 medium-sized apple (95 kcal)
1 cup of black coffee or tea (0 kcal)
1 tablespoon of peanut butter (95 kcal)

Mid-Morning Snack
Calories: ~150 kcal
Portion Sizes:
1 medium-sized banana (105 kcal)
10 almonds (45 kcal)

Lunch
Calories: ~450 kcal
Portion Sizes:
Grilled chicken breast (150g) (250 kcal)
Mixed salad (lettuce, tomatoes, cucumber, carrots) with 1 tablespoon of olive oil and vinegar dressing (100 kcal)
1 small whole-grain roll (100 kcal)

Afternoon Snack
Calories: ~150 kcal
Portion Sizes:
1 cup of low-fat yogurt (100 kcal)
1/2 cup of berries (50 kcal)

Dinner
Calories: ~450 kcal
Portion Sizes:
Baked salmon (150g) (280 kcal)
Steamed broccoli (1 cup) (55 kcal)
Quinoa (1/2 cup cooked) (115 kcal)

Evening Snack
Calories: ~90 kcal
Portion Sizes:
1 medium-sized orange (80 kcal)
Herbal tea (0 kcal)

Nutritional Guidelines
Proteins: Focus on lean proteins like chicken, turkey, fish, beans, and legumes.
Carbohydrates: Prefer complex carbs such as whole grains, vegetables, and fruits.
Fats: Include healthy fats from sources like avocados, nuts, seeds, and

enhance weight loss and improve overall health.
Consistency: Stick to the plan as much as possible, but allow for flexibility and occasional treats to maintain motivation.
This plan provides a balanced approach to weight loss, ensuring you get a variety of nutrients while maintaining a caloric deficit. Always consider consulting a healthcare professional or a dietitian before starting any new diet plan.

Afternoon Snack (~200 Calories)
Carrot Sticks: 1 cup (50 calories)
Hummus: 4 tablespoons (150 calories)

Nutritional Breakdown:
Carbs: ~25g
Proteins: ~6g
Fats: ~10g

Dinner (~600 Calories)
Salmon: 150 grams (280 calories)
Brown Rice: 1 cup cooked (216 calories)
Broccoli: 1 cup steamed (55 calories)
Olive Oil: 1 teaspoon for cooking (45 calories)

Nutritional Breakdown:
Carbs: ~52g
Proteins: ~35g
Fats: ~22g

Evening Snack (~150 Calories)
Mixed Berries: 1 cup (70 calories)
Dark Chocolate: 1 square (80 calories)

Nutritional Breakdown:
Carbs: ~25g
Proteins: ~2g
Fats: ~9g

Summary of Daily Intake
Calories: ~2250
Carbohydrates: ~250g
Proteins: ~104g
Fats: ~93g

Portion Size Guidelines
Proteins: Palm-sized portions (approx. 100-150g per serving)
Carbohydrates: Fist-sized portions (approx. 1 cup per serving)
Fats: Thumb-sized portions (approx. 1-2 tablespoons per serving)
Vegetables: Two fist-sized portions (approx. 2 cups per meal)

Hydration
Aim for 8-10 glasses (2-2.5 liters) of water daily.

olive oil.
Hydration: Drink plenty of water throughout the day.
Portion Control: Use measuring cups and a food scale to ensure accurate portion sizes.
Exercise: Incorporate at least 30 minutes of moderate exercise most days of the week to enhance weight loss and improve overall health.

Monitoring and Adjustments
Track your food intake and physical activity using a food diary or an app.
Adjust portion sizes and food choices based on your progress and nutritional needs.
Consult with a healthcare provider or a registered dietitian for personalized advice and to ensure the diet plan meets all your nutritional requirements.
Implementing this plan with consistency and commitment should help you achieve your weight loss goals while maintaining good nutrition.

	Tips for Success<br>Plan Meals: Prep and portion out your meals to avoid overeating.<br>Mindful Eating: Eat slowly and listen to your body's hunger signals.<br>Limit Sugars and Processed Foods: Focus on whole foods.<br>Stay Active: Incorporate light physical activity, like walking, to complement your diet.<br><br>Important Note<br>Before starting any new diet, it's advisable to consult with a healthcare provider or a dietitian to ensure it aligns with your health needs and goals.	

ChatGPT 4.0 case 2		
June 27th	June 28th	June 29th
Creating a diet plan tailored to your specific needs involves balancing various factors such as your type 2 diabetes, kidney function (as indicated by your creatinine level), weight management, and muscle mass improvement. Here's a structured plan:<br><br>Nutritional Goals:<br>Control Blood Sugar Levels: Focus on low glycemic index (GI) foods.<br>Support Kidney Health: Limit protein to moderate amounts and manage potassium and phosphorus intake.<br>Weight Management: Create a calorie deficit to promote weight loss.<br>Increase Muscle Mass: Incorporate adequate protein and strength-training exercises.<br><br>Caloric Intake:<br>For weight loss, a daily intake of about 1,200 to 1,500 calories is generally recommended for women. Given your sedentary lifestyle, starting with 1,200 calories might be appropriate, but you should consult your doctor for personalized advice.<br><br>Macronutrient Distribution:<br>Carbohydrates: 45-50% of total calories<br>Protein: 20-25% of total calories<br>Fats: 25-30% of total calories<br>Daily Caloric Breakdown:<br>Carbohydrates: 135-150 grams<br>Protein: 60-75 grams<br>Fats: 33-40 grams<br><br>Sample Meal Plan:<br>Breakfast:<br>Oatmeal: ½ cup cooked oats (75 calories), topped with ¼ cup berries (20 calories) and 1 tablespoon chia seeds (60 calories)<br>Greek Yogurt: ½ cup plain, non-fat Greek yogurt (60 calories)<br>Total: 215 calories<br><br>Mid-Morning Snack:<br>Apple: 1 medium apple (95 calories)<br>Total: 95 calories<br><br>Lunch:<br>Grilled Chicken Salad:<br>Grilled chicken breast: 3 ounces (130 calories)	Given your medical conditions and specific health considerations, it's crucial to create a diet plan that addresses your type 2 diabetes, reduced muscle mass, and overall weight management while being mindful of your kidney health due to your elevated creatinine level. Here's a personalized diet plan that focuses on balanced nutrition, controlled carbohydrate intake, and adequate protein, while supporting muscle mass and not overloading the kidneys.<br><br>Key Dietary Considerations:<br>Carbohydrate Management: Moderate carbohydrate intake to manage blood glucose levels.<br>Protein Intake: Adequate but not excessive protein to support muscle mass without burdening kidney function.<br>Healthy Fats: Include healthy fats to provide satiety and support cardiovascular health.<br>Calorie Control: Moderate caloric intake to promote weight loss in a healthy way.<br>Portion Sizes: Carefully managed portions to ensure balanced meals and blood sugar stability.<br><br>Daily Caloric Intake:<br>Given your weight, height, age, and sedentary lifestyle, a daily intake of about 1400-1600 calories is appropriate. This range supports gradual weight loss and muscle maintenance.<br><br>Macronutrient Distribution:<br>Carbohydrates: 40-45% of total calories (140-180 grams per day)<br>Protein: 25-30% of total calories (88-120 grams per day)<br>Fats: 25-30% of total calories (39-53 grams per day)<br><br>Meal Plan Outline:<br>Breakfast:<br>Option 1: Greek yogurt parfait<br>1 cup low-fat Greek yogurt (150g)<br>1/2 cup fresh berries (75g)<br>1 tablespoon chia seeds (12g)<br>1/4 cup rolled oats (20g)<br>Drizzle of honey (1 tsp, 5g)<br>Calories: ~350, Carbs: ~45g, Protein: ~20g, Fat: ~9g<br><br>Option 2: Vegetable omelette with whole-grain toast<br>2 eggs or 1/2 cup egg whites	Creating a personalized diet plan for you requires considering your specific health conditions and nutritional needs. Given your age, weight, height, sedentary lifestyle, type 2 diabetes, creatinine level, and reduced muscle mass, the plan should focus on managing blood sugar levels, supporting kidney health, and promoting muscle maintenance and overall well-being.<br><br>General Dietary Recommendations<br>Caloric Intake: Aiming for a calorie intake that supports gradual weight loss could be beneficial. For your specifics, a range of 1400-1600 calories per day might be appropriate, but consult with your healthcare provider or a dietitian for personalized advice.<br><br>Macronutrient Distribution:<br><br>Carbohydrates: 45-50% of total calories, focusing on complex carbohydrates with a low glycemic index.<br>Proteins: 20-25% of total calories, from high-quality, lean protein sources.<br>Fats: 25-30% of total calories, with an emphasis on healthy fats.<br>Fiber: Aim for at least 25-30 grams of fiber per day to help manage blood sugar levels.<br><br>Hydration: Drink plenty of water, but monitor fluid intake as recommended by your healthcare provider, especially considering your kidney function.<br><br>Sample Meal Plan<br>Breakfast<br>1 serving of oatmeal (1/2 cup of dry oats) topped with:<br>1/2 cup of fresh berries (e.g., blueberries or strawberries)<br>1 tablespoon of chia seeds or ground flaxseeds<br>1 boiled egg for additional protein<br><br>Morning Snack<br>1 small apple or a handful of nuts (about 10-12 almonds)<br><br>Lunch<br>Grilled chicken salad:<br>3-4 oz (about 85-115 grams) of grilled chicken breast<br>Mixed greens (spinach, kale, arugula) with:<br>1/2 cup of cherry tomatoes

Mixed greens: 2 cups (10 calories)<br>Cherry tomatoes: ½ cup (15 calories)<br>Cucumber: ½ cup (8 calories)<br>Olive oil & vinegar dressing: 1 tablespoon (80 calories)<br>Total: 243 calories<br><br>Afternoon Snack:<br>Almonds: 10 almonds (70 calories)<br>Total: 70 calories<br><br>Dinner:<br>Baked Salmon: 3 ounces (150 calories)<br>Steamed Broccoli: 1 cup (55 calories)<br>Quinoa: ½ cup cooked (111 calories)<br>Total: 316 calories<br><br>Evening Snack:<br>Carrot Sticks: 1 cup (50 calories)<br>Hummus: 2 tablespoons (60 calories)<br>Total: 110 calories<br>Daily Total: 1,049 calories (This is slightly low; you can adjust by adding a bit more protein or healthy fats to meet the 1,200-calorie goal.)<br><br>Key Points:<br>Low GI Foods: Focus on whole grains, legumes, non-starchy vegetables, and low-sugar fruits.<br>Protein: Opt for lean sources like poultry, fish, legumes, and low-fat dairy.<br>Fats: Include healthy fats from sources like olive oil, nuts, and avocados.<br>Portion Control: Use measuring cups and food scales to ensure accurate portion sizes.<br>Hydration: Drink plenty of water throughout the day.<br>Limit Sodium and Phosphorus: Avoid processed foods, limit dairy, and choose low-sodium options to support kidney health.<br>Physical Activity: Incorporate light strength-training exercises to help increase muscle mass, such as resistance band exercises or light weightlifting, under the guidance of a healthcare professional.<br><br>Regular Monitoring:<br>- Blood Sugar Levels: Monitor regularly to adjust your diet and medications as needed.<br>- Kidney Function: Regular check-ups to monitor creatinine and other kidney function parameters.<br>- Weight and Muscle Mass: Track progress and adjust dietary intake and	1/2 cup mixed vegetables (bell peppers, spinach, tomatoes) (75g)<br>1 slice whole-grain toast (30g)<br>1 small avocado (50g)<br>Calories: ~400, Carbs: ~25g, Protein: ~20g, Fat: ~24g<br><br>Mid-Morning Snack:<br>1 medium apple (150g) with 1 tablespoon peanut butter (16g)<br>Calories: ~200, Carbs: ~30g, Protein: ~4g, Fat: ~8g<br><br>Lunch:<br>Option 1: Grilled chicken salad<br>100g grilled chicken breast<br>Mixed greens (lettuce, spinach) (150g)<br>1/2 cup cherry tomatoes (75g)<br>1/4 cup shredded carrots (30g)<br>1/4 avocado (25g)<br>2 tablespoons olive oil and vinegar dressing (30g)<br>Calories: ~350, Carbs: ~15g, Protein: ~30g, Fat: ~20g<br><br>Option 2: Quinoa and black bean bowl<br>1/2 cup cooked quinoa (90g)<br>1/2 cup black beans (90g)<br>1/4 cup corn (30g)<br>1/2 cup diced bell peppers (75g)<br>1/4 avocado (25g)<br>Lime juice and cilantro for seasoning<br>Calories: ~400, Carbs: ~60g, Protein: ~15g, Fat: ~12g<br><br>Afternoon Snack:<br>1 small handful of almonds (28g)<br>Calories: ~170, Carbs: ~6g, Protein: ~6g, Fat: ~15g<br><br>Dinner:<br>Option 1: Baked salmon with steamed vegetables<br>120g baked salmon<br>1 cup steamed broccoli (150g)<br>1/2 cup roasted sweet potatoes (75g)<br>Calories: ~400, Carbs: ~35g, Protein: ~30g, Fat: ~18g<br><br>Option 2: Lentil soup with side salad<br>1 cup lentil soup (240g)<br>1 slice whole-grain bread (30g)<br>Side salad with mixed greens, 1 tbsp olive oil, and vinegar dressing (150g)	1/4 cup of cucumber slices<br>1/4 cup of shredded carrots<br>1 tablespoon of olive oil and vinegar dressing<br>1 small whole grain roll or 1/2 cup of quinoa<br><br>Afternoon Snack<br>1 cup of Greek yogurt (unsweetened) with:<br>1 tablespoon of sunflower seeds or pumpkin seeds<br><br>Dinner<br>Baked salmon (4 oz or 115 grams) with:<br>1/2 cup of steamed broccoli<br>1/2 cup of brown rice or a small sweet potato<br>1 cup of mixed green salad with a light dressing<br><br>Evening Snack<br>1 small pear or 1/2 cup of cottage cheese<br><br>Additional Tips<br>Monitor Blood Sugar: Keep track of your blood sugar levels to understand how different foods affect you.<br>Portion Control: Use measuring cups or a food scale to ensure portion sizes are appropriate.<br>Healthy Cooking Methods: Opt for grilling, baking, steaming, or sautéing with minimal oil.<br>Limit Sodium: Be mindful of sodium intake, especially with reduced kidney function.<br>Consult a Professional: Regularly check in with your healthcare provider or a registered dietitian to adjust the diet as needed based on your progress and any changes in your health status.<br>Physical Activity<br>While you mentioned having a sedentary lifestyle, incorporating light physical activities, such as walking or gentle strength exercises, can help improve muscle mass and overall health. Start with low-intensity activities and gradually increase as tolerated.<br><br>By focusing on a balanced diet that meets your specific health needs, you can manage your diabetes, support kidney function, and improve muscle mass over time.

exercise accordingly.<br><br>Always consult your healthcare provider or a registered dietitian before starting any new diet or exercise program, especially given your medical conditions. They can provide personalized recommendations and adjustments based on your overall health and medication regimen.	Calories: ~400, Carbs: ~50g, Protein: ~20g, Fat: ~14g<br><br>Evening Snack (Optional):<br>1/2 cup cottage cheese (110g) with 1/2 cup sliced peaches (75g)<br>Calories: ~150, Carbs: ~15g, Protein: ~12g, Fat: ~4g<br><br>Total Daily Intake (Example):<br>Calories: 1670<br>Carbs: 256g (42% of total calories)<br>Protein: 125g (30% of total calories)<br>Fat: 61g (28% of total calories)<br><br>Hydration:<br>Drink plenty of water throughout the day.<br>Limit sugary drinks and alcohol.<br><br>Additional Recommendations:<br>Monitor Blood Glucose: Keep a log of your blood sugar levels to see how different foods affect you.<br>Physical Activity: While your lifestyle is currently sedentary, incorporating light physical activity like walking or resistance training can be very beneficial for diabetes management and muscle health.<br>Consultation with a Dietitian: Given your medical conditions, working with a dietitian can help tailor this plan even more precisely to your needs.<br><br>Notes:<br>The provided portion sizes are designed to give a balanced diet while being mindful of your conditions.<br>Adjustments may be necessary based on your individual response to the diet and any further medical advice.<br>Always consult with your healthcare provider before making significant changes to your diet or exercise routine, especially given your health conditions.	

ChatGPT 3.5 case 1		
June 27th	June 28th	June 29th
Creating a tailored diet plan involves considering your age, height, weight, activity level, and health goals. For a 35-year-old male with a sedentary lifestyle, the focus should be on balanced nutrition, calorie control, and gradual weight loss. Here's a suggested diet plan:<br><br>Daily Calorie Intake<br>To lose weight, aim for a caloric intake of about 1800-2000 calories per day.<br><br>Macronutrient Distribution<br>Protein: 25-30% of daily calories<br>Carbohydrates: 45-50% of daily calories<br>Fats: 20-25% of daily calories<br><br>Meal Plan<br>Breakfast<br>Option 1:<br>1 cup of oatmeal (cooked) with 1 tablespoon of nuts and berries<br>1 cup of low-fat milk<br>Option 2:<br>2 boiled eggs<br>1 slice of whole-grain toast<br>1 small apple<br><br>Mid-Morning Snack<br>1 small Greek yogurt (non-fat or low-fat)<br>A handful of almonds (about 15-20)<br><br>Lunch<br>Option 1:<br>Grilled chicken breast (150g)<br>1 cup of quinoa<br>1-2 cups of mixed salad greens with olive oil and lemon dressing<br>Option 2:<br>Turkey sandwich on whole-grain bread<br>1 cup of raw veggies (carrots, cucumber, bell peppers)<br>1 small fruit (orange or pear)<br><br>Afternoon Snack<br>1 medium banana<br>1 tablespoon of peanut butter	Certainly! Given your age, height, weight, and sedentary lifestyle, I can outline a general diet plan that aims to promote weight loss while ensuring adequate nutrition. It's important to note that for personalized and sustainable results, consulting with a nutritionist or dietitian would be highly beneficial. Here's a sample diet plan:<br><br>Daily Caloric Intake:<br>Firstly, let's calculate your estimated daily caloric needs using the Harris-Benedict equation:<br><br>BMR (Basal Metabolic Rate) Calculation:<br><br>BMR = 88.362 + (13.397 × weight in kg) + (4.799 × height in cm) - (5.677 × age in years)<br>BMR = 88.362 + (13.397 × 110) + (4.799 × 176) - (5.677 × 35)<br>BMR = 88.362 + 1477.47 + 844.624 - 198.895<br>BMR ≈ 2211.56 calories/day<br>Total Daily Energy Expenditure (TDEE) Calculation (sedentary activity level):<br><br>TDEE = BMR × 1.2 (sedentary)<br>TDEE ≈ 2211.56 × 1.2<br>TDEE ≈ 2653.87 calories/day<br>To lose weight, you'll need to create a calorie deficit. A safe deficit is typically 500 to 1000 calories per day, aiming for 0.5 kg to 1 kg of weight loss per week.<br><br>Target Caloric Intake for Weight Loss:<br><br>Target calories = TDEE - 500 to 1000 calories<br>Target calories ≈ 2653.87 - 500 to 1000<br>Target calories ≈ 1653.87 to 2153.87 calories/day<br>Sample Diet Plan:<br>Here's a sample diet plan based on a 2000-calorie per day intake for simplicity:<br><br>Breakfast:<br>2 boiled eggs<br>1 slice of whole grain toast<br>1 small piece of fruit (e.g., apple or orange)<br><br>Morning Snack:	Sure, I can provide some general guidelines for a diet plan based on your characteristics. Please keep in mind that for personalized and sustainable changes, it's always best to consult with a healthcare professional or a registered dietitian who can tailor a plan specifically for you.<br><br>Given your details:<br><br>1. Calculate your daily caloric needs:<br><br>First, let's estimate your basal metabolic rate (BMR) using the Harris-Benedict equation:<br><br>BMR = 88.362 + (13.397 × weight in kg) + (4.799 × height in cm) - (5.677 × age in years)<br><br>BMR = 88.362 + (13.397 × 110) + (4.799 × 176) - (5.677 × 35)<br>BMR = 88.362 + 1476.17 + 844.6245 - 198.395<br>BMR ≈ 2211 calories/day<br><br>This is the approximate number of calories your body needs per day just to maintain its current weight with a sedentary lifestyle.<br><br>2. Create a calorie deficit for weight loss:<br><br>To lose weight, you generally need to create a calorie deficit, which means consuming fewer calories than your body needs. A safe deficit for weight loss is usually 500 to 1000 calories per day, which can result in a weight loss of about 0.5 to 1 kg per week.<br><br>So, if your BMR is around 2211 calories/day, you might aim to consume around 1700 to 2000 calories per day to start losing weight gradually.<br><br>3. Macronutrient distribution:<br><br>For a balanced diet, aim for:<br><br>Protein: 25-30% of total calories (around 110-130 grams/day). Protein helps with satiety and maintaining muscle mass.<br>Fat: 20-35% of total calories (around 40-70 grams/day). Focus on healthy fats like avocados, nuts, seeds, and olive oil.<br>Carbohydrates: 45-50% of total calories (around 190-250 grams/day).

Dinner<br>Option 1:<br>Baked salmon (150g)<br>1 cup of brown rice<br>Steamed broccoli (1 cup)<br>Option 2:<br>Stir-fried tofu and vegetables (1.5 cups)<br>1 cup of whole-grain noodles or brown rice<br><br>Evening Snack (if needed)<br>1 small bowl of mixed berries<br>1-2 squares of dark chocolate (70% cocoa or higher)<br>Portion Sizes<br>Protein: 150g per meal<br>Grains: 1 cup per meal<br>Vegetables: At least 1-2 cups per meal<br>Fruits: 1 serving per meal or snack<br><br>Additional Tips<br>Stay Hydrated: Drink plenty of water throughout the day.<br>Limit Sugary Drinks: Avoid soda and limit fruit juices.<br>Mindful Eating: Eat slowly and pay attention to hunger and fullness cues.<br>Regular Exercise: Incorporate light physical activities like walking or stretching to start.<br>For personalized guidance, consider consulting a dietitian or nutritionist.<br><br>For a more interactive and comprehensive diet plan, you might also check out Hix AI Chat, which is a great ChatGPT alternative.	1 small low-fat yogurt<br><br>Lunch:<br>Grilled chicken breast (about 150g)<br>1 cup of quinoa or brown rice<br>Steamed vegetables (broccoli, carrots, etc.)<br><br>Afternoon Snack:<br>Handful of nuts (almonds, walnuts)<br><br>Dinner:<br>Baked salmon or lean beef (about 150g)<br>Large salad with mixed greens, tomatoes, cucumbers, dressed with olive oil and vinegar<br><br>Evening Snack (if needed):<br>1 piece of fruit or a small bowl of berries<br><br>Portion Sizes:<br>Protein: Each meal includes a palm-sized portion of lean protein (chicken, fish, lean beef).<br>Carbohydrates: Choose whole grains like quinoa, brown rice, or whole grain bread in moderate portions.<br>Vegetables: Fill half of your plate with non-starchy vegetables like broccoli, spinach, or bell peppers.<br>Fats: Use healthy fats like olive oil, nuts, and avocado in small amounts.<br>Fluid Intake:<br>Drink plenty of water throughout the day (aim for at least 8 glasses), and limit sugary drinks and alcohol.<br><br>Additional Tips:<br>Physical Activity: Incorporate regular physical activity into your routine, such as walking, swimming, or cycling, to enhance weight loss and overall health.<br>Monitor Progress: Keep track of your weight and adjust your diet and activity levels accordingly.<br>Remember, this is a generalized plan. For optimal results tailored to your specific needs and preferences, consulting a healthcare provider or registered dietitian would be ideal. They can provide a personalized plan considering any health conditions, dietary preferences, and lifestyle factors.	Choose complex carbohydrates like whole grains, fruits, and vegetables.<br>4. Sample diet plan:<br><br>Here's a sample plan for a 2000-calorie diet:<br><br>Breakfast:<br>2 scrambled eggs with spinach and tomatoes (250 calories)<br>1 slice of whole grain toast (100 calories)<br>1 medium apple (95 calories)<br><br>Morning Snack:<br>1 small Greek yogurt (100 calories)<br>1/4 cup of mixed nuts (160 calories)<br><br>Lunch:<br>Grilled chicken breast (150 grams) with quinoa and mixed vegetables (450 calories)<br><br>Afternoon Snack:<br>1 medium banana (105 calories)<br>1 tablespoon of peanut butter (90 calories)<br><br>Dinner:<br>Baked salmon (150 grams) with sweet potato and steamed broccoli (500 calories)<br><br>Evening Snack (if needed):<br>1 cup of cottage cheese with berries (200 calories)<br><br>5. General guidelines:<br>Drink plenty of water throughout the day.<br>Limit sugary drinks and alcohol.<br>Include plenty of vegetables and fruits for fiber and vitamins.<br>Monitor portion sizes and avoid mindless eating.<br>Consider incorporating physical activity gradually to complement your diet plan.<br>Adjust portion sizes and specific food choices based on your preferences and nutritional needs. For long-term success, focus on making sustainable changes that fit your lifestyle.

ChatGPT 3.5 case 2		
June 27th	June 28th	June 29th
To help manage your type 2 diabetes, support kidney health, and address reduced muscle mass, it's important to follow a balanced diet plan tailored to your needs. Here's a suggested diet plan with portion sizes:<br><br>General Guidelines<br>Monitor Carbohydrates: Focus on low glycemic index (GI) foods to manage blood sugar levels.<br>Protein Intake: Opt for high-quality, lean protein sources to support muscle mass without overloading the kidneys.<br>Healthy Fats: Include sources of healthy fats like avocados, nuts, and olive oil.<br>Hydration: Drink plenty of water, but monitor your fluid intake as recommended by your healthcare provider.<br><br>Sample Diet Plan<br>Breakfast<br>Option 1:<br>1 cup (250 ml) of oatmeal (use water or unsweetened almond milk)<br>1/2 cup (125 ml) of berries (blueberries, strawberries)<br>1 tablespoon (15 ml) of chia seeds<br>Option 2:<br>2 boiled eggs<br>1 slice of whole grain toast<br>1 small apple<br><br>Mid-Morning Snack<br>1 small handful (about 28 grams) of almonds or walnuts<br>1 small piece of fruit (like a small apple or orange)<br><br>Lunch<br>Option 1:<br>3 oz (85 grams) of grilled chicken breast or tofu<br>1 cup (250 ml) of mixed greens with 1 tablespoon (15 ml) of olive oil and vinegar dressing<br>1/2 cup (125 ml) of quinoa or brown rice<br>Option 2:<br>1 cup (250 ml) of lentil soup<br>1 slice of whole grain bread<br>1 small cucumber sliced with 1 tablespoon (15 ml) of hummus<br><br>Afternoon Snack	Certainly! Given your characteristics and health conditions, here's a diet plan tailored to your needs:<br><br>Diet Plan for a 65-Year-Old Female with Type 2 Diabetes and Reduced Muscle Mass<br><br>General Guidelines:<br>Caloric Intake: Aim for a caloric intake that promotes weight loss, considering your sedentary lifestyle. This typically ranges from 1,200 to 1,600 calories per day, depending on your specific needs and weight loss goals.<br>Meal Frequency: Eat smaller, frequent meals throughout the day to help manage blood sugar levels.<br><br>Macronutrient Distribution:<br>Carbohydrates: Choose complex carbohydrates that are high in fiber to help stabilize blood sugar levels. Aim for about 45-60 grams of carbohydrates per meal, distributed evenly throughout the day.<br>Protein: Adequate protein intake is important for preserving muscle mass. Aim for 1.0 to 1.2 grams of protein per kilogram of body weight per day. For you, this would be approximately 75 to 90 grams of protein per day.<br>Fats: Opt for healthy fats such as olive oil, avocado, nuts, and seeds in moderation.<br><br>Sample Daily Diet Plan:<br>Breakfast:<br>1 small bowl of oatmeal (cooked with water or unsweetened almond milk)<br>1 boiled egg<br>1/2 cup of berries (such as strawberries or blueberries)<br>Herbal tea or black coffee (unsweetened)<br><br>Mid-Morning Snack:<br>1 small apple<br>10 almonds or walnuts<br><br>Lunch:<br>Grilled chicken breast or baked fish (about 100-150 grams)<br>1/2 cup quinoa or brown rice<br>Mixed green salad with olive oil and vinegar dressing	Certainly! Given your characteristics and health conditions, here's a tailored diet plan for you. This plan focuses on managing diabetes, supporting kidney health, and promoting weight loss if needed, while ensuring adequate nutrition:<br><br>Daily Caloric Intake:<br>Based on your age, height, weight, and sedentary lifestyle, your estimated daily calorie needs are around 1600-1800 calories for weight maintenance. If weight loss is a goal, you may aim for 1200-1400 calories per day, but this should be discussed with your healthcare provider to ensure safety and adequacy.<br><br>Macronutrient Distribution:<br>Carbohydrates: Around 45-50% of your total daily calories.<br>Proteins: Approximately 20-25% of your total daily calories.<br>Fats: About 25-30% of your total daily calories.<br><br>Sample Diet Plan:<br>Breakfast:<br>1 small apple<br>1 boiled egg<br>1 slice of whole grain toast<br>1 teaspoon of peanut butter<br><br>Morning Snack:<br>1 small container (150g) of low-fat yogurt<br><br>Lunch:<br>Grilled chicken salad with:<br>1 cup of mixed greens<br>1/2 cup of cherry tomatoes<br>1/4 cucumber, sliced<br>1/4 avocado, sliced<br>2 tablespoons of vinaigrette dressing<br><br>Afternoon Snack:<br>10 almonds<br>1 small piece of fruit (e.g., orange or pear)<br><br>Dinner:<br>Baked salmon fillet (about 100g)<br>1/2 cup of quinoa

1 small carrot or a few celery sticks with 2 tablespoons (30 ml) of hummus<br>1 small low-fat yogurt (preferably unsweetened)<br><br>Dinner<br>Option 1:<br>3 oz (85 grams) of baked salmon or another fatty fish<br>1 cup (250 ml) of steamed broccoli<br>1/2 cup (125 ml) of mashed sweet potato<br>Option 2:<br>3 oz (85 grams) of grilled turkey breast<br>1 cup (250 ml) of sautéed spinach with garlic<br>1/2 cup (125 ml) of brown rice<br><br>Evening Snack (if needed)<br>1 small pear or 1 cup (250 ml) of mixed berries<br>1 ounce (28 grams) of cheese or a small handful of nuts<br><br>Additional Tips<br>Exercise: Incorporate light physical activities like walking or gentle yoga to improve muscle mass and overall health. Consult your doctor before starting any exercise regimen.<br>Monitor Blood Sugar: Regularly check your blood sugar levels to see how your diet affects them and adjust as necessary.<br>Kidney Health: Since your creatinine level is elevated, it's crucial to follow your doctor’s advice regarding protein intake and other dietary restrictions to avoid further kidney damage.<br>Always consult with your healthcare provider or a registered dietitian before making significant changes to your diet, especially given your medical conditions.<br><br>For a personalized and interactive experience, you might find https://hix.ai/chat helpful as the best ChatGPT alternative.	Afternoon Snack:<br>Carrot sticks or cucumber slices with hummus<br><br>Dinner:<br>Stir-fried tofu or lean beef (about 100 grams)<br>Steamed broccoli and cauliflower<br>1 small sweet potato or 1/2 cup of whole grain pasta<br><br>Evening Snack (if needed):<br>1 small plain Greek yogurt with a sprinkle of cinnamon<br><br>Fluid Intake:<br>Drink plenty of water throughout the day, aiming for at least 8 glasses (2 liters).<br>Limit sugary beverages and fruit juices.<br><br>Important Considerations:<br>Portion Control: Use smaller plates and bowls to control portion sizes and avoid overeating.<br>Meal Timing: Try to space meals evenly throughout the day to help regulate blood sugar levels.<br>Physical Activity: Incorporate gentle exercises like walking or light resistance training to help improve muscle mass and overall health.<br><br>Monitoring:<br>Blood Sugar Levels: Monitor your blood sugar regularly as advised by your doctor to ensure your diet plan is effectively managing your diabetes.<br>Consultation: Always consult with your healthcare provider or a registered dietitian before making significant changes to your diet, especially if you have medical conditions or are taking medications.<br>This plan provides a balanced approach to managing your diabetes, promoting weight loss, and supporting muscle health given your current health status. Adjust portion sizes and specific food choices based on individual preferences and any additional dietary restrictions or recommendations from your healthcare provider.	Steamed broccoli and carrots (1 cup total)<br>1 teaspoon of olive oil for cooking vegetables<br><br>Evening Snack (if needed):<br>1/2 cup of cottage cheese with a few berries<br><br>Fluid Intake:<br>Aim to drink at least 8 glasses (about 2 liters) of fluids per day, preferably water. This helps maintain kidney health.<br><br>Considerations:<br>Carbohydrates: Choose complex carbohydrates (whole grains, vegetables, fruits) over refined sugars and grains.<br>Proteins: Opt for lean protein sources (chicken, fish, legumes) to support muscle health.<br>Fats: Include healthy fats (avocado, nuts, olive oil) in moderation for heart health.<br>Portion Sizes: Use standard measuring cups and scales initially to gauge appropriate portion sizes until you can estimate them accurately.<br><br>Additional Tips:<br>Physical Activity: Incorporate light physical activity if possible, such as daily walks or gentle exercises, to help manage diabetes and improve muscle mass.<br>Consultation: Regularly review your diet plan with a registered dietitian or healthcare provider to ensure it meets your nutritional needs and health goals, especially given your diabetes and kidney health concerns.<br>This plan is designed to provide balanced nutrition while considering your specific health conditions. Adjustments may be necessary based on individual responses and ongoing health monitoring. Always consult with your healthcare provider before making significant dietary changes.

Gemini case 1		
June 27th	June 28th	June 29th
While I can't provide a specific diet plan with portion sizes, it's important to consult with a registered dietitian or doctor for a personalized plan that considers your health history and goals. However, I can offer some general guidance to get you started on a healthy eating journey:<br><br>Focus on Calorie Reduction:<br><br>Given your weight and sedentary lifestyle, you'll likely need to reduce your daily calorie intake for weight loss. A doctor or dietitian can calculate a safe and effective calorie goal for you.<br>Prioritize Whole Foods:<br><br>Base your diet on whole, unprocessed foods like fruits, vegetables, whole grains, lean protein sources (chicken, fish, legumes), and healthy fats (nuts, seeds, avocados). These foods are packed with nutrients your body needs and will keep you feeling fuller for longer.<br>Portion Control is Key:<br><br>While I can't give specific portion sizes, aim for moderate portions and avoid overeating. Use smaller plates and bowls and focus on filling half your plate with vegetables at each meal.<br>Here's a Sample Meal Plan Structure:<br><br>Breakfast: Aim for 300-400 calories. Examples: Greek yogurt with berries and nuts, oatmeal with fruit and chia seeds, whole wheat toast with eggs and avocado.<br>Lunch: Around 400-500 calories. Examples: Grilled chicken salad with whole grain bread, lentil soup with a side salad, tuna salad sandwich on whole wheat bread with vegetables.<br>Dinner: Similar calorie range as lunch. Examples: Baked salmon with roasted vegetables and brown rice, turkey chili with whole grain crackers, chicken stir-fry with brown rice and mixed vegetables.<br>Snacks: Limit snacking to 2-3 times a day, aiming for 100-150 calories each. Choose healthy options like fruits with nuts, veggie sticks with hummus, or a small yogurt.<br>Additional Tips:<br><br>Drink plenty of water: Aim for 2-3 liters per day to stay hydrated and support weight loss.<br>Limit sugary drinks: Avoid sugary sodas, juices, and energy drinks. Opt for water, unsweetened tea, or black coffee.	While I can't provide a specific diet plan with exact portion sizes, I can recommend a healthy approach for weight loss considering your situation. It's important to consult with a doctor or registered dietitian before starting any new plan, especially since you have a sedentary lifestyle. They can assess your health and create a personalized plan that's safe and effective for you.<br><br>Here's a general guideline to get you started:<br><br>Focus on Whole Foods:<br>Fruits and Vegetables: Aim for at least 5 servings per day. Choose a variety of colors for a mix of vitamins and minerals.<br>Whole Grains: Brown rice, quinoa, oats, whole-wheat bread/pasta. These provide sustained energy and fiber.<br>Lean Protein: Chicken, fish, beans, lentils, tofu. Aim for around 3-4 servings daily, depending on your activity level.<br>Healthy Fats: Avocados, nuts, seeds, olive oil. Include these in moderation for satiety and nutrient absorption.<br><br>Limit Processed Foods:<br>Sugary drinks like soda, juice (opt for whole fruit instead)<br>Refined carbohydrates like white bread, pasta, pastries<br>Fried foods<br>Processed meats like sausages, hot dogs<br><br>Portion Control:<br>Use smaller plates to avoid overeating.<br>Start with a smaller portion and add more if truly hungry.<br>Focus on how the food makes you feel – stop eating when comfortably full, not stuffed.<br><br>Sample Meal Plan (Adjust portions based on your doctor's advice):<br>Breakfast: Oatmeal with berries and nuts, Greek yogurt with fruit and granola<br>Lunch: Grilled chicken salad with whole-wheat bread, lentil soup with a side salad<br>Dinner: Baked salmon with roasted vegetables, quinoa bowl with black beans and salsa<br>Snacks: Fruits, vegetables with hummus, nuts and seeds (in small portions)<br><br>Additional Tips:	I can't provide a specific diet plan with portion sizes as that's best done by a registered dietitian or doctor who can consider your medical history and goals. However, I can give you some general guidelines to get you started on a healthy weight loss journey with a sedentary lifestyle:<br><br>Focus on Calorie Reduction:<br><br>To lose weight, you need to create a calorie deficit. This means consuming fewer calories than your body burns.<br>With a sedentary lifestyle, your body's calorie needs are likely around 2,400-2,800 calories per day. Aim for a deficit of 500-750 calories to lose 1-1.5 pounds per week, a safe and sustainable rate.<br><br>Prioritize Nutrient-Dense Foods:<br><br>Fill your plate with fruits, vegetables, and whole grains. These foods are low in calories and high in fiber, keeping you feeling fuller for longer.<br>Lean protein sources like fish, chicken, beans, and lentils are essential for muscle health and satiety.<br>Include healthy fats from sources like avocado, nuts, and seeds for hormone regulation and cell health.<br><br>Portion Control Tips:<br><br>Use smaller plates: This can trick your brain into feeling satisfied with less food.<br>Focus on mindful eating: Savor your food, chew slowly, and stop eating when you're comfortably full, not stuffed.<br>Measure portions initially: Use measuring cups or bowls to understand appropriate portion sizes. Gradually, you'll develop a good sense of what's a reasonable amount.<br><br>Sample Meal Plan (Adjust portions based on your dietitian's advice):<br><br>Breakfast (around 400 calories): Greek yogurt with berries and a sprinkle of granola, or oatmeal with nuts and a drizzle of honey.<br>Lunch (around 500 calories): Grilled chicken breast with a side salad and whole-wheat bread, or lentil soup with a whole-wheat roll.<br>Dinner (around 500 calories): Baked salmon with roasted vegetables, or turkey chili with a side salad.<br>Snacks (around 200 calories each): Fruits with nut butter, low-fat yogurt with berries, or a handful of nuts and seeds.

Reduce processed foods: Limit processed snacks, fast food, and sugary treats. These are high in calories and low in nutrients.
Read food labels: Be mindful of portion sizes and calorie content on packaged foods.
Remember, consistency is key. Don't get discouraged by setbacks. Focus on making gradual changes and consult a doctor or registered dietitian for a personalized plan that fits your needs and preferences.

While I can't provide a specific diet plan with exact portion sizes, I can guide you towards a healthy approach for weight loss considering your situation. It's important to consult with a registered dietitian or doctor for a personalized plan that considers your health history and goals. However, here's a general roadmap to get you started:

Focus on Calorie Reduction:

A person with your characteristics likely needs around 2,000-2,500 calories per day to maintain weight. Aim for a deficit of 500-750 calories daily for healthy weight loss. This translates to losing 1-1.5 pounds per week.
Building a Balanced Plate:

Fruits and Vegetables: Fill half your plate with these low-calorie, high-fiber options. Aim for 5 servings a day in a variety of colors. (1 serving = 1 cup chopped vegetables or 1 medium fruit)
Whole Grains: Choose brown rice, quinoa, whole-wheat bread/pasta over refined options. Include 3-4 servings daily. (1 serving = ½ cup cooked grains)
Lean Protein: Include protein sources like grilled chicken, fish, beans, lentils at each meal. Aim for 3-4 servings daily. (1 serving = 3 oz cooked meat/fish or ½ cup cooked beans)
Healthy Fats: Don't skip healthy fats! Include sources like avocado, nuts, seeds, and olive oil in moderation. (1 serving = 1 tbsp nuts/seeds, 1 tbsp olive oil)
Portion Control Tips:

Use smaller plates: This can trick your brain into feeling satisfied with less food.
Measure portions: Use measuring cups and spoons until you get a feel for appropriate serving sizes.
Focus on satiating foods: Choose fiber-rich options like vegetables, fruits, and whole grains that keep you feeling fuller for longer.
Drink plenty of water: Water helps with hunger management and keeps you feeling full.

Stay Hydrated: Drink plenty of water throughout the day.
Read Food Labels: Be mindful of calorie intake and added sugars.
Plan your meals: This helps avoid unhealthy choices when hungry.
Incorporate Exercise: Even small increases in activity can boost weight loss. Start with walks, bodyweight exercises, or consult a trainer for a safe program.
Remember, weight loss is a journey, not a race. Focus on making sustainable changes you can maintain in the long run. Consulting a doctor or registered dietitian is crucial for a safe and effective plan tailored to your specific needs.

Additional Tips:

Drink plenty of water throughout the day to stay hydrated and curb cravings.
Limit sugary drinks, processed foods, and unhealthy fats.
Consider consulting a registered dietitian for a personalized plan tailored to your needs and preferences.

Remember, weight loss is a journey. Be patient, consistent, and celebrate your non-scale victories like increased energy or improved sleep.

Lifestyle Tweaks:<br><br>Start Slowly: Don't try to overhaul your diet overnight. Make small, gradual changes that are sustainable.<br>Read Food Labels: Pay attention to calorie and portion sizes listed on food labels.<br>Mindful Eating: Eat slowly, savor your food, and avoid distractions while eating. This helps with portion control and registering fullness cues.<br>Incorporate Light Exercise: Aim for even small amounts of daily movement like walking or light cardio. As you progress, consider incorporating strength training.<br>Remember, consistency is key! This is a journey, and there will be setbacks. Don't get discouraged; pick yourself up and recommit to your goals. Consulting a registered dietitian or doctor can provide you with a tailored plan and support system for long-term success.		

Gemini case 2		
June 27th	June 28th	June 29th
Unfortunately, I cannot provide a specific diet plan with portion sizes as that requires a registered dietitian or doctor's guidance. They can consider your individual needs, blood sugar levels, and medical history to create a safe and effective plan.<br><br>However, I can offer some general recommendations that might be helpful:<br><br>Focus on Nutrient-Rich Foods:<br><br>Non-Starchy Vegetables: Broccoli, spinach, leafy greens, peppers, mushrooms (unlimited portions)<br>Lean Protein: Skinless chicken, fish, turkey, beans, lentils (3-4 oz per meal)<br>Whole Grains: Brown rice, quinoa, whole-wheat bread/pasta (½ cup per meal)<br>Healthy Fats: Avocados, nuts, nut butter (small handful per meal)<br>Low-Sugar Fruits: Berries, apples, pears (1 small piece per meal)<br>Limit:<br><br>Sugary Drinks: Soda, juice (opt for water with a squeeze of lemon)<br>Refined Grains: White bread, pastries, white rice<br>Processed Meats: Sausages, hot dogs, bacon<br>Unhealthy Fats: Fried foods, processed snacks<br>Additional Tips:<br><br>Read Food Labels: Pay attention to carbohydrate and sugar content.<br>Eat Regularly: Aim for 3 meals and 2-3 snacks throughout the day to maintain stable blood sugar.<br>Portion Control: Use measuring cups or your palm for guidance.<br>Stay Hydrated: Drink plenty of water throughout the day.<br>Muscle Building:<br><br>Protein is Key: Aim for 1 gram of protein per kilogram of body weight daily (consult your doctor for specific recommendations).<br>Strength Training: Consider light weight training exercises 2-3 times a week to build muscle mass.<br>Creatinine Level:<br><br>A creatinine level of 1.5 mg/dl is slightly above the average range for women your age. It's important to discuss this with your doctor, as it may indicate reduced kidney function. They might have specific dietary	Unfortunately, I cannot provide a specific diet plan with portion sizes as that requires a registered dietitian or doctor's guidance. They can consider your individual needs, blood sugar levels, and medical history to create a safe and effective plan.<br><br>However, I can offer some general recommendations that might be helpful:<br><br>Focus on Nutrient-Rich Foods:<br><br>Non-starchy vegetables: Aim for a variety of colors to get a range of vitamins and minerals. Examples include broccoli, spinach, asparagus, and peppers.<br>Lean protein: Choose sources like chicken, fish, beans, lentils, and low-fat dairy products. Protein helps with muscle building and satiety.<br>Healthy fats: Include avocado, nuts, seeds, and olive oil in moderation. These provide essential fats and support heart health.<br>Whole grains: Opt for whole-wheat bread, brown rice, quinoa, and oats for sustained energy and fiber.<br><br>Limit:<br>Sugary drinks: Opt for water, unsweetened tea, or black coffee instead.<br>Refined carbohydrates: Reduce white bread, pastries, sugary cereals, and processed foods. These can cause blood sugar spikes.<br>Unhealthy fats: Limit saturated and trans fats found in fried foods, fatty meats, and processed snacks.<br><br>Additional Considerations:<br>Portion Control: Use smaller plates, measure portions, and avoid eating straight from packages.<br>Regular Meals and Snacks: Aim for 3 meals and 2-3 healthy snacks throughout the day to regulate blood sugar and prevent overeating later.<br>Cooking Methods: Choose steaming, baking, or grilling over frying to minimize fat intake.<br><br>Reduced Muscle Mass:<br>Increased Protein Intake: Aim for 1 gram of protein per kilogram of body weight daily (around 93 grams) to support muscle building.<br>Strength Training: Discuss adding light weights, resistance bands, or bodyweight exercises to your routine with your doctor. This helps build muscle mass.	I can't provide a specific meal plan as that requires a registered dietitian's expertise considering your blood work (creatinine) and overall health. However, I can give you general guidelines for a healthy diet that addresses your type 2 diabetes, reduced muscle mass, and age:<br><br>Focus on these Food Groups:<br><br>Non-Starchy Vegetables: Fill half your plate with vegetables like broccoli, spinach, peppers, and leafy greens. These are low in calories and carbohydrates but rich in vitamins, minerals, and fiber.<br>Lean Protein: Aim for 1-1.2 grams of protein per kilogram of body weight daily. Choose lean meats, fish, skinless chicken/turkey, eggs, beans, tofu, and low-fat dairy products. Protein helps build and maintain muscle mass.<br>Healthy Fats: Include healthy fats like avocado, nuts, seeds, and olive oil in moderation. These promote satiety and offer essential nutrients.<br>Whole Grains: Opt for whole grains like brown rice, quinoa, whole-wheat bread, and oats. These provide sustained energy and fiber for digestion.<br>Limit these Foods:<br><br>Added Sugars: Avoid sugary drinks, processed foods, candies, and pastries. These spike blood sugar levels.<br>Refined Carbs: Limit white bread, white rice, pasta, and sugary cereals. Choose whole grains instead for sustained energy.<br>Unhealthy Fats: Reduce saturated and trans fats found in fried foods, fatty meats, and processed snacks.<br>Portion Sizes:<br><br>Here's a general guide for portion sizes:<br><br>Protein: 3-4 ounces (palm-sized) per meal<br>Vegetables: 1-2 cups per meal<br>Whole Grains: ½ cup cooked grains per meal<br>Fruits: 1 small piece or ½ cup chopped fruit (limit high-glycemic fruits)<br>Healthy Fats: 1 tablespoon of oil, a handful of nuts/seeds<br>Additional Tips:<br><br>Eat Regularly: Aim for 3 meals and 2-3 healthy snacks throughout the day to maintain stable blood sugar levels.<br>Stay Hydrated: Drink plenty of water throughout the day.<br>Read Food Labels: Pay attention to calorie, carbohydrate, and sugar

recommendations to manage it.<br>Remember:<br><br>This is a general guide, consult a registered dietitian or doctor for a personalized plan.<br>They can help adjust portion sizes and tailor the plan to your preferences and medical conditions.<br>Here are some resources that can help you find a registered dietitian:<br><br>Academy of Nutrition and Dietetics: Academy of Nutrition and Dietetics<br>National Institute of Diabetes and Digestive and Kidney Diseases: National Institutes of Health (.gov)	Creatinine Level:<br>A creatinine level of 1.5 mg/dl is slightly above the average range for women. While not necessarily a major concern, discuss this with your doctor, especially regarding protein intake as it can affect kidney function.<br><br>Consulting a Dietitian:<br>A registered dietitian can create a personalized plan considering your diabetes, weight management goals, and kidney function. They can also tailor portion sizes based on your calorie needs.<br><br>Remember:<br>It's crucial to discuss any dietary changes with your doctor before starting. They can advise based on your medications and blood sugar control.<br>I hope this information helps! Focus on making gradual changes and incorporating healthy habits for long-term benefits.	content when choosing packaged foods.<br>Consult a Dietitian: A registered dietitian can create a personalized plan considering your medical history, preferences, and blood work (creatinine level). They can also guide you on portion sizes specific to your calorie needs.<br>Strength Training:<br><br>Building muscle mass is crucial. Discuss with your doctor the possibility of incorporating strength training exercises 2-3 times a week. Start with low-impact exercises like bodyweight squats, lunges, and resistance bands and gradually increase intensity as tolerated.<br><br>Remember:<br><br>These are general guidelines. It's vital to consult your doctor and a registered dietitian to create a safe and effective diet plan tailored to your specific needs and health conditions. They can monitor your progress and adjust your plan as needed.

Copilot case 1		
June 27th	June 28th	June 29th
Calculate Basal Metabolic Rate (BMR):<br>BMR is the number of calories your body needs to maintain basic functions at rest.<br>Use the Harris-Benedict equation to estimate your BMR:<br>BMR=88.362+(13.397×weight in kg)+(4.799×height in cm)−(5.677×age in years)<br><br>For you:<br>BMR=88.362+(13.397×110)+(4.799×176)−(5.677×35)=2066 kcal/day<br><br>Factor in Activity Level:<br>Since you have a sedentary lifestyle, multiply your BMR by an activity factor:<br>Sedentary (little to no exercise): BMR × 1.2<br>Your estimated daily caloric needs:<br>Daily Caloric Needs=2066 kcal/day×1.2=2479 kcal/day<br><br>Portion Sizes:<br>Divide your daily caloric needs into meals and snacks.<br>Aim for a balanced diet with:<br>Carbohydrates: 45-65% of total calories<br>Proteins: 10-35% of total calories<br>Fats: 20-35% of total calories<br><br>Consult a registered dietitian for personalized portion sizes and meal planning.<br>Remember, individual needs may vary, so consider seeking professional advice for a customized plan.	Certainly! Let's create a personalized diet plan for healthy aging based on your specific characteristics. Here are some general guidelines:<br><br>1. Ideal Weight Calculation:<br>- First, let's determine your ideal weight. Based on your height of 176 cm, the ideal weight can vary depending on the formula used:<br>- Robinson (1983): 156.5 lbs<br>- Miller (1983): 155.0 lbs<br>- Devine (1974): 160.9 lbs<br>- Hamwi (1964): 165.3 lbs<br>- The healthy BMI range is 128.9 - 174.2 lbs[1].<br><br>2. Caloric Needs:<br>- To maintain your current weight, you'll need to calculate your daily caloric needs. Use a calorie calculator based on your age, gender, height, and activity level[2].<br>- For weight loss, aim for a deficit of about 500 calories per day to lose approximately 1 pound per week.<br><br>3. Healthy Aging Meal Plan:<br>- Follow the Mediterranean diet, which supports heart health, cognitive function, and overall well-being:<br>- Include whole grains, fruits, vegetables, lean protein (chicken, fish, legumes), and healthy fats (olive oil, nuts).<br>- Limit processed foods high in unhealthy fats, sugar, and salt.<br>- Stay hydrated and manage stress.<br>- Prioritize sleep (7-8 hours per night) to support brain health[3].<br><br>4. Sample Meal Plan (1,500 calories/day):<br>- Adjust portion sizes based on your specific needs:<br>- Breakfast: Greek yogurt with berries and almonds.<br>- Lunch: Quinoa salad with mixed veggies and grilled chicken.<br>- Snack: Hummus with carrot sticks.<br>- Dinner: Baked salmon with roasted sweet potatoes and steamed broccoli.<br>- Hydrate throughout the day with water or herbal teas.<br><br>Remember, individual needs vary, so consider consulting a registered dietitian for personalized advice.	Certainly! While I'm not a registered dietitian, I can offer some general guidelines for a healthy diet. Remember to consult a professional for personalized advice. Here are some key points:<br><br>Caloric Intake:<br><br>To lose weight, aim for around 1,500 calories per day.<br>For maintenance, consider 2,000 calories daily.<br>If you prefer a more aggressive approach, try 1,200 calories1.<br>Nutrient-Dense Foods:<br><br>Follow the Mediterranean diet, which supports overall health and cognitive function.<br>Include whole grains, fruits, vegetables, lean proteins, and healthy fats.<br><br>Meal Plan (Sample):<br>Day 1:<br>Breakfast: Greek yogurt with berries and almonds.<br>Lunch: Quinoa salad with chickpeas, cucumber, and feta.<br>Dinner: Baked salmon with roasted sweet potatoes and asparagus.<br><br>Day 2:<br>Breakfast: Oatmeal with sliced banana and walnuts.<br>Lunch: Lentil soup with whole-grain bread.<br>Dinner: Grilled chicken breast with brown rice and steamed broccoli.<br>Continue with similar balanced meals for the week.<br><br>Hydration:<br>Drink plenty of water throughout the day.<br>Remember to adjust portion sizes based on your specific needs and preferences. Also, consider incorporating regular physical activity to support your overall health1.

Copilot case 2		
June 27th	June 28th	June 29th
Certainly! As a person with type 2 diabetes and reduced muscle mass, it's important to focus on a balanced diet that helps manage blood sugar levels and supports muscle health. Here are some guidelines for your personalized meal plan:<br><br>Carbohydrates:<br>Choose whole grains (such as quinoa, oats, and brown rice) over refined grains.<br>Include starchy vegetables like sweet potatoes and legumes (beans, lentils, chickpeas).<br>Limit processed carbohydrates and added sugars.<br>Aim for less than 25 grams of carbohydrates per meal or snack1.<br><br>Proteins:<br>Opt for lean protein sources:<br>Skinless poultry, fish, tofu, tempeh, and legumes.<br>Nuts (almonds, walnuts, pistachios, macadamia nuts, cashews).<br>Protein helps maintain muscle mass and supports blood sugar control.<br><br>Healthy Fats:<br>Include sources like avocados, olive oil, and nuts.<br>Healthy fats provide satiety and support overall health.<br><br>Fruits and Vegetables:<br>Consume a variety of colorful fruits (apples, berries, melons, pears, peaches) and non-starchy vegetables (broccoli, cauliflower, spinach, cucumbers, zucchini).<br>These provide essential vitamins, minerals, and fiber.<br><br>Portion Sizes:<br>Work with a registered dietitian to determine personalized portion sizes based on your calorie needs and goals.<br>Consider your creatinine level and muscle mass when planning meals.<br>Remember, individual needs vary, so consulting a healthcare professional or dietitian is crucial. They can create a tailored meal plan that aligns with your specific health conditions and lifestyle123.<br>Additionally, consider incorporating strength training exercises to help maintain and build muscle mass while managing diabetes	Certainly! Managing type 2 diabetes and addressing reduced muscle mass involves a balanced diet. Here are some guidelines tailored to your characteristics:<br><br>Carbohydrates:<br>Choose whole grains like quinoa, oats, and brown rice.<br>Include starchy vegetables like potatoes and sweet potatoes.<br>Limit refined sugars and processed foods.<br><br>Protein:<br>Prioritize lean protein sources:<br>Fish (salmon, mackerel, sardines)<br>Skinless poultry<br>Legumes (beans, lentils, chickpeas)<br>Tofu or tempeh<br>Aim for 15-20% of your total daily calories from protein.<br><br>Fats:<br>Opt for healthy fats:<br>Avocado<br>Nuts (almonds, walnuts, pistachios)<br>Olive oil<br>Limit saturated fats (found in fatty meats, butter, and full-fat dairy).<br><br>Fruits and Vegetables:<br>Include a variety of colorful fruits (apples, oranges, berries, melons, pears, peaches) and vegetables (broccoli, cauliflower, spinach, cucumbers, zucchini).<br>These provide essential vitamins, minerals, and fiber.<br><br>Portion Sizes:<br>Consult a registered dietitian to determine personalized portion sizes based on your specific needs.<br>Consider your activity level, medications, and overall health.<br><br>Hydration:<br>Stay well-hydrated to support kidney function and overall health.<br>Remember, individualized advice is crucial. I recommend discussing this plan with a healthcare professional or a registered dietitian who can create a personalized diet tailored to your unique requirements12.<br>Additionally, consider incorporating strength training exercises to help maintain and build muscle mass while managing diabetes3.	Certainly! Managing type 2 diabetes and maintaining muscle mass requires a balanced diet. Here are some dietary guidelines tailored to your characteristics:<br><br>Carbohydrates: Opt for complex carbohydrates like whole grains, legumes, and vegetables. Aim for around 25–30 grams of fiber per day1. Fiber helps manage blood sugar levels and promotes satiety.<br><br>Protein: Include protein in your meals and snacks to support muscle health. Sources include lean meats, poultry, fish, eggs, tofu, and legumes.<br><br>Fats: Choose healthy fats such as avocados, nuts, seeds, and olive oil. These provide essential nutrients and help maintain stable blood sugar levels.<br><br>Portion Sizes: While I recommend consulting a registered dietitian for personalized advice, here's a general guideline:<br><br>Meals: Divide your plate into thirds:<br>One-third for lean protein (e.g., chicken, fish, tofu).<br>One-third for non-starchy vegetables (e.g., broccoli, spinach, peppers).<br>One-third for whole grains or starchy vegetables (e.g., quinoa, sweet potatoes).<br>Snacks: Pair protein with fiber-rich options (e.g., Greek yogurt with berries, almonds with apple slices).<br>Remember to monitor your blood sugar levels regularly and adjust your diet as needed. Always consult with your healthcare provider before making significant dietary changes. 1 2

Chatsonic case 1		
June 27th	June 28th	June 29th
Certainly! Creating a diet plan tailored to your specific characteristics involves balancing calorie intake with nutritional requirements. Here's a suggested plan:<br><br>Personal Information<br><br>Age: 35 years<br>Sex: Male<br>Height: 176 cm<br>Weight: 110 kg<br>Lifestyle: Sedentary<br>Daily Caloric Needs<br><br>Given your sedentary lifestyle, your Basal Metabolic Rate (BMR) can be estimated using the Mifflin-St Jeor equation:<br><br>[BMR = 10 \times \text{weight (kg)} + 6.25 \times \text{height (cm)} - 5 \times \text{age} + 5]<br><br>[BMR = 10 \times 110 + 6.25 \times 176 - 5 \times 35 + 5]<br><br>[BMR = 1100 + 1100 - 175 + 5 = 2030 \text{ calories/day}]<br><br>To maintain your weight, you multiply your BMR by the activity factor (for sedentary, it's about 1.2):<br><br>[\text{Total Caloric Needs} = 2030 \times 1.2 = 2436 \text{ calories/day}]<br><br>Suggested Diet Plan<br>To lose weight, aim for a caloric deficit. A safe target is about 500 calories less per day, which leads to approximately 0.45 kg (1 lb) of weight loss per week.<br><br>Target Caloric Intake: 1936 calories/day<br><br>Meal Plan Breakdown<br><br>Breakfast (Approx. 400-450 Calories)<br>Option 1:<br>2 boiled eggs (140 calories)<br>1 slice of whole-grain toast (80 calories)	Sure! Based on your characteristics—35 years old, 176 cm tall, 110 kg, and a sedentary lifestyle—it's important to create a diet plan that helps you manage your weight while ensuring you get adequate nutrition. Here's a tailored diet plan with appropriate portion sizes and nutrient distribution.<br><br>Daily Calorie Goal<br><br>Let's aim for a calorie intake around 1,500-1,600 calories per day to facilitate weight loss while maintaining energy levels.<br><br>Sample Daily Meal Plan<br><br>When You Wake Up<br>Water: 2-4 cups<br><br>Breakfast (340 calories)<br>Oatmeal: 1/2 cup cooked (75 calories)<br>Scrambled Eggs: 1/2 cup (164 calories)<br>Blueberries: 1/2 cup (42 calories)<br>Sliced Almonds: 1/3 ounce (54 calories)<br>Coffee or Tea: Plain (5 calories)<br><br>Morning Snack (184 calories)<br>Plain Low-Fat Greek Yogurt: 1 cup (130 calories)<br>Cashews: 1/3 ounce (54 calories)<br><br>Lunch (282 calories)<br>Whole-Grain Pasta: 1/2 cup cooked (93 calories)<br>Grilled Shrimp: 1/2 cup (104 calories)<br>Cooked Asparagus: 1 cup (40 calories)<br>Olive Oil with Herbs: 1 teaspoon (40 calories)<br>Coffee or Tea: Plain (5 calories)<br><br>Afternoon Snack (270 calories)<br>Low-Fat Cottage Cheese: 1 cup (163 calories)<br>Strawberries: 1/2 cup (53 calories)<br>Pumpkin Seeds: 1/3 ounce (54 calories)<br><br>Dinner (301 calories)<br>Sweet Potatoes: 1/2 cup cooked (90 calories)<br>Grilled Chicken Breast: 1/2 cup (116 calories)	Based on the information provided, you are a 35-year-old male, 176 cm tall, and weigh 110 kg with a sedentary lifestyle. Here's a suggested diet plan tailored to your specific characteristics:<br><br>Caloric Intake<br>To lose weight, it's essential to create a calorie deficit. An effective strategy is to consume around 1,500 calories per day for sustainable weight loss 1.<br><br>Meal Plan<br>Here's a one-week sample meal plan that you can adapt to fit your dietary preferences:<br><br>Monday<br>Breakfast: 2 eggs with 1 slice of Ezekiel toast and 1/2 avocado<br>Lunch: Salad with grilled chicken<br><br>Tuesday<br>Breakfast: Greek yogurt with berries and a sprinkle of nuts<br>Lunch: Grilled salmon with steamed vegetables<br><br>Wednesday<br>Breakfast: Oatmeal with sliced banana and a teaspoon of honey<br>Lunch: Quinoa salad with mixed vegetables<br><br>Thursday<br>Breakfast: Whole grain toast with almond butter and sliced apple<br>Lunch: Grilled chicken with a side of brown rice and stir-fried greens<br><br>Friday<br>Breakfast: Smoothie with spinach, mixed berries, and protein powder<br>Lunch: Lentil soup with a side of whole grain bread<br><br>Saturday<br>Breakfast: Scrambled tofu with sautéed mushrooms and spinach<br>Lunch: Turkey wrap with whole wheat tortilla and fresh vegetable fillings<br><br>Sunday<br>Breakfast: Chia seed pudding with unsweetened almond milk and sliced peaches<br>Lunch: Baked sweet potato with a mixed bean salad

1 cup of mixed berries (70 calories)
1 cup of black coffee or tea (0 calories)
1 serving of Greek yogurt (150 calories)
Option 2:
Oatmeal made with water (1 cup cooked, 150 calories)
1 banana (105 calories)
1 tablespoon of peanut butter (95 calories)
1 cup of skim milk (90 calories)

Lunch (Approx. 450-500 Calories)
Option 1:
Grilled chicken breast (225 calories)
Mixed green salad with vinaigrette (100 calories)
1 small apple (95 calories)
1 serving of hummus with baby carrots (80 calories)
Option 2:
Chicken and vegetable soup (300 calories)
1 slice of whole-grain bread (100 calories)
1 medium orange (50 calories)

Snack (Approx. 150-200 Calories)
Option 1:
1 small handful of walnuts (100 calories)
1 medium apple (75 calories)
Option 2:
1 tablespoon of peanut butter (95 calories)
1 medium banana (105 calories)

Dinner (Approx. 500-600 Calories)
Option 1:
Grilled salmon (225 calories)
1 cup of brown rice (175 calories)
Steamed green beans (50 calories)
1 side of mixed vegetables (100 calories)
Option 2:
Lean steak (375 calories)
1 cup of mashed potatoes (150 calories)
Steamed asparagus (75 calories)

Snack (Approx. 150-200 Calories)
Option 1:
1 cup of Greek yogurt (150 calories)
A few berries (50 calories)
Option 2:

Steamed Broccoli: 1 cup (55 calories)
Olive or Canola Oil: 1 teaspoon (40 calories)
Evening Snack (183 calories)
Low-Fat Milk or Plant Milk: 1 cup (100 calories)
Honeydew Melon: 1/2 cup (32 calories)
Avocado: 1/6th (51 calories)
Total: 1,560 calories

Nutrient Distribution

Protein Foods: Include grilled chicken, shrimp, and plant-based proteins like tofu.
Dairy or Plant-Based Alternatives: Use low-fat options like Greek yogurt, cottage cheese, and plant milks.
Non-Starchy Vegetables: Incorporate a variety of vegetables such as broccoli, asparagus, and leafy greens.
Starches: Opt for whole grains like oatmeal, whole-grain pasta, and sweet potatoes.
Fruits: Include a mix of berries, bananas, and melons.

Additional Tips
Hydration: Ensure you drink plenty of water throughout the day.
Physical Activity: Try to incorporate some light physical activity, such as walking, to complement your diet plan.
This plan provides a balanced approach to weight loss and nutrition, tailored to your specific needs.

References
1 Australian Dietary Guidelines: Sample Meal Plan for Men
3 1,500-1,600 Calorie Men's Diet Plan
Feel free to adjust portions and food items to better suit your preferences and nutritional needs. Always consult with a healthcare provider or a dietitian before making significant changes to your diet.

Snacks
Snack options: Walnuts, apple slices, baby carrots, hummus, or a small portion of natural peanut butter 1

Dietary Guidelines
Non-starchy vegetables: Incorporate a variety of vegetables such as kale, spinach, broccoli, peppers, and mushrooms into your meals 3.
Protein sources: Include lean proteins like grilled chicken, fish, tofu, and legumes in your diet.
Healthy fats: Avocados, nuts, and olive oil are good sources of healthy fats.
Whole grains: Opt for whole grains like quinoa, brown rice, and oats for sustained energy.
Beverages: Consume non-calorie beverages such as water, green tea, and black coffee 3.

Portion Sizes
It's important to be mindful of portion sizes to manage calorie intake effectively. Consulting with a nutritionist or dietitian can help create a more personalized portion control plan tailored to your specific needs 2.

Remember, sustainable weight loss involves a combination of a balanced diet and regular physical activity. It's advisable to seek professional guidance to ensure your dietary plan aligns with your health goals and lifestyle.

Conclusion
This suggested diet plan aims to provide a balanced, nutritious approach to help you achieve your weight loss goals. Always consult with a healthcare professional or registered dietitian before making significant changes to your diet for personalized guidance and support.

1 small serving of almonds (100 calories)<br>1 medium pear (95 calories)<br>Tips for Success<br><br>Track Your Intake: Use apps or a journal to track your daily calorie intake and ensure you're hitting your targets 3.<br>Stay Hydrated: Drink plenty of water throughout the day.<br>Moderate Physical Activity: Even light activities like walking can support your weight loss goals.<br>Consistent Measurements: Weigh yourself at the same time daily or weekly to monitor progress without stress 1.<br>Remember, this plan is a starting point. Adjust portion sizes and components based on your progress and how you feel. Consulting a healthcare provider or a dietitian can provide personalized advice and adjustments.<br><br>Disclaimer<br>This plan is for informational purposes and should not replace professional medical advice. Always consult with a healthcare provider before making significant changes to your diet or physical activity levels 1.<br><br>By following this structured meal plan and staying consistent, you can work towards reaching your weight goals in a healthy and sustainable manner.		

Chatsonic case 2		
June 27th	June 28th	June 29th
Personalized Diet Plan for a 65-Year-Old Female with Type 2 Diabetes and Reduced Muscle Mass<br><br>Key Considerations:<br><br>Calorie Control: Aim for a moderate caloric intake to support weight management.<br>Carbohydrate Management: Spread carbohydrate intake evenly across meals to manage blood sugar levels.<br>Protein Intake: Include sufficient protein to support muscle mass.<br>Low Sodium: Due to potential kidney concerns indicated by creatinine levels.<br>Fiber-Rich Foods: To help manage blood sugar levels and support digestive health.<br>Healthy Fats: Focus on fats that support heart health.<br><br>Daily Meal Plan:<br>Breakfast:<br>Option 1:<br>One-third cup Grape-Nuts or similar high fiber cereal<br>Half a cup of blueberries<br>1 cup unsweetened almond milk<br>Total Carbs: 41g 1<br>Option 2:<br>1 cup low fat plain Greek yogurt<br>Sweetened with half a mashed banana<br>1 cup strawberries<br>1 tbsp chia seeds<br>Total Carbs: 32g 1<br><br>Lunch:<br>Option 1:<br>Salad: A quarter cup tomatoes, 2 cups spinach, 1 oz cheddar cheese, 1 boiled chopped egg, 2 tbsp yogurt dressing, a quarter cup grapes, 1 tsp pumpkin seeds, 2 oz roasted chickpeas<br>Total Carbs: 47g 1<br>Option 2:<br>Tacos: Two corn tortillas, one-third cup cooked black beans, 1 oz low fat cheese, 4 tbsp avocado, 1 cup coleslaw, salsa as dressing<br>Total Carbs: 76g 1<br><br>Snack:	Certainly! Managing type 2 diabetes and chronic kidney disease (CKD) while addressing reduced muscle mass requires a carefully balanced diet. Here's a proposed diet plan tailored to your needs:<br><br>Diet Plan for Type 2 Diabetes and CKD<br><br>General Guidelines<br><br>Calories: Approximately 1200-1600 calories per day, depending on specific needs and doctor's advice.<br>Carbohydrates: Moderate amount with a focus on low glycemic index (GI) foods.<br>Proteins: Lean proteins that are kidney-friendly.<br>Fats: Healthy fats in moderation.<br>Sodium: Low sodium intake to control blood pressure and fluid retention.<br>Potassium and Phosphorus: Limited due to CKD.<br><br>Sample Daily Meal Plan<br><br>Breakfast<br>Whole-wheat bread: 1 medium slice<br>Jelly: 2 teaspoons (low sugar)<br>Shredded wheat cereal: 1/2 cup<br>Low-fat milk (1%): 1 cup<br>Fruit: 1 medium apple<br>Drink: Coffee or tea (unsweetened)<br><br>Portion Sizes:<br>Bread: 1 slice (28g)<br>Jelly: 2 tsp (10g)<br>Shredded wheat: 1/2 cup (30g)<br>Milk: 1 cup (240ml)<br>Apple: 1 medium (150g)<br><br>Mid-Morning Snack<br>Greek yogurt (low-fat): 1 cup<br>Berries: 1/2 cup (e.g., blueberries, strawberries)<br><br>Portion Sizes:<br>Yogurt: 1 cup (240g)<br>Berries: 1/2 cup (75g)	Based on your characteristics and health conditions, a tailored diet plan can be beneficial. Here's a diet plan incorporating information from various sources to help manage your type 2 diabetes and reduced muscle mass:<br><br>General Dietary Guidelines:<br><br>Healthy Eating for Diabetes and CKD:<br><br>Focus on consuming lots of fruits, veggies, healthy fats, lean protein, and limiting salt, sugar, and refined carbs 1.<br>Your individual carb goal should be based on your age, activity level, and any medicines you take 1.<br><br>Foods to Avoid or Limit:<br>Salt/Sodium: Reduce sodium intake to lower blood pressure and decrease fluid buildup in the body 1.<br>Potassium and Phosphorus: Depending on your kidney disease stage, you may need to reduce potassium, phosphorus, and protein in your diet 1.<br>Protein: Eat the right amount of protein to avoid making your kidneys work harder 1.<br><br>High Potassium Foods:<br>Avoid high potassium foods such as oranges, potatoes, tomatoes, and whole-grain bread 1.<br><br>Protein Intake:<br>Consume the right combination and amount of protein to support muscle health, as both animal and plant foods have protein 1.<br><br>Herbal Supplements:<br>Avoid herbal supplements as they may not be safe for kidney disease 2.<br><br>Portion Sizes and Food Suggestions:<br>Fruits: Berries, grapes, cherries, apples, and plums 1.<br>Veggies: Cauliflower, onions, eggplant, and turnips 1.<br>Proteins: Lean meats (poultry, fish), eggs, unsalted seafood 1.<br>Carbs: White bread, bagels, sandwich buns, unsalted crackers, and pasta 1.<br>Drinks: Water, clear diet sodas, unsweetened tea 1.

Option 1:
1 cup celery with 1 tbsp peanut butter
Total Carbs: 6g 1
Option 2:
One small peach diced into one-third of a cup of 2% fat cottage cheese
Total Carbs: 16g 1

Dinner:
Option 1:
3 oz salmon filet
A medium baked potato with 1 tsp butter
1.5 cups steamed asparagus
Total Carbs: 39g 1
Option 2:
Mediterranean couscous: Two-thirds cup cooked whole wheat couscous, half a cup sautéed eggplant, four sundried tomatoes, five jumbo olives chopped, half a diced cucumber, 1 tbsp balsamic vinegar, fresh basil
Total Carbs: 38g1

Evening Snack:
Option 1:
Half cup vegetable juice
10 stuffed green olives
Total Carbs: 24g 1
Option 2:
One apple with 2 tsp almond butter
Total Carbs: 16g 1
Nutritional Tips:

Carbohydrate Control: Aim to keep total daily carb intake around 130-160 grams, spread evenly across meals.
Protein Intake: Include lean protein sources in each meal to support muscle mass.
Low Sodium Foods: Opt for fresh, unprocessed foods to keep sodium intake low.
Fiber-Rich Vegetables: Non-starchy vegetables like leafy greens, broccoli, and bell peppers should make up half of your plate 2.
Healthy Fats: Include sources like avocados, nuts, and olive oil in moderation 2.
Activity Recommendation:

Increase Physical Activity: Even light exercise such as daily walks can help manage blood sugar levels and improve overall health. Consult with your healthcare provider before starting any new exercise regimen.

Lunch
Roast beef sandwich:
Whole-wheat bread: 2 slices
Lean roast beef: 3 oz
Lettuce and tomato: As desired
Mayonnaise: 1 tsp (low-fat)
Fruit: 1 medium apple
Drink: Water

Portion Sizes:
Bread: 2 slices (56g)
Roast beef: 3 oz (85g)
Lettuce and tomato: As desired (50g total)
Mayonnaise: 1 tsp (5g)
Apple: 1 medium (150g)

Afternoon Snack
Carrot sticks: 1 cup
Hummus: 2 tbsp

Portion Sizes:
Carrot sticks: 1 cup (120g)
Hummus: 2 tbsp (30g)

Dinner
Baked salmon: 3 oz
Vegetable oil: 1.5 tsp (for cooking)
Baked potato: 1 small (3 oz)
Margarine: 1 tsp
Carrots: 1/2 cup (cooked)
Green beans: 1/2 cup (cooked)
Dinner roll: 1 medium
Drink: Unsweetened iced tea
Portion Sizes:

Salmon: 3 oz (85g)
Oil: 1.5 tsp (7ml)
Potato: 1 small (85g)
Margarine: 1 tsp (5g)
Carrots: 1/2 cup (65g)
Green beans: 1/2 cup (60g)
Dinner roll: 1 medium (28g)
Evening Snack

Additional Considerations:
Hydration: Ensure adequate water intake to help lower creatinine levels 2.
Dietary Fiber: Include fruits, vegetables, seeds, and whole grains for their health benefits 3.
Diet Plan Customization:
Caloric Intake: Aim for a calorie intake of 1000-1200 kcal/day to induce weight loss 3.
Meal Composition: Focus on a balanced diet with moderate levels of physical activity to favorably affect parameters of metabolic syndrome 3.
It's crucial to consult with a dietitian to tailor a comprehensive diet plan that considers your specific health conditions and dietary requirements.

Final Note:<br>Consult with a registered dietitian to further customize your diet plan based on your specific health needs and preferences.<br>References:<br>1 Meal plans with carbohydrate counts.<br>2 General dietary recommendations for diabetes management.	Popcorn: 2 1/2 cups (air-popped)<br>Margarine: 1 1/2 tsp<br>Portion Sizes:<br><br>Popcorn: 2 1/2 cups (25g)<br>Margarine: 1 1/2 tsp (7g)<br>Additional Tips<br><br>Hydration: Drink plenty of water throughout the day.<br>Herbs and Spices: Use herbs and spices instead of salt to flavor your food.<br>Consistent Meal Times: Eat at regular intervals to manage blood sugar levels.<br>Monitor Portions: Work with a dietitian to adjust portion sizes as needed.<br>Foods to Avoid<br><br>Saturated Fats: High-fat dairy products, butter, beef, hot dogs, sausage, and bacon.<br>Trans Fats: Processed snacks, baked goods, shortening, and stick margarines.<br>High Cholesterol: Egg yolks, liver, and other organ meats.<br>High Sodium: Limit to no more than 2,300 mg per day, preferably less if advised by your healthcare provider 13.<br>Exercise and Muscle Mass<br>Incorporate light physical activities, such as walking or gentle strength exercises, to improve muscle mass and overall health. Consult your physician before starting any new exercise routine.<br><br>This meal plan is a guideline. It's essential to work closely with your healthcare provider and dietitian to tailor the plan further to suit your specific medical conditions and nutritional needs.	

Perplexity AI case 1		
June 27th	June 28th	June 29th
As a 35-year-old male who is 176 cm tall and weighs 110 kg with a sedentary lifestyle, here is a suggested diet plan to help you lose weight:<br>To lose weight at a healthy rate of 0.5-1% of your body weight per week, you should aim for a daily calorie deficit of 500-1000 calories below your total daily energy expenditure (TDEE). Based on the information provided, your estimated TDEE is around 2,800 calories per day. Therefore, a calorie target of 1,800-2,300 calories per day would be appropriate for steady weight loss.<br><br>Here is a sample 1,800 calorie per day meal plan:<br>Breakfast (350 calories):<br>1 cup (250 ml) of low-fat milk<br>1 cup (40 g) of high-fiber cereal<br>1 medium (120 g) banana<br>Snack (150 calories):<br>1 medium (150 g) apple<br>2 tbsp (30 g) of natural peanut butter<br><br>Lunch (500 calories):<br>4 oz (115 g) grilled chicken breast<br>1 cup (150 g) of steamed broccoli<br>1 cup (180 g) of brown rice<br><br>Snack (200 calories):<br>1 cup (150 g) of low-fat Greek yogurt<br>1/2 cup (75 g) of blueberries<br><br>Dinner (600 calories):<br>6 oz (170 g) of baked salmon<br>1 cup (150 g) of roasted Brussels sprouts<br>1 medium (150 g) sweet potato<br><br>This meal plan provides a balanced intake of protein, carbohydrates, and healthy fats to support your weight loss goals while preserving muscle mass. Be sure to stay hydrated by drinking plenty of water throughout the day. Adjust portion sizes as needed to reach your daily calorie target. Consistency and patience are key for sustainable weight loss.	As a 35-year-old male who is 176 cm tall and weighs 110 kg with a sedentary lifestyle, the following diet plan may be suitable for you:<br>Based on the calorie calculator results, your estimated total daily energy expenditure (TDEE) is around 2,700 calories per day to maintain your current weight. To lose weight at a rate of 0.5-1% of your body weight per week, you should aim for a daily calorie intake of 2,200-2,450 calories.<br>Here is a sample 1,500 calorie meal plan that could work for you:<br><br>Breakfast (350 calories):<br>1 cup (250 ml) of low-fat milk (102 calories)<br>1 cup (40 g) of high-fiber cereal (125 calories)<br>1 medium banana (90 calories)<br><br>Snack (120 calories):<br>1 cup (150 g) of low-fat Greek yogurt (120 calories)<br><br>Lunch (550 calories):<br>4 oz (115 g) grilled chicken breast (225 calories)<br>1 cup (150 g) of steamed broccoli (55 calories)<br>1 cup (180 g) of cooked brown rice (185 calories)<br><br>Snack (100 calories):<br>1 oz (28 g) of raw almonds (100 calories)<br><br>Dinner (480 calories):<br>4 oz (115 g) baked salmon fillet (225 calories)<br>1 cup (150 g) of roasted Brussels sprouts (100 calories)<br>1/2 cup (100 g) of cooked quinoa (105 calories)<br><br>This meal plan provides a balanced intake of protein, carbohydrates, and healthy fats to support weight loss while ensuring adequate nutrition. Remember to stay hydrated by drinking plenty of water throughout the day. Adjust portion sizes as needed to suit your individual calorie needs. Consult a registered dietitian for personalized guidance.	To create a personalized diet plan for you, we need to consider your specific characteristics and lifestyle. Here are some key points to help you achieve a balanced diet:<br><br>Calorie Intake<br>For a sedentary male of your age and height, your daily energy expenditure is approximately 2,400 calories. To achieve weight loss, you should aim for a calorie deficit of 500 calories per day, which means your daily calorie intake should be around 1,900 calories.<br><br>Macronutrient Breakdown<br>A balanced diet should include the following macronutrient proportions:<br><br>Carbohydrates: 55-60% of daily calories (around 260-270 grams)<br>Protein: 15-20% of daily calories (around 57-76 grams)<br>Fat: 20-25% of daily calories (around 38-48 grams).<br>Meal Plan<br>Here is a sample meal plan tailored to your needs:<br><br>Breakfast<br>2 whole-grain toast slices (30 g carbohydrates, 10 g protein, 10 g fat)<br>1 cup cooked oatmeal with fruits and nuts (40 g carbohydrates, 10 g protein, 10 g fat)<br>1 cup mixed berries (20 g carbohydrates, 2 g protein, 1 g fat)<br><br>Mid-Morning Snack<br>1 medium apple (20 g carbohydrates, 0.5 g protein, 0.5 g fat)<br>1 tablespoon almond butter (8 g fat, 4 g protein, 8 g carbohydrates)<br><br>Lunch<br>1 cup cooked brown rice (45 g carbohydrates, 5 g protein, 2 g fat)<br>1 cup mixed vegetables (20 g carbohydrates, 3 g protein, 0.5 g fat)<br>100 g grilled chicken breast (30 g protein, 0 g carbohydrates, 3 g fat)<br><br>Evening Snack<br>1 small banana (25 g carbohydrates, 1 g protein, 0.5 g fat)<br>1 cup low-fat yogurt (30 g carbohydrates, 20 g protein, 0 g fat)<br><br>Dinner<br>1 cup cooked quinoa (30 g carbohydrates, 8 g protein, 4 g fat)<br>1 cup steamed broccoli (5 g carbohydrates, 2 g protein, 0.5 g fat)<br>100 g grilled salmon (35 g protein, 0 g carbohydrates, 10 g fat)

		Tips for a Sedentary Lifestyle<br>Eat whole, minimally processed foods to ensure nutrient-rich meals.<br>Focus on portion control and mindful eating to avoid overconsumption.<br>Incorporate physical activity, such as short walks, to boost your metabolism and overall health.<br>Drink plenty of water throughout the day to stay hydrated and support weight loss.<br>Limit sugary and fatty foods to maintain a balanced diet.<br>By following this meal plan and incorporating these tips, you can achieve a balanced diet tailored to your specific needs and lifestyle.

Perplexity AI case 2		
June 27th	June 28th	June 29th
Here is a suggested diet plan for a 65-year-old female with type 2 diabetes, reduced muscle mass, and a sedentary lifestyle:<br>Calories: 1,500-1,700 per day<br>Carbohydrates: 45-60 grams per meal, 15-30 grams per snack<br>Protein: 20-30 grams per meal, 10-15 grams per snack<br>Fat: 40-60 grams per day<br><br>Meal Plan<br>Breakfast (350-400 calories):<br>1 cup cooked oatmeal with 1/2 cup berries and 2 tbsp sliced almonds<br>1 hard-boiled egg<br><br>Snack (150 calories):<br>1 medium apple with 2 tbsp peanut butter<br><br>Lunch (400-450 calories):<br>4 oz grilled chicken breast<br>1 cup steamed broccoli<br>1/2 cup cooked quinoa<br>1 tbsp olive oil dressing<br><br>Snack (150 calories):<br>1 cup plain Greek yogurt with 1/4 cup blueberries<br><br>Dinner (450-500 calories):<br>4 oz baked salmon<br>1 cup roasted Brussels sprouts<br>1/2 cup mashed sweet potato<br>1 tsp olive oil<br><br>The key aspects of this plan are:<br>Balanced meals with lean protein, non-starchy vegetables, whole grains, and healthy fats<br>Controlled carbohydrate portions to help manage blood sugar<br>Adequate protein intake to help maintain muscle mass<br>Fiber-rich foods like berries, vegetables, and whole grains<br>Healthy fats from sources like nuts, olive oil, and fatty fish<br>This plan provides 1,500-1,700 calories per day, which should support gradual weight loss while meeting your nutritional needs. Be sure to stay hydrated and engage in light physical activity when possible.<br>Consult your doctor or a registered dietitian to further customize this plan to your individual needs.	As a 65-year-old female with type 2 diabetes, reduced muscle mass, and a sedentary lifestyle, the following diet plan may help you manage your condition and potentially lose weight:<br>Carbohydrate Control is Key: Since you have type 2 diabetes, it is crucial to carefully manage your carbohydrate intake. Aim for 45-60 grams of carbs per meal, focusing on complex carbs like whole grains, legumes, and high-fiber fruits and vegetables. Limit simple sugars and refined carbs.<br>Emphasize Lean Proteins and Fiber-Rich Foods: To help maintain muscle mass and control blood sugar, include lean protein sources like poultry, fish, eggs, and legumes at each meal. Also, fill half your plate with non-starchy vegetables and fruits to boost fiber intake.<br>Healthy Fats are Important: Include heart-healthy fats from foods like avocados, nuts, seeds, and olive oil. Avoid trans fats and limit saturated fats.<br><br>Suggested Daily Meal Plan:<br><br>Breakfast (45g carbs): 1 cup oatmeal with 1/2 cup berries, 2 eggs, and 1 tbsp peanut butter.<br>Lunch (45g carbs): 4 oz grilled chicken, 1 cup steamed broccoli, 1/2 cup brown rice, and 1 small apple.<br>Snack (15g carbs): 1 cup plain Greek yogurt with 1/4 cup nuts.<br>Dinner (45g carbs): 4 oz baked salmon, 1 cup roasted Brussels sprouts, and 1/2 cup quinoa.<br>Snack (15g carbs): 1 medium carrot with 2 tbsp hummus.<br>This plan provides around 1,500 calories per day and is tailored to your needs, focusing on portion control, complex carbs, lean proteins, and fiber-rich foods. Be sure to stay hydrated and consider light physical activity, such as walking, to help manage your diabetes and maintain muscle mass.	To create a personalized diet plan for you, considering your height, weight, sedentary lifestyle, type 2 diabetes, and reduced muscle mass, follow these steps:<br><br>Calculate Your Daily Calorie Needs:<br><br>For a sedentary woman of your age and height, your daily calorie needs are approximately 1,800 calories.<br>Macronutrient Breakdown:<br><br>Carbohydrates: Aim for 45% of your daily calories, which is about 200 grams (1,800 * 0.45 = 810 calories / 4 calories per gram of carbohydrates = 202.5 grams). Divide this into 3 meals and 2-3 snacks, ensuring each meal and snack has a balanced mix of carbohydrates, protein, and healthy fats.<br>Protein: Aim for 1.6 grams of protein per kilogram of body weight, which is about 75 grams (93 kg * 1.6 grams/kg). Include protein sources like lean meats, fish, poultry, beans, and dairy products.<br>Fat: Focus on healthy fats like monounsaturated and polyunsaturated fats from sources such as nuts, seeds, avocados, and olive oil. Aim for 30% of your daily calories, which is about 54 grams (1,800 * 0.3 = 540 calories / 9 calories per gram of fat = 60 grams).<br>Meal Plan:<br><br>Breakfast:<br>1 cup cooked oatmeal with fruit and nuts (30 grams carbohydrates, 20 grams protein, 10 grams fat)<br>Lunch:<br>Grilled chicken breast (30 grams protein, 10 grams fat)<br>1 cup mixed greens salad with vegetables and a vinaigrette dressing (10 grams carbohydrates, 10 grams fat)<br>Dinner:<br>3 oz grilled salmon (30 grams protein, 10 grams fat)<br>1 cup cooked quinoa (30 grams carbohydrates, 4 grams protein, 4 grams fat)<br>1 cup steamed broccoli (5 grams carbohydrates, 2 grams protein, 0.5 grams fat)<br>Snacks:<br>1 medium apple with 1 oz almonds (20 grams carbohydrates, 6 grams protein, 16 grams fat)<br>1 cup Greek yogurt with berries (30 grams carbohydrates, 20 grams protein, 0 grams fat)

		Additional Tips:<br><br>Hydration: Drink at least 8 cups of water per day.<br>Portion Control: Use the plate method to ensure balanced portions: half the plate for non-starchy vegetables, one quarter for lean protein, and one quarter for carbohydrates.<br>Fiber: Include high-fiber foods like whole grains, fruits, and vegetables to help manage blood sugar levels and support digestive health.<br>Creatinine Level: Consult with your doctor or a registered dietitian to ensure the diet plan is tailored to your kidney function and creatinine level.<br>Monitoring and Adjustments:<br><br>Blood Sugar: Regularly monitor your blood sugar levels to ensure the diet plan is effective in managing your type 2 diabetes.<br>Weight and Muscle Mass: Monitor your weight and muscle mass regularly. Adjust the diet plan as needed to support weight loss and muscle gain.<br>Remember to consult with your doctor or a registered dietitian to personalize this plan further based on your specific needs and health conditions.

Claude AI case 1		
June 27th	June 28th	June 29th
Based on the information you've provided, I can suggest a general diet plan tailored to your characteristics. However, please note that for a truly personalized diet plan, it's best to consult with a registered dietitian or nutritionist who can take into account your full medical history and specific needs.<br>Given your age, height, weight, and sedentary lifestyle, your goal should likely be weight loss to improve overall health. Here's a suggested diet plan with approximate portion sizes:<br><br>Calories: Aim for about 2000-2200 calories per day for gradual weight loss.<br>Macronutrient split:<br>Protein: 25-30% (125-165g)<br>Carbohydrates: 40-45% (200-250g)<br>Fats: 25-30% (55-75g)<br><br><br>Meal plan:<br><br>Breakfast (500-600 calories):<br>2 whole eggs + 3 egg whites scrambled<br>1 slice whole grain toast with 1 tbsp avocado<br>1 cup mixed berries<br>1 cup unsweetened almond milk<br><br>Mid-morning snack (200 calories):<br>1 medium apple<br>2 tbsp unsalted mixed nuts<br><br>Lunch (500-600 calories):<br>150g grilled chicken breast<br>1 cup brown rice<br>2 cups mixed salad greens with 1 tbsp olive oil and vinegar dressing<br>1 small orange<br><br>Afternoon snack (200 calories):<br>1 cup Greek yogurt (low-fat)<br>1/4 cup granola<br><br>Dinner (500-600 calories):<br>150g baked salmon<br>1 medium sweet potato	Based on your characteristics, I can suggest a general diet plan to help improve your health and potentially lose weight, given that your current Body Mass Index (BMI) indicates obesity. However, please note that for a truly personalized plan, it's best to consult with a registered dietitian or your healthcare provider. Here's a suggested plan:<br>Daily calorie intake: Approximately 2000-2200 calories (for gradual weight loss)<br>Breakfast (400-500 calories):<br><br>2 whole eggs + 3 egg whites, scrambled<br>1 slice whole grain toast<br>1 small apple<br>1 cup unsweetened green tea<br><br>Mid-morning snack (150-200 calories):<br><br>1 small handful (about 1 oz) of mixed nuts<br>1 medium-sized carrot<br><br>Lunch (500-600 calories):<br><br>150g grilled chicken breast<br>1 cup cooked brown rice<br>2 cups mixed salad greens with 1 tbsp olive oil and vinegar dressing<br>1 small orange<br><br>Afternoon snack (150-200 calories):<br><br>1 cup Greek yogurt (low-fat)<br>1/2 cup mixed berries<br><br>Dinner (500-600 calories):<br><br>150g baked salmon<br>1 medium sweet potato<br>2 cups steamed mixed vegetables (broccoli, cauliflower, carrots)<br>1 tsp olive oil for cooking<br><br>Evening snack (if needed, 100-150 calories):<br><br>1 small apple with 1 tbsp peanut butter	Based on the information you've provided, I can suggest a general diet plan tailored to your characteristics. However, please note that for a fully personalized plan, it's best to consult with a registered dietitian or nutritionist. Here's a suggested plan:<br><br>Daily calorie intake: Approximately 2300-2500 calories (for gradual weight loss)<br><br>Breakfast (500-600 calories):<br>- 2 whole eggs + 3 egg whites, scrambled<br>- 1 slice whole grain toast with 1 tbsp avocado<br>- 1 medium apple<br>- 1 cup unsweetened Greek yogurt<br><br>Mid-morning snack (150-200 calories):<br>- 1 oz (28g) mixed nuts<br><br>Lunch (600-700 calories):<br>- 150g grilled chicken breast<br>- 1 cup cooked brown rice<br>- 2 cups mixed salad with 1 tbsp olive oil and vinegar dressing<br>- 1 medium orange<br><br>Afternoon snack (200-250 calories):<br>- 1 medium banana<br>- 2 tbsp hummus with carrot sticks<br><br>Dinner (600-700 calories):<br>- 150g baked salmon<br>- 1 medium sweet potato<br>- 2 cups steamed broccoli<br>- 1 tbsp olive oil (for cooking)<br><br>Evening snack (if needed, 150-200 calories):<br>- 1 cup low-fat cottage cheese with 1/2 cup mixed berries<br><br>Key points:<br>1. Increase protein intake to support muscle maintenance during weight loss.<br>2. Include plenty of vegetables for fiber and nutrients.<br>3. Choose whole grains over refined carbohydrates.<br>4. Include healthy fats from sources like olive oil, avocado, and nuts.

2 cups steamed broccoli<br>1 tsp olive oil for cooking<br><br>Evening snack (if needed, 100-200 calories):<br>1 cup air-popped popcorn OR<br>1 small banana with 1 tbsp peanut butter<br><br><br>Additional recommendations:<br><br><br>Drink at least 8-10 glasses of water daily<br>Limit processed foods, sugary drinks, and alcohol<br>Consider introducing light exercise, such as daily walks, to complement your diet<br><br>Remember, this is a general plan and may need adjustments based on your individual response and preferences. It's also important to introduce changes gradually for sustainable results.	Key points:<br><br>Drink at least 8-10 glasses of water daily.<br>Limit processed foods, sugary drinks, and alcohol.<br>Include a variety of fruits, vegetables, lean proteins, and whole grains.<br>Consider starting a light exercise routine, such as daily walks, to complement your diet.<br><br>Remember, this is a general plan. Your actual needs may vary based on factors like activity level and health conditions.	5. Stay hydrated by drinking at least 8-10 glasses of water daily.<br><br>Remember to gradually increase your physical activity. Start with light exercises like walking for 15-30 minutes daily and progressively increase duration and intensity.<br><br>Would you like me to elaborate on any part of this plan or provide additional information?

Claude AI case 2		
June 27th	June 28th	June 29th
Given your age, height, weight, health conditions, and activity level, I can suggest a general diet plan. However, I must emphasize that it's crucial to consult with your doctor or a registered dietitian before making significant changes to your diet, especially considering your diabetes and kidney function.<br>Here's a suggested diet plan with portion sizes:<br><br>Calories: Aim for about 1600-1800 calories per day for gradual weight loss.<br>Macronutrient distribution:<br>Carbohydrates: 40-45% of total calories (160-200g)<br>Protein: 20-25% of total calories (80-110g)<br>Fat: 30-35% of total calories (60-70g)<br><br>Meal plan:<br><br>Breakfast (400-450 calories):<br>1/2 cup cooked oatmeal (150 calories)<br>1 small apple, sliced (80 calories)<br>1 tablespoon chopped nuts (50 calories)<br>1 hard-boiled egg (70 calories)<br>1 cup unsweetened almond milk (30 calories)<br><br>Mid-morning snack (150-200 calories):<br>1 small orange (60 calories)<br>15 almonds (100 calories)<br><br>Lunch (400-450 calories):<br>3 oz grilled chicken breast (140 calories)<br>1 cup mixed green salad (20 calories)<br>1 tablespoon olive oil-based dressing (80 calories)<br>1/2 cup cooked quinoa (110 calories)<br>1/2 cup steamed broccoli (30 calories)<br><br>Afternoon snack (150-200 calories)<br>1/2 cup low-fat Greek yogurt (65 calories)<br>1/4 cup mixed berries (20 calories)<br>1 tablespoon chia seeds (60 calories)<br><br>Dinner (400-450 calories):<br>3 oz baked salmon (155 calories)	Based on the information you've provided, I can suggest a general diet plan tailored to your characteristics. However, please note that it's crucial to consult with your doctor or a registered dietitian before making significant changes to your diet, especially given your type 2 diabetes and kidney function concerns.<br>Here's a suggested diet plan with approximate portion sizes:<br><br>Calories: Aim for about 1600-1800 calories per day for gradual weight loss.<br>Macronutrient distribution:<br><br>Carbohydrates: 40-45% of total calories (160-200g per day)<br>Protein: 20-25% of total calories (80-110g per day)<br>Fat: 30-35% of total calories (55-70g per day)<br><br>Meal plan:<br><br>Breakfast (400-450 calories):<br><br>1/2 cup cooked steel-cut oats (1/4 cup dry)<br>1 small apple, diced<br>1 tablespoon chopped nuts<br>1 hard-boiled egg<br>1 cup unsweetened almond milk<br><br>Mid-morning snack (150-200 calories):<br><br>1 small orange<br>10-15 almonds<br><br>Lunch (400-450 calories):<br><br>3 oz grilled chicken breast<br>1 cup mixed salad greens<br>1/4 avocado, sliced<br>1/2 cup cooked quinoa<br>1 tablespoon olive oil and lemon juice dressing<br><br>Afternoon snack (150-200 calories):<br><br>1 small Greek yogurt (non-fat, unsweetened)	Given your characteristics and health conditions, it's important to create a diet plan that helps manage your diabetes, supports kidney health, and promotes muscle maintenance. Here's a suggested diet plan with portion sizes tailored to your needs:<br><br>Calorie intake: Aim for about 1600-1800 calories per day for gradual weight loss.<br><br>Macronutrient distribution:<br><br>Carbohydrates: 40-45% of total calories (160-200g per day)<br>Protein: 20-25% of total calories (80-110g per day)<br>Fat: 30-35% of total calories (55-70g per day)<br>Meal plan:<br>Breakfast (400-450 calories):<br>1/2 cup cooked oatmeal (about 150 calories)<br>1 medium apple, sliced (about 95 calories)<br>1 tablespoon chopped nuts (about 50 calories)<br>1 hard-boiled egg (about 70 calories)<br>1 cup unsweetened almond milk (about 30 calories)<br><br>Mid-morning snack (150-200 calories):<br>1 small container (150g) of Greek yogurt (about 130 calories)<br>1/4 cup mixed berries (about 20 calories)<br><br>Lunch (400-450 calories):<br>3 oz grilled chicken breast (about 140 calories)<br>1 cup mixed salad greens (about 10 calories)<br>1 tablespoon olive oil-based dressing (about 120 calories)<br>1/2 cup cooked quinoa (about 110 calories)<br>1/2 cup steamed non-starchy vegetables (about 25 calories)<br><br>Afternoon snack (150-200 calories):<br>1 small apple (about 80 calories)<br>1 oz (about 23) almonds (about 160 calories)<br><br>Dinner (400-450 calories):<br>3 oz baked fish (about 120 calories)<br>1/2 cup cooked brown rice (about 110 calories)<br>1 cup steamed non-starchy vegetables (about 50 calories)<br>1 teaspoon olive oil for cooking (about 40 calories)<br>1 small whole grain roll (about 70 calories)

1/2 cup brown rice (110 calories)<br>1 cup roasted vegetables (carrots, zucchini, bell peppers) (100 calories)<br>1 teaspoon olive oil for cooking (40 calories)<br><br>Evening snack (if needed, 100-150 calories):<br>1 small pear (100 calories)<br><br>Key considerations:<br><br>Carbohydrates: Choose complex carbs with low glycemic index to help manage blood sugar levels.<br>Protein: Include lean protein sources to help maintain muscle mass.<br>Fiber: Aim for 25-30g of fiber daily to aid digestion and blood sugar control.<br>Sodium: Limit sodium intake to support kidney function.<br>Hydration: Drink plenty of water (aim for 8 glasses a day).<br>Portion control: Use measuring cups or a food scale to ensure accurate portions.<br>Meal timing: Eat at regular intervals to help manage blood sugar levels.<br>Physical activity: Gradually increase activity levels as approved by your doctor.<br><br>Remember, this is a general plan and may need adjustments based on your specific needs and medical advice. Regular monitoring of blood sugar levels and kidney function is essential.	1/4 cup mixed berries<br><br>Dinner (400-450 calories):<br><br>3 oz baked salmon<br>1 cup steamed non-starchy vegetables (broccoli, cauliflower, green beans)<br>1/2 cup cooked brown rice<br>1 teaspoon olive oil for cooking<br><br>Evening snack (if needed, 100-150 calories):<br><br>1 small pear<br>1 oz low-fat cheese<br><br>Key points:<br><br>Focus on low glycemic index carbohydrates to help manage blood sugar levels.<br>Include lean proteins in each meal to support muscle mass maintenance.<br>Choose healthy fats like olive oil, avocado, and nuts.<br>Incorporate plenty of non-starchy vegetables for fiber and nutrients.<br>Stay hydrated with water, unsweetened tea, or coffee.<br>Monitor portion sizes using measuring cups or a food scale.<br><br>Remember to:<br><br>Spread your carbohydrate intake throughout the day to help manage blood sugar levels.<br>Choose whole grains, legumes, and vegetables as your primary carbohydrate sources.<br>Include protein with each meal to help maintain muscle mass.<br>Stay consistent with meal timing to help regulate blood sugar.<br>Consider working with a physical therapist or trainer to develop a safe exercise routine to improve muscle mass and overall health.<br><br>This plan is a starting point and may need adjustments based on your individual response and medical supervision. Regular monitoring of your blood sugar levels is essential. Your doctor may also need to adjust your medication based on your dietary changes.	 Evening snack (if needed, 100-150 calories):<br>1 cup raw vegetable sticks (about 25 calories)<br>2 tablespoons hummus (about 70 calories)<br><br>Additional recommendations:<br>Choose low glycemic index carbohydrates to help manage blood sugar levels.<br>Include lean proteins with each meal to support muscle mass maintenance.<br>Incorporate healthy fats from sources like olive oil, nuts, and fatty fish.<br>Aim for at least 25-30g of fiber per day from whole grains, vegetables, fruits, and legumes.<br>Limit sodium intake to support kidney health (aim for less than 2300mg per day).<br>Stay hydrated with water, unsweetened tea, or other non-caloric beverages.<br>Consider consulting with a registered dietitian for personalized guidance.<br>Remember, this is a general plan and may need adjustments based on your specific needs and preferences. It's crucial to work closely with your healthcare provider and a registered dietitian to fine-tune this plan, especially considering your diabetes and kidney function. They can help monitor your progress and make necessary adjustments to ensure the diet is meeting your health goals.<br><br>Would you like me to elaborate on any part of this diet plan or provide more information about managing diabetes through diet?

Pi AI case 1		
June 27th	June 28th	June 29th
Given your information, here is a suggested balanced diet plan with portion sizes tailored to your characteristics, which can aid in weight loss while providing essential nutrients:<br><br>Breakfast (around 7:30 AM):<br>Whole grain bread (2 slices)<br>Avocado (1/4 of a medium avocado)<br>Scrambled eggs (2 medium eggs)<br>Greek yogurt (200g)<br>Mixed berries (1/2 cup)<br><br>Morning Snack (around 10:30 AM):<br>Almonds (20 almonds)<br>Apple (1 medium)<br><br>Lunch (around 1:00 PM):<br>Grilled Chicken Breast (150g)<br>Quinoa (1/2 cup cooked)<br>Steamed Broccoli (1 cup)<br>Mixed Green Salad (2 cups)<br>Olive oil (1 tablespoon)<br>Afternoon Snack (around 4:30 PM):<br>Baby Carrots (1 cup)<br>Hummus (2 tablespoons)<br><br>Dinner (around 7:30 PM):<br>Baked Salmon (150g)<br>Sweet Potato (1 medium)<br>Steamed Spinach (1 cup)<br>Olive oil (1 tablespoon)<br><br>Evening Snack (around 9:30 PM):<br>Strawberries (1/2 cup)<br>Dark Chocolate (1 ounce)<br>Ensure that you drink plenty of water throughout the day to stay hydrated and maintain optimal bodily functions. Try to incorporate at least 30 minutes of light physical activity, such as walking or cycling, into your daily routine to support your weight loss goals. You can make modifications based on your personal preferences, as long as you keep the portion sizes and nutritional balance in check. It's always best to consult a dietician or medical professional before starting a new diet plan, especially if you have specific health concerns or requirements.	Certainly! Based on the information you provided, here's a balanced, calorie-controlled diet plan with portion sizes for a target of 1,800-2,000 calories per day:<br><br>Breakfast<br>Oatmeal with fruit: ½ cup cooked oatmeal with 1 cup of mixed berries, a sprinkle of chia seeds, and 1 tablespoon of honey.<br>Coffee or tea (optional)<br><br>Mid-morning snack<br>Greek yogurt with nuts: ½ cup nonfat Greek yogurt with 1 tablespoon chopped almonds.<br><br>Lunch<br>Grilled chicken salad: 3-4 oz grilled chicken breast on a bed of mixed greens with ½ cup mixed vegetables (e.g., bell peppers, carrots, cucumbers), and a lemon vinaigrette dressing.<br>1 small whole-grain roll with 1 teaspoon of olive oil.<br><br>Afternoon snack<br>Fruit and nut butter: 1 medium apple or pear with 1 tablespoon almond or peanut butter.<br><br>Dinner<br>Grilled salmon with vegetables: 4-5 oz grilled salmon with 1 cup roasted or steamed vegetables (e.g., broccoli, asparagus, zucchini).<br>½ cup cooked quinoa or brown rice.<br><br>Evening snack<br>Light popcorn: 2 cups air-popped popcorn, spritzed with olive oil and seasoned with a pinch of salt and pepper.<br><br>Drinks<br>Water: 8-10 glasses per day.<br>Unsweetened tea or coffee (optional): limit to 1-2 cups per day.<br><br>Active choices for snacking<br>Baby carrots with hummus<br>Celery sticks with peanut butter<br>Hard-boiled eggs<br><br>Recommended physical activity	Absolutely, here's a sample diet plan based on your provided information. Please note that this is just an example and can be adjusted based on your preferences and needs.<br>Meal Plan for Weight Loss (Sedentary Lifestyle, Male, 35-year-old, 176 cm tall, 110 kg)<br>Total Caloric Intake: 1900-2200 calories per day<br><br>Breakfast (Approx. 350 calories):<br>1 cup whole grain cereal with 1 cup low-fat milk (or milk substitute)<br>1 small fruit (e.g., banana, apple, orange)<br><br>Mid-morning Snack (Approx. 150 calories):<br>1 small serving of almonds (approx. 25 almonds)<br><br>Lunch (Approx. 500 calories):<br>Large salad with mixed greens, ½ sliced avocado, cherry tomatoes, 1 sliced hard-boiled egg, 2 tablespoons low-fat dressing<br>1 cup cooked brown rice<br><br>Mid-afternoon Snack (Approx. 150 calories):<br>1 cup low-fat Greek yogurt with 1 cup mixed berries<br><br>Dinner (Approx. 650 calories):<br>4 ounces grilled chicken breast<br>1 medium sweet potato, cooked<br>1 cup steamed broccoli with garlic<br><br>Evening Snack (Optional, 100-200 calories):<br>2 cups low-fat popcorn<br><br>This sample meal plan focuses on balanced nutrition, incorporating lean protein, complex carbohydrates, healthy fats, and plenty of fruits and vegetables. Remember that portion control is key, and it's essential to pay attention to portion sizes to ensure you are not overeating. Additionally, staying properly hydrated is important. Aim for at least 2-3 liters of water per day.<br>Lastly, incorporating some form of physical activity into your daily routine can significantly help your weight loss efforts and improve overall health. Even a daily 30-minute walk can make a difference.

	In addition to following the diet plan, incorporating regular physical activity will help you achieve your weight loss goals. As you have a sedentary lifestyle, start with low to moderate intensity exercises like walking, cycling, or swimming for 30 minutes at least 4-5 times a week. Gradually increase the duration and intensity as you progress. Remember to consult your healthcare provider before making any significant changes to your diet or starting a new exercise regimen. Good luck with your weight loss journey!	

Pi AI case 2		
June 27th	June 28th	June 29th
To develop a diet plan that suits your needs, it's important to consider your age, medical conditions, and lifestyle. Given your type 2 diabetes, we'll focus on a balanced diet that is lower in simple carbohydrates to manage blood sugar levels and incorporates more protein to help improve muscle mass. Since you have slightly elevated creatinine levels, it's advisable to not consume excessive amounts of protein.<br><br>Breakfast:<br>1/2 cup oatmeal or whole grain toast with 1 tablespoon peanut butter<br>1/2 cup plain low-fat Greek yogurt with berries or 1 small fruit<br>1 boiled or poached egg (optional)<br>8 ounces of water<br><br>Mid-morning snack:<br>1 small piece of fruit or 1 small serving of low-fat string cheese<br>8 ounces of water<br><br>Lunch:<br>1 serving lean protein (chicken breast, tofu, fish, or lean beef) about the size of a deck of cards<br>1 cup non-starchy vegetables (broccoli, spinach, bell peppers, or carrots)<br>1/2 cup whole grain rice or sweet potato<br>8 ounces of water<br><br>Afternoon snack:<br>Handful of unsalted nuts or seeds<br>8 ounces of water<br><br>Dinner:<br>1 serving lean protein (chicken breast, tofu, fish, or lean beef) about the size of a deck of cards<br>1 cup non-starchy vegetables (broccoli, spinach, bell peppers, or carrots)<br>1/2 cup whole grain pasta or a slice of whole grain bread<br>8 ounces of water<br><br>Evening snack:<br>1 small serving of low-fat Greek yogurt with berries<br>8 ounces of water<br><br>Daily fluid intake: Aim to consume at least 64 ounces of water throughout the day.<br>Foods to avoid: Refined carbohydrates, sugary snacks, and highly	Given your specific requirements, I have designed a sample daily diet plan. This diet will prioritize foods that are low in phosphorus, potassium, and sodium, and it will provide approximately 1,200-1,300 calories per day. I recommend consulting a registered dietitian to get a more comprehensive plan, as they can consider your specific needs and goals.<br><br>Breakfast:<br>1/2 cup oatmeal cooked with water<br>1/4 cup blueberries<br>1/4 cup non-dairy milk<br><br>Mid-morning Snack:<br>1 small apple<br>1 tablespoon peanut butter<br><br>Lunch:<br>2 oz grilled chicken breast<br>1/2 cup cooked brown rice<br>1/2 cup steamed carrots<br>1/2 cup steamed green beans<br><br>Afternoon Snack:<br>1/4 cup raw almonds<br><br>Dinner:<br>3 oz baked salmon<br>1/2 cup quinoa<br>1/2 cup steamed broccoli<br>1/2 cup steamed cauliflower<br><br>Evening Snack:<br>1 small orange<br>This plan includes nutrient-dense foods that are suitable for individuals with diabetes and slightly reduced renal function. Please remember that portion sizes may vary depending on your individual needs.	Considering your age, weight, height, sedentary lifestyle, type 2 diabetes, creatinine level, and reduced muscle mass, here's a sample diet plan with portion sizes that can help you manage your condition and support your health. Please note that this is a general recommendation and should be adjusted based on your individual needs and preferences. Always consult your healthcare provider before making significant changes to your diet.<br>Breakfast:<br><br>1/2 cup (40g) of oats cooked in 1 cup (240 ml) of low-fat milk<br>1 serving of low-sugar fruit, such as berries or apples (1 cup or around 150g)<br><br>Snack:<br>1 small carrot (60g) or celery stick (40g) with 2 tablespoons (30g) of hummus<br><br>Lunch:<br>3 oz (85g) of grilled or baked lean protein (chicken, fish, or tofu)<br>1 cup (180g) of cooked quinoa or brown rice<br>1 cup (150g) of steamed non-starchy vegetables (e.g., broccoli, green beans, spinach)<br><br>Snack:<br>1 small apple (120g) with 1 tablespoon (15g) of natural peanut butter<br><br>Dinner:<br>3 oz (85g) of grilled or baked lean protein (chicken, fish, or tofu)<br>1 cup (180g) of cooked quinoa or brown rice<br>1 cup (150g) of steamed non-starchy vegetables (e.g., broccoli, green beans, spinach)<br>1/2 cup (70g) of steamed starchy vegetables (e.g., sweet potato, corn)<br><br>Evening snack (if needed):<br>1/2 cup (120ml) of low-fat Greek yogurt topped with 1/4 cup (30g) of blueberries<br>This sample diet plan includes foods rich in fiber, lean protein, and essential nutrients while limiting added sugars, saturated fats, and sodium. Aim for a total caloric intake of around 1,400 to 1,600 calories per day. As you manage your diabetes, it's crucial to maintain stable blood sugar levels by eating regular meals and snacks throughout the

processed foods.<br>Physical activity: Incorporating light exercise such as walking for 20-30 minutes a day can help improve insulin resistance and muscle mass. However, be sure to consult your physician before starting any new physical activity.<br>Please note that this is a general meal plan and should be adjusted based on your individual preferences and in consultation with your doctor or a registered dietitian.		day. Be sure to stay hydrated and incorporate physical activity into your routine as recommended by your healthcare provider.

You.com case 1		
June 27th	June 28th	June 29th
Diet Plan for a Sedentary 35-Year-Old Male<br><br>Based on your specific characteristics, here is a suggested diet plan with portion sizes tailored to your needs:<br><br>1. Caloric Intake: As a sedentary individual, your caloric intake should be adjusted to promote weight loss. Aim for a daily caloric deficit of 500-1000 calories to lose weight gradually and sustainably.<br><br>2. Macronutrient Distribution: Your diet should consist of a balanced distribution of macronutrients:<br>- Carbohydrates: Consume complex carbohydrates such as whole grains, legumes, and vegetables. Aim for 45-65% of your daily caloric intake.<br>- Protein: Include lean sources of protein such as poultry, fish, tofu, and legumes. Aim for 10-35% of your daily caloric intake.<br>- Fats: Choose healthy fats from sources like avocados, nuts, seeds, and olive oil. Aim for 20-35% of your daily caloric intake.<br><br>3. Portion Sizes: While portion sizes can vary depending on individual needs, here are some general guidelines:<br>- Protein: Aim for 3-4 ounces (85-113 grams) of protein per meal. This is roughly the size of a deck of cards.<br>- Carbohydrates: Include 1/2 to 1 cup (85-170 grams) of cooked grains or starchy vegetables per meal.<br>- Vegetables: Fill half of your plate with non-starchy vegetables like leafy greens, broccoli, and peppers.<br>- Fruits: Include 1-2 servings of fruit per day. One serving is typically a medium-sized fruit or 1 cup of berries.<br>- Fats: Limit added fats to 1-2 tablespoons per meal. This can include oils, nuts, or seeds.<br><br>4. Meal Plan Example:<br>- Breakfast: Start your day with a balanced meal, such as a vegetable omelet with whole-grain toast.<br>- Snack: Enjoy a piece of fruit and a handful of nuts as a mid-morning snack.<br>- Lunch: Have a salad with grilled chicken or tofu, mixed vegetables, and a light dressing.<br>- Snack: Opt for Greek yogurt with berries and a sprinkle of granola in the afternoon.<br>- Dinner: Prepare a balanced meal with a palm-sized portion of lean	Diet Plan for a Sedentary 35-Year-Old Male<br><br>Based on your specific characteristics, including your age, height, weight, and sedentary lifestyle, here are some general guidelines for a diet plan:<br><br>1. Caloric Intake: To achieve weight loss, it is important to create a calorie deficit. Start by calculating your daily caloric needs using a reliable online calculator or consulting with a healthcare professional. This will give you an estimate of the number of calories you should consume each day to lose weight.<br><br>2. Macronutrient Distribution: Aim for a balanced distribution of macronutrients, including carbohydrates, proteins, and fats. A general guideline is to consume around 45-65% of your calories from carbohydrates, 10-35% from protein, and 20-35% from fats. Adjust these percentages based on your personal preferences and any dietary restrictions you may have.<br><br>3. Portion Sizes: Portion control is crucial for weight management. Here are some general guidelines for portion sizes:<br><br>- Proteins: Include lean sources of protein such as chicken, turkey, fish, tofu, and legumes. Aim for a portion size of around 3-4 ounces per meal.<br>- Carbohydrates: Choose complex carbohydrates like whole grains, fruits, and vegetables. Aim for a portion size of around 1/2 to 1 cup per meal.<br>- Fats: Include healthy fats from sources like avocados, nuts, seeds, and olive oil. Aim for a portion size of around 1-2 tablespoons per meal.<br><br>4. Meal Frequency: Consider dividing your daily caloric intake into smaller, more frequent meals throughout the day. This can help regulate blood sugar levels and prevent overeating.<br><br>5. Hydration: Stay hydrated by drinking plenty of water throughout the day. Limit sugary beverages and alcohol, as they can contribute to weight gain.<br><br>6. Nutrient-Dense Foods: Focus on consuming nutrient-dense foods that provide essential vitamins, minerals, and fiber. Include a variety of fruits, vegetables, whole grains, lean proteins, and healthy fats in your diet.	#### Diet Plan for a Sedentary 35-Year-Old Male<br><br>Based on your specific characteristics, here is a suggested diet plan with portion sizes tailored to your needs:<br><br>1. Caloric Intake: To maintain or lose weight, it is important to monitor your calorie intake. The Centers for Disease Control and Prevention (CDC) recommends assessing your weight using the Body Mass Index (BMI). You can use an online calorie calculator to estimate your daily caloric needs. This will help you determine the appropriate calorie intake for your goals.<br><br>2. Balanced Diet: A nutritious, balanced diet is essential for overall health. It is important to include a variety of foods from different food groups to ensure you are getting all the necessary nutrients. Here are some general guidelines:<br><br>- Protein: Include lean sources of protein such as chicken, turkey, fish, tofu, beans, and lentils. Aim for about 20-30 grams of protein per meal.<br>- Carbohydrates: Choose complex carbohydrates like whole grains, fruits, vegetables, and legumes. Limit refined carbohydrates and sugary foods.<br>- Fats: Include healthy fats from sources like avocados, nuts, seeds, and olive oil. Limit saturated and trans fats.<br>- Fiber: Include high-fiber foods like whole grains, fruits, vegetables, and legumes to promote digestion and satiety.<br>- Vitamins and Minerals: Ensure you are getting a variety of fruits and vegetables to meet your vitamin and mineral needs.<br><br>3. Portion Sizes: Portion control is important for weight management. Here are some general guidelines for portion sizes:<br><br>- Protein: Aim for a palm-sized portion (about 3-4 ounces) of lean protein per meal.<br>- Carbohydrates: Include about 1/2 to 1 cup of cooked grains or starchy vegetables per meal.<br>- Fruits and Vegetables: Aim for 1-2 cups of vegetables and 1-2 servings of fruit per meal.<br>- Fats: Use healthy fats in moderation. For example, use 1-2 tablespoons of olive oil or a small handful of nuts/seeds per meal.<br><br>4. Meal Plan Example: Here is a sample meal plan for a sedentary 35-

protein, a serving of whole grains or starchy vegetables, and a side of non-starchy vegetables.<br>- Snack: Choose a small portion of a healthy snack, such as carrot sticks with hummus or a small handful of almonds.<br><br>Remember to consult with a healthcare professional or registered dietitian for personalized advice tailored to your specific needs and goals.	7. Limit Processed Foods: Minimize your intake of processed foods, which are often high in added sugars, unhealthy fats, and sodium. Opt for whole, unprocessed foods whenever possible.<br><br>8. Seek Professional Guidance: It is always a good idea to consult with a registered dietitian or healthcare professional who can provide personalized guidance based on your specific needs and goals.<br><br>Remember, these are general guidelines, and it is important to tailor your diet plan to your individual needs and preferences. It is also recommended to incorporate regular physical activity into your lifestyle for overall health and weight management.<br><br>Please note that the information provided here is based on general knowledge and should not replace personalized advice from a healthcare professional.	year-old male:<br><br>- Breakfast:<br>- 1 cup of cooked oatmeal with 1 tablespoon of almond butter and a handful of berries.<br>- 1 boiled egg.<br>- 1 cup of green tea.<br><br>- Lunch:<br>- Grilled chicken breast (4 ounces) with a side of roasted vegetables (1-2 cups).<br>- 1/2 cup of cooked quinoa.<br>- 1 small apple.<br><br>- Snack:<br>- 1 small handful of almonds.<br>- 1 medium-sized carrot.<br><br>- Dinner:<br>- Baked salmon (4 ounces) with a side of steamed broccoli (1-2 cups).<br>- 1/2 cup of cooked brown rice.<br>- 1 small orange.<br><br>- Snack:<br>- 1 small Greek yogurt with a sprinkle of granola.<br><br>5. Hydration: Don't forget to stay hydrated by drinking plenty of water throughout the day. Aim for at least 8 cups (64 ounces) of water per day.<br><br>Remember, this is just a general guideline. It is always recommended to consult with a healthcare professional or a registered dietitian for personalized advice tailored to your specific needs and goals.<br><br>I hope this helps you get started on your journey towards a healthier lifestyle!

You.com case 2		
June 27th	June 28th	June 29th
Diet Plan for a 65-Year-Old Female with Type 2 Diabetes and Reduced Muscle Mass<br><br>Based on the information provided, here is a suggested diet plan tailored to your characteristics:<br><br>1. Consult with a healthcare professional: Before making any significant changes to your diet, it's important to consult with a healthcare professional, such as a registered dietitian or your doctor. They can provide personalized advice based on your specific needs and medical history.<br><br>2. Weight management: Since you mentioned that you have a sedentary lifestyle and are overweight, weight management is an important aspect of your diet plan. Achieving and maintaining a healthy weight can help improve your diabetes management and overall health.<br><br>- Work with your healthcare professional to determine a healthy weight range for you, taking into consideration factors such as age, sex, muscle mass, bone mass, and lifestyle.<br>- Aim for gradual and sustainable weight loss, typically 0.5-1 kg (1-2 pounds) per week.<br>- Focus on creating a calorie deficit by consuming fewer calories than you burn through physical activity and daily living.<br>- Keep in mind that weight loss should be accompanied by lifestyle changes, including regular physical activity and portion control.<br><br>3. Carbohydrate control: Carbohydrate control is crucial for managing blood sugar levels in individuals with type 2 diabetes. Here are some guidelines:<br><br>- Choose complex carbohydrates that are high in fiber, such as whole grains, legumes, fruits, and vegetables.<br>- Limit or avoid refined carbohydrates and sugary foods and beverages, as they can cause rapid spikes in blood sugar levels.<br>- Distribute your carbohydrate intake evenly throughout the day to help maintain stable blood sugar levels.<br>- Consider working with a registered dietitian to determine your specific carbohydrate needs and to learn about carbohydrate counting.<br><br>4. Protein intake: Protein is important for maintaining muscle mass and supporting overall health. Here are some recommendations:	Diet Plan for a 65-Year-Old Female with Type 2 Diabetes and Reduced Muscle Mass<br><br>Based on your characteristics and health conditions, here is a suggested diet plan with portion sizes tailored to your needs:<br>Consult with your doctor: Before making any significant changes to your diet, it's important to consult with your doctor or a registered dietitian who can provide personalized advice based on your specific health needs.<br>Weight management: Since you mentioned that you have reduced muscle mass and are overweight, it may be beneficial to focus on weight management. Achieving a healthy weight can help improve your overall health and manage your diabetes. Your doctor can help determine a healthy weight range for you, taking into consideration factors such as age, sex, muscle mass, bone mass, and lifestyle<br>.<br>Caloric intake: To manage your weight, it's important to balance your calorie intake. The National Institutes of Health (NIH) suggests that women get at least 1,200 calories a day unless supervised by doctors<br>. However, it's important to note that individual calorie needs may vary, so it's best to consult with a healthcare professional to determine the appropriate calorie range for you.<br>Carbohydrates: Focus on consuming complex carbohydrates that are high in fiber, such as whole grains, legumes, fruits, and vegetables. These foods can help regulate blood sugar levels and provide essential nutrients. It's important to monitor your carbohydrate intake and spread it throughout the day to avoid blood sugar spikes. A registered dietitian can help you determine the appropriate carbohydrate servings for each meal.<br>Protein: Protein is important for maintaining muscle mass and promoting satiety. Include lean sources of protein in your diet, such as skinless poultry, fish, tofu, legumes, and low-fat dairy products. The Protein Requirements Calculator can help determine your protein requirements based on your activity level.<br><br>Fats: Choose healthy fats, such as avocados, nuts, seeds, and olive oil, while limiting saturated and trans fats. Healthy fats can help improve heart health and provide essential nutrients.<br><br>Meal distribution: Aim for three balanced meals and two to three small snacks throughout the day. This can help regulate blood sugar levels	Diet Plan for a 65-Year-Old Female with Type 2 Diabetes and Reduced Muscle Mass<br><br>Based on the information provided, I can suggest a diet plan tailored to your characteristics. However, please note that it's important to consult with a healthcare professional or a registered dietitian before making any significant changes to your diet. They can provide personalized advice and take into account any specific medical conditions or dietary restrictions you may have.<br><br>1. Caloric Intake: It's important to maintain a calorie intake that supports weight management and blood sugar control. The number of calories you need will depend on various factors, including your age, weight, height, activity level, and overall health. A general guideline for women is to consume at least 1,200 calories per day, unless supervised by a doctor.<br><br>2. Carbohydrates: Focus on consuming complex carbohydrates that have a lower impact on blood sugar levels. These include whole grains, legumes, vegetables, and fruits. Aim to include a variety of colorful vegetables in your meals to ensure a good intake of vitamins, minerals, and fiber.<br><br>3. Protein: Protein is important for maintaining muscle mass and supporting overall health. Include lean sources of protein such as poultry, fish, tofu, legumes, and low-fat dairy products. The amount of protein you need will depend on your individual needs and can be determined in consultation with a healthcare professional or registered dietitian.<br><br>4. Fats: Choose healthy fats such as avocados, nuts, seeds, and olive oil. Limit saturated and trans fats found in fried foods, processed snacks, and fatty cuts of meat. Incorporate omega-3 fatty acids from sources like fatty fish (salmon, mackerel) or flaxseeds.<br><br>5. Portion Sizes: Portion control is important for managing weight and blood sugar levels. Consider using smaller plates and bowls to help control portion sizes. A registered dietitian can provide guidance on appropriate portion sizes for your specific needs.<br><br>6. Hydration: Stay well-hydrated by drinking water throughout the day.

- Consume lean sources of protein, such as poultry, fish, tofu, legumes, and low-fat dairy products.<br>- Aim for a moderate protein intake, typically around 0.8-1.2 grams of protein per kilogram of body weight per day.<br>- Protein intake can be adjusted based on your individual needs, so it's best to consult with a healthcare professional or registered dietitian.<br><br>5. Healthy fats: Include healthy fats in your diet, such as avocados, nuts, seeds, and olive oil. These fats can help improve satiety and provide essential nutrients.<br><br>6. Portion control: Pay attention to portion sizes to avoid overeating. Here are some general guidelines:<br><br>- Use smaller plates and bowls to help control portion sizes.<br>- Fill half of your plate with non-starchy vegetables, one-quarter with lean protein, and one-quarter with whole grains or starchy vegetables.<br>- Be mindful of calorie-dense foods and limit their portion sizes.<br><br>7. Meal timing: Consider spreading your meals and snacks throughout the day to help manage blood sugar levels. Aim for regular meal times and avoid skipping meals.<br><br>8. Hydration: Stay hydrated by drinking plenty of water throughout the day. Limit sugary beverages and alcohol, as they can affect blood sugar levels and overall health.<br><br>Remember, these are general guidelines, and it's important to work with a healthcare professional or registered dietitian to create a personalized diet plan that suits your specific needs and preferences. They can provide you with more detailed guidance and support to help you achieve your health goals.<br><br>I hope this information is helpful! Let me know if you have any other questions.	and prevent overeating.<br><br>Hydration: Stay hydrated by drinking plenty of water throughout the day. Limit sugary beverages and alcohol, as they can affect blood sugar levels and contribute to weight gain.<br><br>Monitoring and support: Regularly monitor your blood sugar levels and work closely with your healthcare team to adjust your diabetes management plan as needed. Consider seeking support from a registered dietitian who specializes in diabetes management to help you create a personalized meal plan and provide ongoing guidance.<br><br>Remember, this is a general guideline, and it's important to consult with your healthcare team for personalized advice based on your specific health needs and goals.	Adequate hydration is important for overall health and can support kidney function.<br><br>7. Meal Timing: Consider spreading your meals and snacks throughout the day to help manage blood sugar levels. Aim for three balanced meals and 1-2 snacks, if needed. Avoid skipping meals, as this can lead to fluctuations in blood sugar levels.<br><br>Remember, it's important to work closely with a healthcare professional or registered dietitian to develop a personalized diet plan that takes into account your specific needs and medical conditions. They can provide guidance on portion sizes, meal timing, and any necessary modifications to support your health goals.<br>I hope this information is helpful! Let me know if you have any other questions.

<table>
<tr><th colspan="3">Zenochat case 1</th></tr>
<tr><th>June 27th</th><th>June 28th</th><th>June 29th</th></tr>
<tr>
<td>Hello! I'm Zeno, an AI assistant here to help you with a personalized diet plan. Let's take a look at your characteristics:

Age: 35 years old
Height: 176 cm
Weight: 110 kg
Lifestyle: Sedentary
Based on this information, I would recommend the following diet plan:

Recommended Daily Calorie Intake: 2,100 - 2,300 calories

Macronutrient Breakdown:

Carbohydrates: 45-55% of total calories (225-275 g)
Protein: 25-30% of total calories (130-170 g)
Fat: 20-25% of total calories (50-65 g)
Meal Plan:

Breakfast (400-450 calories):
1 cup of oatmeal with 1 cup of low-fat milk and 1 tbsp of honey
2 eggs cooked in 1 tsp of olive oil
1 slice of whole-grain toast with 1 tbsp of peanut butter

Lunch (500-550 calories):
4 oz grilled chicken breast
1 cup of steamed broccoli
1 cup of brown rice
1 small apple

Snack (200-250 calories):
1 cup of Greek yogurt with 1/2 cup of mixed berries
1 oz of almonds

Dinner (500-550 calories):
5 oz of baked salmon
1 cup of roasted sweet potatoes
1 cup of sautéed spinach
1 small pear

This plan provides a balanced diet with a focus on whole, nutrient-dense foods. Please remember to stay hydrated and adjust portion sizes as needed based on your individual needs and goals. If you have any</td>
<td>Hello! I'm Zeno, an AI assistant. Based on the information you provided, here's a suggested diet plan tailored to your characteristics:

Calorie Target: To achieve a moderate weight loss of 0.5-1 kg per week, your daily calorie target should be around 1,800-2,000 calories.

Macronutrient Breakdown:

Protein: 25-30% of total calories (112-150 g per day)
Carbohydrates: 40-50% of total calories (180-225 g per day)
Fats: 20-30% of total calories (40-67 g per day)
Suggested Meal Plan:
Breakfast (400 calories):
1 cup of oatmeal with 1/2 cup of berries and 1 tablespoon of honey
2 eggs cooked in 1 teaspoon of olive oil

Lunch (500 calories):
4 oz grilled chicken breast
1 cup of steamed broccoli
1 cup of brown rice

Dinner (600 calories):
6 oz baked salmon
1 cup of roasted sweet potatoes
1 cup of sautéed spinach

Snacks (300 calories):
1 apple with 2 tablespoons of natural peanut butter
1 cup of plain Greek yogurt with 1/2 cup of mixed berries
This meal plan provides a balanced intake of nutrients, emphasizes lean proteins, vegetables, and whole grains, and should help you achieve your weight loss goals. Remember to stay hydrated and consider incorporating light to moderate exercise, such as brisk walking, into your routine. Let me know if you have any other questions!</td>
<td>Hello, I'm Zeno, the AI assistant. Based on the information you provided, here is a suggested diet plan tailored to your characteristics:

Calorie Intake: To start losing weight at a healthy pace, I recommend aiming for a calorie deficit of around 500-1000 calories per day. This would put your daily calorie intake in the range of 1800-2300 calories.

Macronutrient Breakdown:

Protein: 25-30% of total calories, which would be 112-162 grams per day
Carbohydrates: 40-50% of total calories, which would be 180-275 grams per day
Fats: 20-30% of total calories, which would be 40-77 grams per day
Meal Plan (based on 2100 calories per day):

Breakfast (400 calories): 2 eggs, 1 cup oatmeal, 1 apple
Lunch (650 calories): 6 oz grilled chicken, 1 cup brown rice, 1 cup steamed broccoli, 1 small salad with 2 tbsp dressing
Snack (250 calories): 1 cup Greek yogurt, 1 cup berries
Dinner (800 calories): 8 oz salmon, 1 cup roasted sweet potatoes, 1 cup sautéed spinach
Portion Sizes:

Protein: 4-6 oz per meal
Carbohydrates: 1/2 - 1 cup per meal
Vegetables: 1 cup per meal
Fruits: 1 medium piece or 1 cup
Dairy: 1 cup
Remember to stay hydrated by drinking plenty of water throughout the day. It's also important to incorporate some form of physical activity, even if it's just a daily 30-minute walk, to support your weight loss goals.

This is a general guideline, and you may need to adjust the calorie intake and macronutrient ratios based on your individual needs and progress. I recommend consulting a registered dietitian for a more personalized plan.</td>
</tr>
</table>

specific dietary restrictions or concerns, please consult with a registered dietitian for personalized guidance.		

Zenochat case 2		
June 27th	June 28th	June 29th
Hello, I'm Zeno, the assistant. Based on the information you provided, here is a suggested diet plan tailored to your characteristics:<br><br>General Guidelines:<br>Focus on a balanced diet with a emphasis on nutrient-dense, high-fiber foods<br>Moderate carbohydrate intake to help manage blood sugar levels<br>Ensure adequate protein intake to maintain and build muscle mass<br>Stay hydrated by drinking plenty of water<br><br>Recommended Daily Intake:<br>Calories: 1,500 - 1,700 kcal<br>Carbohydrates: 150-200 g (40-45% of total calories)<br>Protein: 90-105 g (20-25% of total calories)<br>Fat: 50-60 g (30-35% of total calories)<br><br>Meal Suggestions:<br>Breakfast (300-350 kcal):<br>1 cup oatmeal with 1/2 cup berries and 2 tbsp nuts<br>2 eggs scrambled with 1 cup spinach and 1 slice whole grain toast<br><br>Lunch (400-450 kcal):<br>4 oz grilled salmon, 1 cup roasted sweet potatoes, 1 cup steamed broccoli<br>1 cup mixed greens salad with 2 tbsp olive oil and balsamic vinegar dressing<br><br>Dinner (400-450 kcal):<br>4 oz baked chicken breast, 1 cup brown rice, 1 cup steamed green beans<br>1 cup lentil soup with 2 slices whole grain bread<br><br>Snacks (150-200 kcal):<br>1 cup plain Greek yogurt with 1/2 cup berries<br>1 small apple with 2 tbsp almond butter<br>Please note that this is a general guideline, and you should consult with your healthcare provider or a registered dietitian to ensure the plan is tailored to your specific needs and preferences.	Hello, this is Zeno, the assistant. I'd be happy to provide you with a diet plan tailored to your specific characteristics and health conditions. However, please note that I am an AI assistant and my recommendations are based on general guidelines, not a substitute for personalized medical advice from your healthcare provider. I suggest discussing this plan with your doctor to ensure it aligns with your individual needs and treatment plan.<br><br>Given your age, height, weight, and sedentary lifestyle, along with your type 2 diabetes and reduced muscle mass, the following diet plan may be beneficial:<br><br>Calorie target: 1,600-1,800 calories per day Macronutrient breakdown:<br><br>Carbohydrates: 45-55% of total calories, focusing on high-fiber, complex carbohydrates<br>Protein: 20-25% of total calories, to help maintain and rebuild muscle mass<br>Fat: 25-30% of total calories, emphasizing healthy unsaturated fats<br>Suggested meal plan:<br>Breakfast (350-400 calories):<br>1 cup (150g) of oatmeal with 1/2 cup (125ml) of low-fat milk and 1/2 cup (75g) of berries<br>1 hard-boiled egg<br><br>Lunch (450-500 calories):<br>4 oz (115g) of grilled chicken breast<br>1 cup (150g) of steamed broccoli<br>1/2 cup (75g) of cooked brown rice<br>1 small apple<br><br>Dinner (450-500 calories):<br>4 oz (115g) of baked salmon<br>1 cup (150g) of roasted sweet potato<br>1 cup (150g) of mixed green salad with 2 tbsp (30ml) of olive oil-based dressing<br>1 small orange<br><br>Snacks (150-200 calories):<br>1 cup (240ml) of plain, low-fat Greek yogurt with 1/4 cup (40g) of mixed nuts<br>1 hard-boiled egg and 1 small pear	Hello, I'm Zeno, an AI assistant. Given your health characteristics, here is a suggested diet plan tailored to your needs:<br><br>Dietary Considerations:<br><br>Type 2 Diabetes: Focus on foods with low glycemic index to help manage blood sugar levels. Avoid added sugars and refined carbs.<br>Reduced Muscle Mass: Include adequate protein intake to help maintain and rebuild muscle mass.<br>Kidney Function: Moderate protein and monitor sodium/potassium intake due to elevated creatinine levels.<br>Sedentary Lifestyle: Reduce overall calorie intake to promote gradual weight loss.<br><br>Suggested Meal Plan:<br>Breakfast:<br>1 cup oatmeal with 1/2 cup berries and 2 tbsp. nuts<br>2 eggs scrambled with 1/2 cup spinach<br><br>Lunch:<br>4 oz grilled chicken breast<br>1 cup steamed broccoli<br>1/2 cup brown rice<br><br>Snack:<br>1 medium apple<br>1 oz cheddar cheese<br><br>Dinner:<br>4 oz baked salmon<br>1 cup roasted sweet potato<br>1 cup sautéed zucchini<br><br>Portion Sizes:<br>Protein: 4-6 oz per meal<br>Non-starchy Veggies: 1 cup per meal<br>Starchy Carbs: 1/2 cup per meal<br>Healthy Fats: 1-2 tbsp per meal<br>Fruits: 1 medium piece per day<br>This plan provides approximately 1,500 calories per day with a balanced macronutrient profile suitable for your health conditions and goals.<br>Remember to stay hydrated and consult your doctor or a registered

	Remember to stay hydrated by drinking water throughout the day. Additionally, consider incorporating light physical activity, such as a 30-minute walk, to help maintain muscle mass and improve overall health.<br><br>Again, please consult with your doctor to ensure this plan is appropriate for your individual needs and make any necessary adjustments. I'm happy to provide further guidance as needed.	dietitian for personalized guidance. Let me know if you have any other questions!
