# Supplementary material for: Comparison of the Accuracy, Completeness, Reproducibility, and Consistency of Different AI Chatbots in Providing Nutritional Advice: An Exploratory Study
Source: J Clin Med. 2024 Dec 20;13(24):7810. doi: 10.3390/jcm13247810 (PMC11677083; doi:10.3390/jcm13247810)
Supplement: Supplementary file 1 [file jcm-13-07810-s001.zip › Supplementary Table S3.pdf]

Table S3a. Evaluation of the accuracy, completeness, and reproducibility of Case 1

		Accuracy (maximum score 20)					Completeness (maximum score 7)					Reproducibility (maximum score 12)				
		Expert 1	Expert 2	Expert 3	Mean± SD	% Accuracy	Expert 1	Expert 2	Expert 3	Mean± SD	% Completeness	Expert 1	Expert 2	Expert 3	Mean ± SD	% Reproducibility
**ChatGPT 4.0**	Day 1	12	13	12			5	6	5							
	Day 2	14	14	15	12.2±2.3	61.1	7	7	7	6.1±0.8	87.3	7	5	6	6.0±1.0	50.0
	Day 3	11	7	12			6	6	6							
**ChatGPT 3.5**	Day 1	14	16	14			7	4	6							
	Day 2	12	12	8	13.4±2.5	67.2	6	6	5	6.1±1.0	87.3	8	4	7	6.3±2.1	52.8
	Day 3	16	14	15			7	7	7							
**Gemini**	Day 1	9	9	13			3	3	6							
	Day 2	6	8	9	8.0±2.8	40.0	4	3	4	3.9±0.9	55.6	10	5	8	7.7±2.5	63.9
	Day 3	6	3	9			4	4	4							
**Copilot**	Day 1	2	5	2			2	2	2							
	Day 2	4	4	7	4.2±1.6	21.1	3	2	4	3.0±1.0	42.9	5	8	7	6.7±1.5	55.5
	Day 3	5	4	5			4	4	4							
**Chatsonic**	Day 1	8	6	8			5	5	4							
	Day 2	6	4	8	6.2±2.1	31.1	5	5	5	4.6±0.5	65.1	10	12	9	10.3±1.5	86.1
	Day 3	6	2	8			4	4	4							
**Perplexity AI**	Day 1	6	6	5			4	4	3							
	Day 2	2	6	4	8.0±4.9	40.0	4	4	3	4.8±1.7	68.3	7	8	6	7.0±1.0	58.3
	Day 3	14	14	15			7	7	7							
**Claude**	Day 1	14	8	12			7	7	6							
	Day 2	10	8	11	10.2±2.0	51.1	6	6	6	6.0±0.7	85.7	9	7	8	8.0±1.0	66.7
	Day 3	10	11	8			5	6	5							
**Pi AI**	Day 1	5	5	5			4	4	3							
	Day 2	13	10	11	8.2±2.9	41.1	6	5	5	4.7±0.9	66.7	7	9	9	8.3±1.1	69.4
	Day 3	8	8	9			5	5	5							
**You.com**	Day 1	5	6	6			4	5	5							
	Day 2	7	6	6	5.7±0.7	26.7	3	4	3	4.0±0.7	57.1	9	7	6	7.3±1.5	61.1
	Day 3	5	5	5			4	4	4							
**ZenoChat**	Day 1	10	6	9			5	4	5							
	Day 2	11	8	12	9.7±2.1	48.3	6	7	6	5.6±0.9	79.4	8	7	9	8.0±1.0	66.7
	Day 3	12	8	11			6	6	5							

SD= Standand deviation

Table S3b. Evaluation of the accuracy, completeness, and reproducibility of Case 2

		Accuracy (maximum score 32)					Completeness (maximum score 10)					Reproducibility (maximum score 18)				
		Expert 1	Expert 2	Expert 3	Mean ± SD	% Accuracy	Expert 1	Expert 2	Expert 3	Mean ± SD	% Completeness	Expert 1	Expert 2	Expert 3	Mean ± SD	% Reproducibility
**ChatGPT 4.0**	Day 1	21	13	19			8	8	9							
	Day 2	11	11	10	14.8±4.8	46.2	7	7	7	7.8±0.7	77.8	9	9	9	9.0±0	50.0
	Day 3	22	16	10			8	8	8							
**ChatGPT 3.5**	Day 1	7	9	8			5	5	5							
	Day 2	16	15	15	12.5±3.7	39.2	8	8	8	6.7±1.3	66.7	12	14	13	13.0±1.0	72.2
	Day 3	17	12	14			7	7	7							
**Gemini**	Day 1	15	10	13			4	4	4							
	Day 2	13	12	13	13.3±1.7	41.7	4	4	4	4.0±0	40	17	17	17	17.0±0	94.4
	Day 3	16	14	14			4	4	4							
**Copilot**	Day 1	6	6	6			3	2	3							
	Day 2	10	12	10	7.9±2.3	24.6	4	2	4	2.3±1.2	23.3	12	17	11	13.3±3.2	74.1
	Day 3	8	6	7			1	1	1							
**Chatsonic**	Day 1	10	11	10			5	5	5							
	Day 2	17	15	16	13.1±2.6	41.0	8	7	8	6.4±1.2	64.6	8	12	8	9.3±2.3	51.8
	Day 3	14	12	13			6	7	7							
**Perplexity AI**	Day 1	12	7	12			7	7	7							
	Day 2	10	8	11	10±1.9	31.2	7	8	7	7.0±0.5	70	11	14	11	12.0±1.7	66.7
	Day 3	11	8	11			6	7	7							
**Claude**	Day 1	16	8	16			8	9	8							
	Day 2	14	6	13	13.1±4.7	41.0	8	6	8	7.8±0.8	77.8	13	14	13	13.3±0.6	72.2
	Day 3	19	8	18			8	7	8							
**Pi AI**	Day 1	6	4	8			6	5	6							
	Day 2	9	4	6	7.5±3.3	23.6	5	5	5	6.2±1.4	62.2	8	11	8	9.0±1.7	50.0
	Day 3	14	6	11			8	8	8							
**You.com**	Day 1	13	7	12			4	4	4							
	Day 2	11	6	9	9.4±2.4	29.5	4	4	4	3.8±0.4	37.8	13	14	13	13.3±0.6	74.1
	Day 3	11	7	9			3	4	3							
**ZenoChat**	Day 1	14	7	13			6	6	6							
	Day 2	18	12	16	12.0±3.7	37.5	7	7	7	6.8±0.7	67.8	11	13	11	11.7±1.1	64.8
	Day 3	10	7	11			7	8	7							
